# Associations between gut microbiota and sleep: a two-sample, bidirectional Mendelian randomization study

**DOI:** 10.3389/fmicb.2023.1236847

**Published:** 2023-08-14

**Authors:** Jun Wu, Baofu Zhang, Shengjie Zhou, Ziyi Huang, Yindong Xu, Xinwu Lu, Xiangtao Zheng, Dong Ouyang

**Affiliations:** ^1^Department of Vascular Surgery, The Second Affiliated Hospital of Wenzhou Medical University, Wenzhou, China; ^2^Department of Obstetrics and Gynecology, Taizhou Women and Children’s Hospital of Wenzhou Medical University, Taizhou, Zhejiang, China; ^3^Department of Vascular Surgery, Shanghai Ninth People’s Hospital Affiliated to Shanghai Jiao Tong University School of Medicine, Shanghai, China

**Keywords:** sleep, gut microbiota, Mendelian randomization, instrumental variable, causal relationship

## Abstract

**Introduction:**

Previous research has reported that the gut microbiota performs an essential role in sleep through the microbiome–gut–brain axis. However, the causal association between gut microbiota and sleep remains undetermined.

**Methods:**

We performed a two-sample, bidirectional Mendelian randomization (MR) analysis using genome-wide association study summary data of gut microbiota and self-reported sleep traits from the MiBioGen consortium and UK Biobank to investigate causal relationships between 119 bacterial genera and seven sleep-associated traits. We calculated effect estimates by using the inverse-variance weighted (as the main method), maximum likelihood, simple model, weighted model, weighted median, and MR-Egger methods, whereas heterogeneity and pleiotropy were detected and measured by the MR pleiotropy residual sum and outlier method, Cochran’s Q statistics, and MR-Egger regression.

**Results:**

In forward MR analysis, inverse-variance weighted estimates concluded that the genetic forecasts of relative abundance of 42 bacterial genera had causal effects on sleep-associated traits. In the reverse MR analysis, sleep-associated traits had a causal effect on 39 bacterial genera, 13 of which overlapped with the bacterial genera in the forward MR analysis.

**Discussion:**

In conclusion, our research indicates that gut microbiota may be involved in the regulation of sleep, and conversely, changes in sleep-associated traits may also alter the abundance of gut microbiota. These findings suggest an underlying reciprocal causal association between gut microbiota and sleep.

## 1. Introduction

Sleep disorders have become a global public health issue, affecting approximately 15–30% of adults and causing significant burdens on quality of life, as well as occupational, psychological, and economic well-being (Ohayon, 2002; Morin et al., 2006). In modern society, owing to the negative effects of modern work patterns and screen time, the prevalence of sleep disorders and circadian rhythm disorders is growing (Thomée et al., 2011; Jehan et al., 2017; Murdock et al., 2017; Harknett et al., 2021; Di et al., 2022). Moreover, recent studies have indicated that insufficient sleep and sleep disturbances are correlated with countless adverse outcomes (Itani et al., 2017; Jike et al., 2018; Sun et al., 2020; Winer et al., 2021; Sun et al., 2022; Han et al., 2023). However, the molecular mechanisms underlying sleep–wake cycles are unclear. Early research focused on the central nervous system’s role in sleep regulation and dysregulation (Raven et al., 2018; Van Someren, 2021; Sulaman et al., 2023). However, sleep is not only regulated by the central system but also affected by signals from peripheral tissues. Recently, researchers have focused on specific interactions between circadian rhythm processes and the gut microbiome.

The gut microbiome is a highly complex microbial community that may directly or indirectly participate in the regulation of the sleep–wake cycle through the microbiome–gut–brain axis (Neuman et al., 2015; Sen et al., 2021; Wang et al., 2022). Moreover, dietary composition, rhythms of feeding, and loss of the microbiome influence the composition of the gut cycling transcriptome and the expression of rhythm genes, independently and together (Leone et al., 2015; Zhang et al., 2023). For example, intraperitoneal injection of components of bacterial cell walls or bacteria-derived metabolites, such as lipopolysaccharides, lipoteichoic acid, and butyrate, were found to increase non-REM sleep in mice (Szentirmai and Krueger, 2014; Szentirmai et al., 2019, 2021). Some randomized controlled trials have suggested that *Bifidobacteria* and *Lactobacillaceae* may help enhance sleep quality, especially sleep induction, and relieve subclinical signs of anxiety and depression (Nishida et al., 2019; Ho et al., 2021; Lee et al., 2021). Additionally, sleep disturbances are associated with the disruption of gut bacteria, which results in a dysfunctional colonic barrier and the development of intestinal illnesses (Benedict et al., 2016; Gao et al., 2023). However, owing to the existence of confounding factors, such as lifestyle, diet, and age (Rinninella et al., 2019), and the limitations of experimental ethics, it is difficult to carry out randomized controlled trials (RCT) to uncover the causal association between gut microbiota and sleep. Moreover, previous observational studies were not robust because they contained small numbers of participants and the direction of the effects was difficult to judge. As a result, it is unclear whether gut microbiota and sleep disturbances are causally related.

In genetic epidemiology, Mendelian randomization is a method that involves using genetic variants to compose instrumental variables (IVs) of traits to investigate the causal relationship between traits and outcomes. Since genetic variants are generally randomly assigned at meiosis and are not affected by disease states, MR analysis can minimize common confounding factors, avoid confounding factors measurement error, and overcome reverse causation (Smith and Ebrahim, 2003; Davey Smith and Hemani, 2014).

We conducted a two-sample, bidirectional MR study using single nucleotide polymorphisms (SNPs) from the most recent genome-wide association studies (GWASs) to identify whether gut microbiota affect sleep disturbances and whether such associations are directional.

## 2. Materials and methods

### 2.1. Description of study design

**Figure 1:**
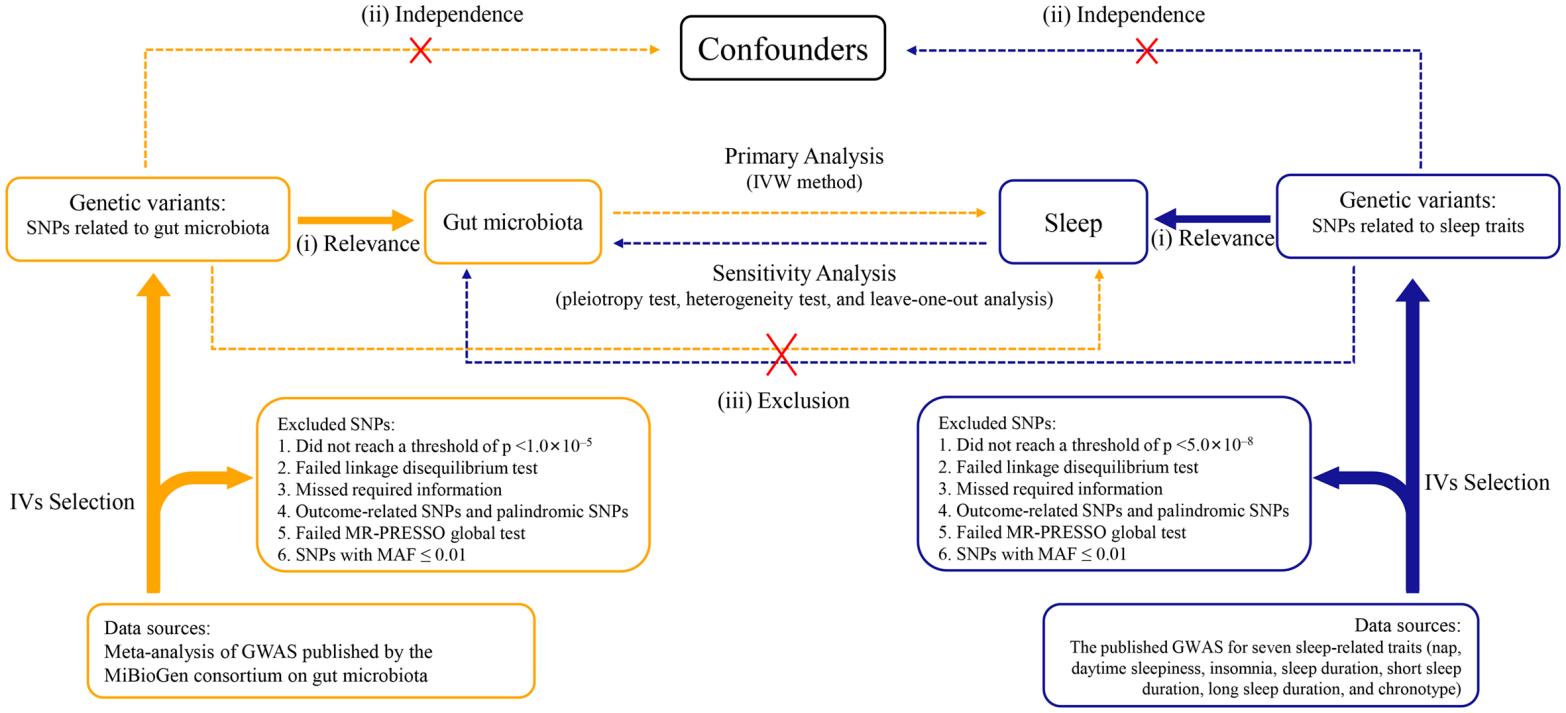
Study design of the bidirectional Mendelian randomization study on the associations of gut microbiota and sleep. IVs, instrumental variables. GWAS, genome-wide association studies. SNPs, single nucleotide polymorphisms. MR-PRESSO, Mendelian randomization pleiotropy residual sum and outlier. MAF, minor allele frequency. IVW, inverse variance weighted.

A two-sample, bidirectional MR design was performed to uncover the possible causal effects of gut microbiota on sleep-related traits (Figure 1). There were three core assumptions that genetic variants had to meet in order to be included as IVs in our study (Swerdlow et al., 2016; Davies et al., 2018): (i) relevance—the relationship between genetic variants and exposure was robust; (ii) independence—the genetic variants were independent of confounding factors affecting exposure and outcome; and (iii) exclusion restriction—the genetic variants influenced the risk of the outcome through exposure rather than other potential pathways. The forward MR analyses considered gut microbiota as the exposure and each sleep phenotype as the outcome. By contrast, reverse MR analyses took each sleep phenotype as the exposure and gut microbiota as the outcome. We used a two-sample MR computational model to investigate if there were bidirectional causal relationships between gut microbiota and sleep traits. Finally, several sensitivity analyses (the heterogeneity test, the pleiotropy test, and leave-one-out analysis) were performed sequentially.

### 2.2. Data sources

The GWAS summary statistics used in our study were compiled and are shown in Supplementary Table S1. The individuals from the data sources we used for the MR analysis were primarily of European ancestry.

#### 2.2.1. Gut microbiota

GWAS summary data for intestinal bacteria were assessed from the MiBioGen consortium. This consortium conducted the largest GWAS of the intestinal microbiome. The GWAS gathered whole-genome genotyping data from 18,340 participants (24 cohorts) as well as the 16S rRNA genes from participant’s fecal microbiomes. Then, using three distinct variable regions (V1-V2, V3-V4, and V4) of the 16S rRNA gene, the study profiled the composition of intestinal microbial species. By performing microbiome trait loci mapping, genetic variants were identified that affected the relative abundance or presence of nine phyla, 16 classes, 20 orders, 35 families, and 131 genera (included 12 unknown genera). Finally, we included 119 genera taxa for our bidirectional MR study.

#### 2.2.2. Sleep-related traits

GWAS summary data for seven sleep-related traits, namely daytime napping (*n* = 452,633), daytime sleepiness (*n* = 452,071), insomnia (*n* = 386,533), sleep duration (*n* = 446,118), long sleep duration (*n* = 339,926), short sleep duration (*n* = 411,934), and chronotype (*n* = 403,195) were obtained from United Kingdom Biobank. Sleepiness and napping during the day are recognized as related clinical features of the attenuated arousal continuum (Dashti et al., 2021). Moreover, daytime napping may result from a lack of sleep at night or underlying poor health (Sayón-Orea et al., 2013; Celis-Morales et al., 2017), which makes causal inferences difficult in observational studies. Excessive daytime sleepiness is a main sign of chronic sleep deficiency and several primary sleep disorders; it affects 10–20% of the population (Ohayon, 2008; Cohen et al., 2010). Insomnia is a common disorder, and up to 33% of the population experience transient insomnia symptoms at any given time (Morin et al., 2015). Sleep duration, as judged by self-reported data, is traditionally regarded as a continuous variable and divided into two distinct categories: short sleep duration (<7 h/night) and long sleep duration (≥9 h/night). In addition, we excluded extreme cases of sleep duration of less than 3 h or more than 18 h. Chronotype is determined by individual tendencies to sleep earlier or later, often referred to as circadian preference. Chronotype is generally treated as a continuous variable, but to provide interpretable OR in this GWAS study, a binary phenotype was also defined by using the same data field as for chronotype (Jones et al., 2019).

### 2.3. Selection of instrumental variables

For forward MR analysis, a sufficient number of SNPs need to be included as IVs for subsequent sensitivity analysis and horizontal pleiotropic detection. Therefore, we extracted SNPs closely related to the gut microbiota from the published data, with *p* < 1.0 × 10^−5^ as the primary filter. For the reverse MR analysis, SNPs were associated with sleep-related traits and reached the conventional GWAS significance threshold (*p* < 5.0 × 10^−8^). Then, to make sure that the IVs applied to exposure were independent, European sample data from the 1,000 Genomes project was used as the reference panel; SNPs in linkage disequilibrium (r2 < 0.001, clumping window = 10,000 kb) were excluded. We extracted SNPs associated with exposure for each outcome, and where exposed SNPs were not available they were discarded. After harmonizing exposure as well as outcome SNPs, we excluded palindromic SNPs, outliers eliminated by the MR pleiotropy residual sum and outlier (MR-PRESSO) global test, and SNPs with minor allele frequency ≤ 0.01.

### 2.4. Mendelian randomization analysis

For MR analysis, multiple statistical models including inverse-variance weighted (IVW), simple model, weighted model, weighted median, maximum likelihood method, and MR-Egger regression were utilized to estimate the potential bidirectional causal relationships between gut microbiota and sleep traits. We used the random-effects IVW method as the principal statistical method, and the overall estimate obtained by this method is equivalent to weighted linear regression for Wald estimates for each SNP, regardless of intercept (Burgess et al., 2013). However, in the presence of horizontal pleiotropic SNPs, the IVW results would be severely biased (Burgess et al., 2016). Therefore, we used the MR-Egger method, which provides a valid test for causal effects consistent with the IVW method, after excluding SNPs that are directly related to the results or have horizontal pleiotropy (Bowden et al., 2015). The maximum likelihood method, similar to the IVW method, can provide results with a smaller standard error than IVW in the absence of heterogeneity or horizontal pleiotropy (Hartwig et al., 2017). Complementary analyses using the simple model, weighted model, and weighted median method were used as supplements to IVW. The weighted median method gives a credible estimate even if up to half of the results come from invalid SNPs (Bowden et al., 2016). When the largest number of similar individual SNPs causal effect estimates are from efficient SNPs, the weighted model was consistent even if SNPs were invalid (Hartwig et al., 2017). And the simple mode is an unweighted mode of the empirical density function of causal estimation (Hemani et al., 2018).

### 2.5. Sensitivity analysis

The intercept term of MR-Egger regression was used to determine the presence of pleiotropy. When the intercept term approaches zero, it suggests that there is no horizontal pleiotropy for the SNP used in the bidirectional MR analysis. In addition, we performed the MR-PRESSO global test to judge horizontal pleiotropy. Furthermore, we used Cochran’s Q statistics and funnel plots to assess the heterogeneity of the IVW method. Moreover, to evaluate whether a single SNP impacted the main causal association, we conducted the “leave-one-out” analysis by eliminating each SNP in turn. In order to appraise the strength of IVs, we computed the F-statistic according to the following formula: $F=\frac{R^{2}\times \left(N-1-K\right)}{\left(1-R^{2}\right)\times K}$ . All the bidirectional MR analyses were performed using the two-sample MR (version 0.5.6) R packages in R (version 4.2.2). Finally, when the IVW-derived *p*-value <0.05 and the estimates of all methods were in the same direction, we considered the results of MR analysis to be nominally significant. In addition, taking into account multiple hypothesis testing, we set the *p*-value for Bonferroni correction in the forward MR analysis to 0.05/9 (0.0055) and in the reverse MR analysis to 0.05/119 (4.20 × 10^−4^).

## 3. Results

### 3.1. Causal effects of gut microbiota on sleep-related traits

Based on the selection criteria of the IVs, we selected 460 SNPs for 42 bacterial taxa to uncover the potential causal associations between gut microbiota and sleep-related traits in the forward MR analysis. rs9393920 (in MR analysis of *Oscillibacter* on sleep duration) and rs736744 (in MR analysis of *Oxalobacter* on daytime sleepiness) were detected as outliers by MR-PRESSO and removed. All the F-statistics of IVs were larger than 10, which indicated weak instrument bias was unlikely. Supplementary Table S2 shows the details of the selected IVs of 42 bacterial taxa, including Beta, standard error, and *p*-values.

**Figure 2:**
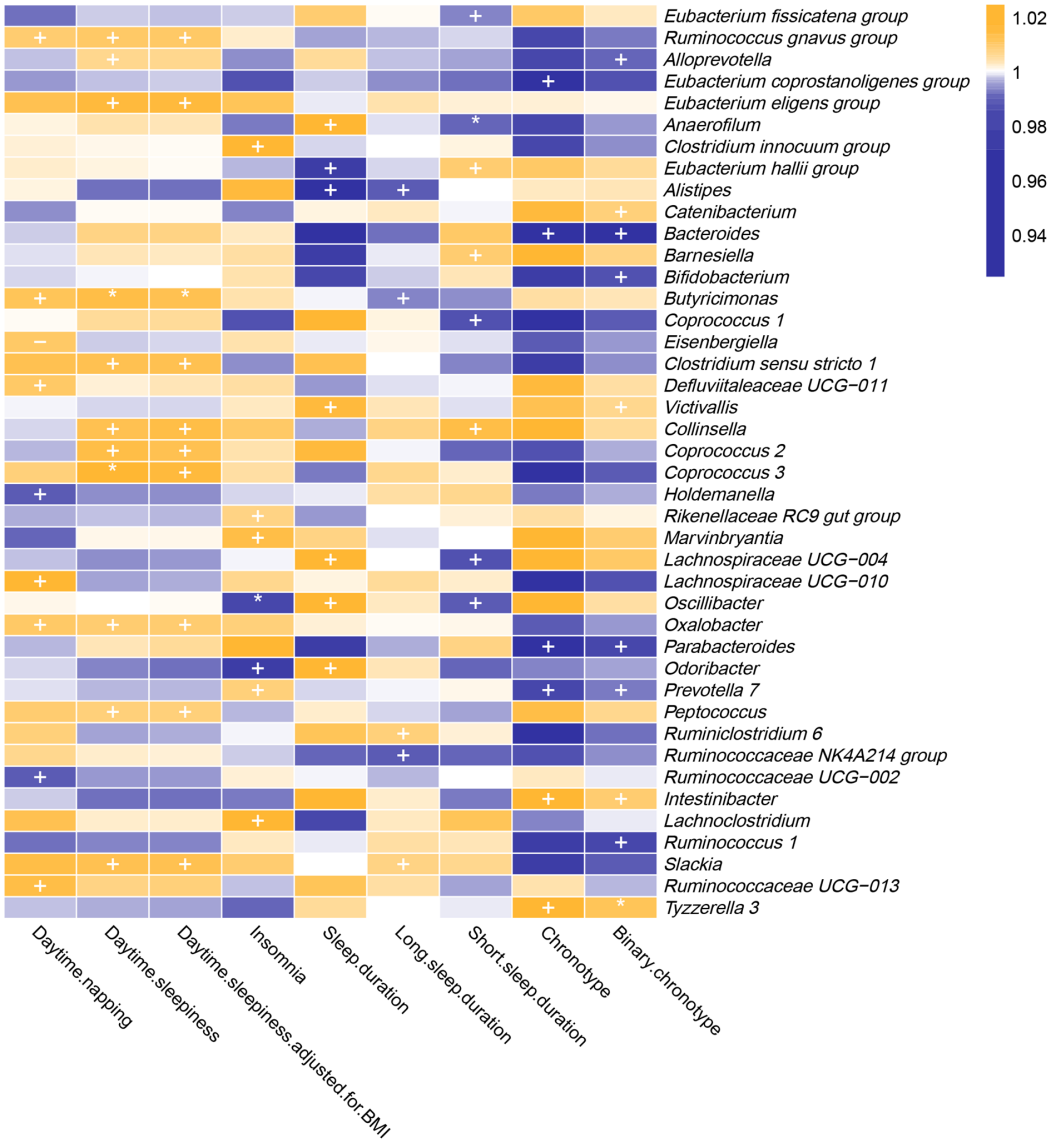
In the forward MR analysis, IVW estimates from 42 bacterial genera on seven sleep-associated traits (daytime napping, daytime sleepiness, insomnia, sleep duration, long sleep duration, short sleep duration and chronotype). The color of each block represents the OR of every MR analysis (blue, OR < 1; orange, OR > 1). *p*-values of <0.05 were marked with “+,” *p*-values of <0.0055 were marked with “*” and *p*-values of <0.05 with potential pleiotropy were marked with “−”.

As shown in Figure 2 and Supplementary Table S3, genetically predicted abundances of nine genera were associated with daytime napping, 11 genera were associated with daytime sleepiness, seven genera were associated with insomnia, seven genera were associated with sleep duration, five genera were associated with long sleep duration, eight genera were associated with short sleep duration, and six genera were associated with chronotype, according to the estimates of the IVW method.

#### 3.1.1. Daytime napping

The genetic forecast of abundance ratio of *Holdemanella* (OR: 0.989, 95% CI: 0.979–0.998, *p* = 0.020) and *Ruminococcaceae* UCG-002 (OR: 0.990, 95% CI: 0.981–0.999, *p* = 0.032) showed a negative correlation with daytime napping. The genetic forecast of abundance ratio of seven intestinal flora genera was positively correlated with daytime napping, specifically, *Ruminococcus* (*gnavus group*) (OR: 1.010, 95% CI: 1.000–1.019, *p* = 0.041), *Defluviitaleaceae* UCG-011 (OR: 1.011, 95% CI: 1.000–1.021, *p* = 0.046), *Oxalobacter* (OR: 1.011, 95% CI: 1.001–1.021, *p* = 0.034), *Eisenbergiella* (OR: 1.011, 95% CI: 1.001–1.021, *p* = 0.040), *Butyricimonas* (OR: 1.013, 95% CI: 1.001–1.024, *p* = 0.035), *Ruminococcaceae* UCG-013 (OR: 1.015, 95% CI: 1.000–1.029, *p* = 0.049), and *Lachnospiraceae* UCG-010 (OR: 1.017, 95% CI: 1.001–1.034, *p* = 0.033).

#### 3.1.2. Daytime sleepiness

The IVW estimates suggested that the genetic forecast of abundance ratio of 11 intestinal flora genera was positively associated with daytime sleepiness, specifically, *Alloprevotella* (OR: 1.007, 95% CI: 1.000–1.014, *p* = 0.048), *Peptococcus* (OR: 1.009, 95% CI: 1.002–1.015, *p* = 0.008), *Oxalobacter* (OR: 1.014, 95% CI: 1.007–1.021, *p* = 2.79E-05), *Ruminococcus* (*gnavus group*) (OR: 1.010, 95% CI: 1.002–1.018, *p* = 0.011), *Collinsella* (OR: 1.013, 95% CI: 1.000–1.025, *p* = 0.045), *Slackia* (OR: 1.013, 95% CI: 1.001–1.026, *p* = 0.006), *Clostridium sensu stricto 1* (OR: 1.014, 95% CI: 1.002–1.025, *p* = 0.021), *Coprococcus 2* (OR: 1.014, 95% CI: 1.002–1.026, p = 0.020), *Coprococcus 3* (OR: 1.018, 95% CI: 1.005–1.031, *p* = 0.005), *Eubacterium* (*eligens group*) (OR: 1.016, 95% CI: 1.003–1.030, p = 0.021), and *Butyricimonas* (OR: 1.014, 95% CI: 1.006–1.023, *p* = 0.001).

Moreover, the protective effects of *Coprococcus 3* and *Butyricimonas* on daytime sleepiness were still significant after Bonferroni correction. However, after adjustment for body mass index, the effect of *Alloprevotella* (OR: 1.007, 95% CI: 1.000–1.014, *p* = 0.051) on daytime sleepiness was not nominally significant.

#### 3.1.3. Insomnia

Genetically predicted relative abundance of *Odoribacter* (OR: 0.976, 95% CI: 0.954–1.000, *p* = 0.044) and *Oscillibacter* (OR: 0.985, 95% CI: 0.974–0.996, *p* = 0.005) decreased the risk of insomnia. After Bonferroni correction, the effect of *Oscillibacter* on insomnia risk remained. By contrast, five bacterial taxa increased the risk of insomnia, namely, *Rikenellaceae RC9 gut group* (OR: 1.007, 95% CI: 1.000–1.015, *p* = 0.046), *Prevotella 7* (OR: 1.009, 95% CI: 1.002–1.017, *p* = 0.017), *Marvinbryantia* (OR: 1.014, 95% CI: 1.000–1.029, *p* = 0.049), *Clostridium* (*innocuum group*) (OR: 1.018, 95% CI: 1.005–1.031, *p* = 0.006), and *Lachnoclostridium* (OR: 1.029, 95% CI: 1.007–1.052, *p* = 0.009).

#### 3.1.4. Sleep duration

*Alistipes* (OR: 0.967, 95% CI: 0.938–0.997, *p* = 0.032) and *Eubacterium* (*hallii group*) (OR: 0.977, 95% CI: 0.959–0.997, *p* = 0.022) showed a negative correlation with sleep duration. By contrast, five intestinal flora genera were positively correlated with sleep duration according to the IVW estimates, namely, *Victivallis* (OR: 1.016, 95% CI: 1.003–1.028, *p* = 0.013), *Anaerofilum* (OR: 1.018, 95% CI: 1.002–1.035, *p* = 0.032), *Oscillibacter* (OR: 1.024, 95% CI: 1.005–1.043, *p* = 0.012), *Lachnospiraceae* UCG-004 (OR: 1.033, 95% CI: 1.004–1.064, *p* = 0.026), and *Odoribacter* (OR: 1.038, 95% CI: 1.001–1.077, *p* = 0.043).

#### 3.1.5. Long sleep duration

The IVW estimates suggested that the genetic forecast of abundance ratio of *Alistipes* (OR: 0.988, 95% CI: 0.980–0.997, *p* = 0.011), *Ruminococcaceae* NK4A214 group (OR: 0.990, 95% CI: 0.983–0.998, *p* = 0.009), and *Butyricimonas* (OR: 0.994, 95% CI: 0.988–1.000, *p* = 0.050) showed a negative correlation with long sleep duration. However, *Slackia* (OR: 1.007, 95% CI: 1.000–1.014, *p* = 0.037) and *Ruminiclostridium 6* (OR: 1.009, 95% CI: 1.001–1.016, *p* = 0.020) were positively correlated with long sleep duration.

#### 3.1.6. Short sleep duration

*Coprococcus* 1 (OR: 0.987, 95% CI: 0.975–1.000, *p* = 0.043), *Lachnospiraceae* UCG-004 (OR: 0.987, 95% CI: 0.978–0.997, *p* = 0.013), *Oscillibacter* (OR: 0.990, 95% CI: 0.981–0.998, *p* = 0.017), *Anaerofilum* (OR: 0.991, 95% CI: 0.985–0.997, *p* = 0.003), and *Eubacterium* (*fissicatena group*) (OR: 0.994, 95% CI: 0.988–1.000, *p* = 0.041) showed a negative correlation with short sleep duration. Three genera were positively correlated with short sleep duration, namely, *Eubacterium* (*hallii group*) (OR: 1.010, 95% CI: 1.002–1.018, *p* = 0.020), *Barnesiella* (OR: 1.010, 95% CI: 1.001–1.019, *p* = 0.030), and *Collinsella* (OR: 1.014, 95% CI: 1.001–1.027, *p* = 0.037). Furthermore, the causal link between *Anaerofilum* and short sleep duration was still significant after Bonferroni correction.

#### 3.1.7. Chronotype

The IVW method yielded nominal associations of four intestinal flora genera with chronotype, namely, *Bacteroides* (OR: 0.955, 95% CI: 0.918–0.993, *p* = 0.019), *Parabacteroides* (OR: 0.952, 95% CI: 0.916–0.989, *p* = 0.011), *Eubacterium* (*coprostanoligenes group*) (OR: 0.961, 95% CI: 0.927–0.998, *p* = 0.036), and *Prevotella 7* (OR: 0.982, 95% CI: 0.968–0.997, *p* = 0.015). Genetically predicted relative abundance of *Intestinibacter* (OR: 1.026, 95% CI: 1.003–1.049, *p* = 0.025) and *Tyzzerella 3* (OR: 1.030, 95% CI: 1.009–1.052, *p* = 0.006) had positive causal contributions to chronotype.

After using the binary phenotype of the chronotype, the significant difference of all these associations persisted except for *Eubacterium* (*coprostanoligenes group*) (OR: 0.987, 95% CI: 0.974–1.000, *p* = 0.065). Furthermore, nominal significant effects on chronotype were observed for *Bifidobacterium* (OR: 0.987, 95% CI: 0.977–0.996, *p* = 0.006), *Ruminococcus 1* (OR: 0.986, 95% CI: 0.975–0.998, *p* = 0.019), *Catenibacterium* (OR: 1.009, 95% CI: 1.001–1.017, *p* = 0.025), *Victivallis* (OR: 1.006, 95% CI: 1.000–1.012, *p* = 0.032), and *Alloprevotella* (OR: 0.992, 95% CI: 0.985–1.000, *p* = 0.037). In addition, the influence of *Tyzzerella 3* on chronotype was more significant.

#### 3.1.8. Sensitivity analysis

For the forward MR analysis, *p*-values derived from Cochran’s Q were all >0.05, except for estimates of *Clostridium* (*innocuum group*) on insomnia, *Oxalobacter* on daytime napping and daytime sleepiness, *Lachnoclostridium* on insomnia, and *Tyzzerella 3* on chronotype, which showed that there was no significant heterogeneity. Except for *Eisenbergiella*, all *p*-values of MR-Egger intercept tests were > 0.05 (Supplementary Table S4), suggesting that no horizontal pleiotropy appeared in forward MR analysis. This was also confirmed by “leave-one-out” analysis and funnel plots (Supplementary Figures S1–S73).

### 3.2. Causal effects of sleep-related traits on gut microbiota

According to the selection criteria of the IVs, we selected 78 SNPs for daytime napping, 33 SNPs for daytime sleepiness, 34 SNPs for insomnia, 58 SNPs for sleep duration, four SNPs for long sleep duration, 23 SNPs for short sleep duration, and 117 SNPs for chronotype. Details about the selected SNPs are displayed in Supplementary Tables S5–S13. Except for IVs for long sleep duration and short sleep duration, the F-statistics for IVs were larger than 10.

**Figure 3:**
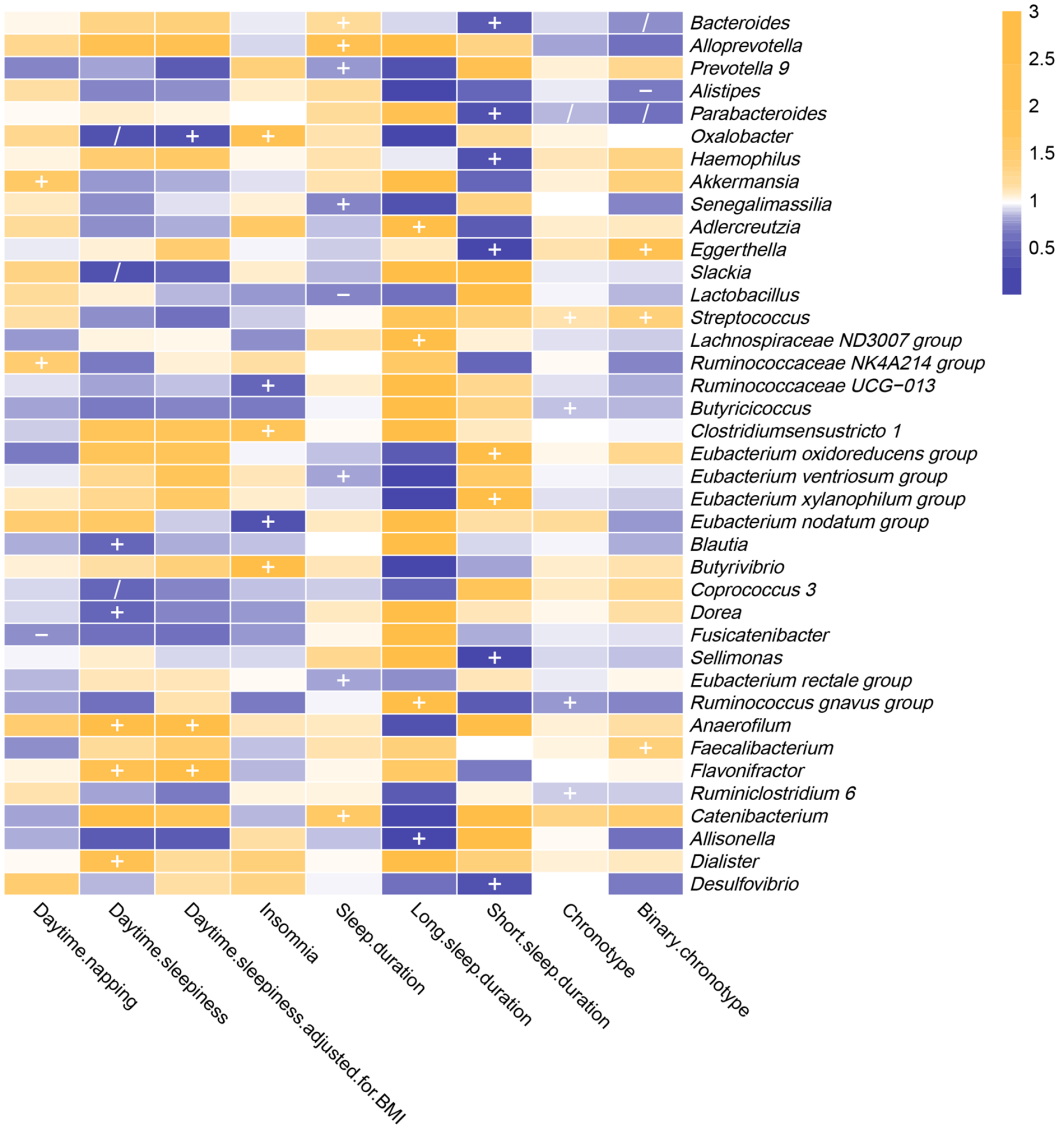
In the reverse MR analysis, IVW estimates from seven sleep-associated traits (daytime napping, daytime sleepiness, insomnia, sleep duration, long sleep duration, short sleep duration and chronotype) on 39 bacterial genera. The color of each block represents the OR of every MR analysis (blue, OR < 1; orange, OR > 1). *p*-values of <0.05 were marked with “+,” *p*-values <0.05 in both forward and reverse MR analyses were marked with “/” and *p*-values of <0.05 with potential pleiotropy were marked with “−”.

As shown in Figure 3 and Supplementary Table S14, the results of reverse MR analysis indicated that daytime napping was correlated with three bacterial taxa, daytime sleepiness was correlated with eight bacterial taxa, insomnia was correlated with five bacterial taxa, sleep duration was correlated with eight bacterial taxa, long sleep duration was correlated with four bacterial taxa, short sleep duration was correlated with eight bacterial taxa, and chronotype was correlated with five bacterial taxa.

The IVW estimates indicated that daytime napping had causal contribution to the reduction of *Fusicatenibacter* abundance (OR: 0.739, 95% CI: 0.554–0.987, *p* = 0.040), while being positively correlated with *Akkermansia* (OR: 1.530, 95% CI: 1.081–2.166, *p* = 0.017) and *Ruminococcaceae NK4A214 group* (OR: 1.527, 95% CI: 1.115–2.092, *p* = 0.008).

The IVW estimates indicated that daytime sleepiness was negatively correlated with five bacterial taxa, namely, *Oxalobacter* (OR: 0.302, 95% CI: 0.101–0.904, *p* = 0.032), *Slackia* (OR: 0.339, 95% CI: 0.135–0.854, *p* = 0.022), *Coprococcus 3* (OR: 0.541, 95% CI: 0.304–0.962, *p* = 0.037), *Blautia* (OR: 0.555, 95% CI: 0.324–0.950, *p* = 0.032), and *Dorea* (OR: 0.556, 95% CI: 0.322–0.961, *p* = 0.035). In addition, there were suggestive associations between daytime sleepiness with three bacterial taxa, namely, *Dialister* (OR: 2.122, 95% CI: 1.093–4.117, *p* = 0.026), *Flavonifractor* (OR: 2.292, 95% CI: 1.140–4.608, *p* = 0.020), and *Anaerofilum* (OR: 3.688, 95% CI: 1.246–10.916, *p* = 0.018). However, after additional adjustment for body mass index, the effects of daytime sleepiness on *Slackia*, *Dialister*, *Blautia*, *Dorea*, and *Coprococcus 3* were not significant.

Insomnia was negatively correlated with *Eubacterium* (*nodatum group*) (OR: 0.310, 95% CI: 0.100–0.961, *p* = 0.043) and *Ruminococcaceae UCG-013* (OR: 0.522, 95% CI: 0.345–0.791, *p* = 0.002), while being positively correlated with three bacterial taxa, namely, *Clostridium sensu stricto 1* (OR: 1.708, 95% CI: 1.085–2.687, *p* = 0.021), *Oxalobacter* (OR: 2.434, 95% CI: 1.104–5.369, *p* = 0.027), and *Butyrivibrio* (OR: 2.656, 95% CI: 1.005–7.016, *p* = 0.049).

The results of the IVW method revealed that sleep duration was negatively correlated with five intestinal flora genera, namely, *Senegalimassilia* (OR: 0.709, 95% CI: 0.507–0.991, *p* = 0.044), *Lactobacillus* (OR: 0.726, 95% CI: 0.534–0.988, *p* = 0.041), *Prevotella 9* (OR: 0.776, 95% CI: 0.604–0.998, *p* = 0.048), *Eubacterium* (*ventriosum group*) (OR: 0.786, 95% CI: 0.641–0.964, *p* = 0.021) and *Eubacterium* (*rectale group*) (OR: 0.794, 95% CI: 0.655–0.962, *p* = 0.018). It was positively correlated with three bacterial taxa, namely, *Bacteroides* (OR: 1.225, 95% CI: 1.016–1.477, *p* = 0.033), *Catenibacterium* (OR: 1.649, 95% CI: 1.017–2.672, *p* = 0.042), and *Alloprevotella* (OR: 1.946, 95% CI: 1.104–3.427, *p* = 0.021).

The F-statistic of SNPs for long sleep duration and short sleep duration was less than 10. Therefore, weak instrumental bias could disturb the conclusions of reverse MR analysis (Supplementary Table S15).

Chronotype was negatively correlated with the genetic forecast of abundance ratio of four bacterial taxa, specifically, *Ruminococcus* (*gnavus group*) (OR: 0.781, 95% CI: 0.654–0.933, *p* = 0.006), *Parabacteroides* (OR: 0.858, 95% CI: 0.768–0.957, *p* = 0.006), *Butyricicoccus* (OR: 0.870, 95% CI: 0.782–0.969, *p* = 0.011), and *Ruminiclostridium 6* (OR: 0.884, 95% CI: 0.783–0.998, *p* = 0.047). It was positively correlated with *Streptococcus* (OR: 1.129, 95% CI: 1.011–1.260, *p* = 0.031). However, after using the binary phenotype of the chronotype, the associations of chronotype with *Ruminococcus* (*gnavus group*), *Butyricicoccus*, and *Ruminiclostridium 6* became insignificant. In addition, significant effects of chronotype on *Eggerthella* (OR: 2.124, 95% CI: 1.145–3.941, *p* = 0.017), *Alistipes* (OR: 0.689, 95% CI: 0.489–0.972, *p* = 0.034), *Bacteroides* (OR: 0.736, 95% CI: 0.543–0.997, *p* = 0.048), and *Faecalibacterium* (OR: 1.399, 95% CI: 1.001–1.954, *p* = 0.049) were observed.

For the reverse MR analysis, *p*-values derived from Cochran’s Q were all >0.05 (Supplementary Table S15). In other words, there was no evidence of significant heterogeneity. Except for *Fusicatenibacter*, *Lactobacillus*, and *Alistipes*, all *p*-values of MR-Egger intercept tests were > 0.05, suggesting that no horizontal pleiotropy appeared. Furthermore, through funnel plots and “leave-one-out” analysis, we found no causal associations between sleep-related traits and bacterial genera that were primarily driven by any single SNP (Supplementary Figures S74–S123).

## 4. Discussion

**Figure 4:**
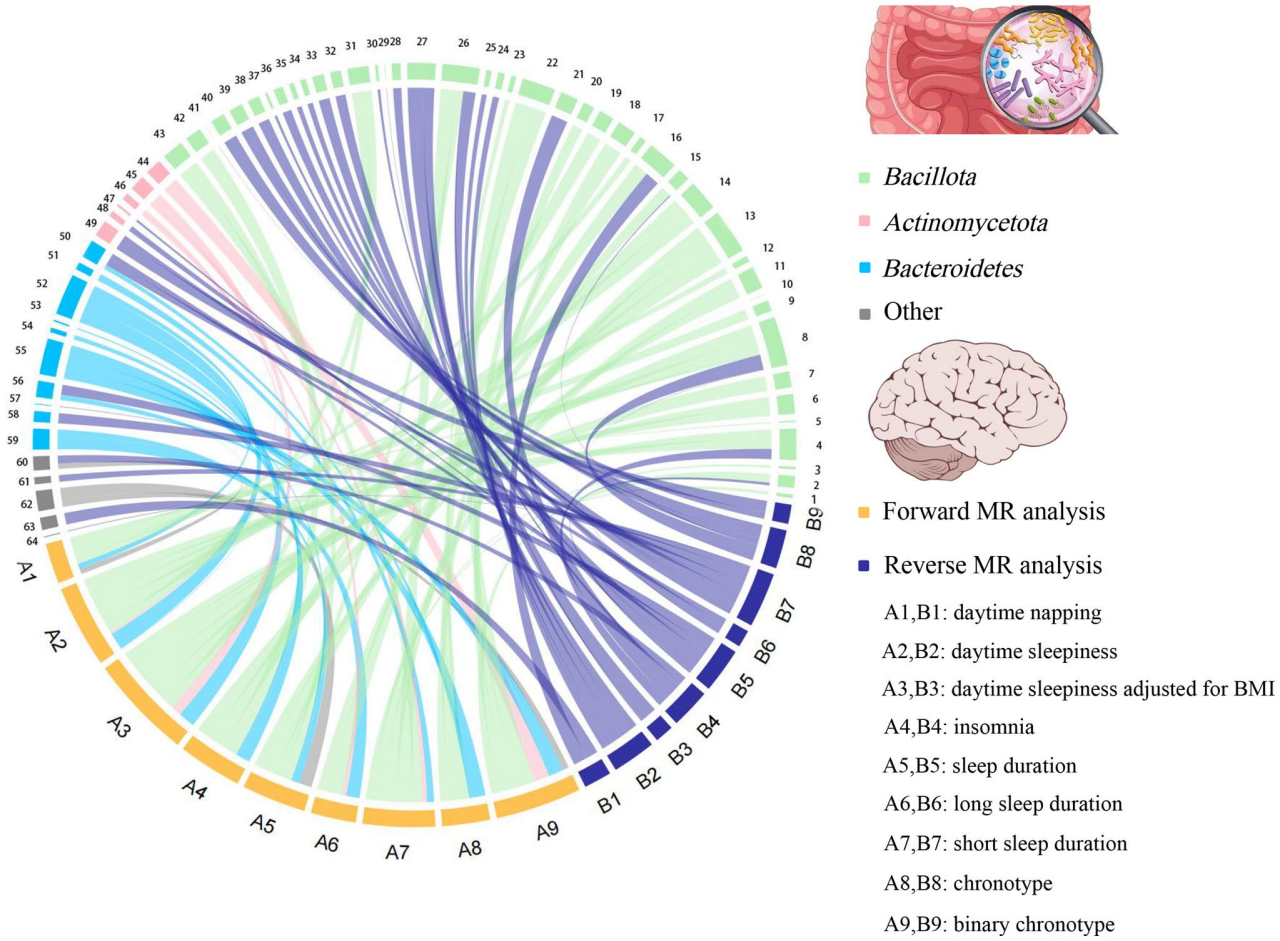
The chord plot showed the causal relationships between gut microbiota and sleep. 1: *Eubacterium fissicatena* group; 2: Catenibacterium; 3: Coprococcus 1; 4: Clostridium *sensu stricto* 1; 5: Defluviitaleaceae UCG011; 6: Coprococcus 2; 7: Coprococcus 3; 8: *Ruminococcus gnavus* group; 9: Holdemanella; 10: Marvinbryantia; 11: Lachnospiraceae UCG004; 12: Lachnospiraceae UCG010; 13: Oscillibacter; 14: Peptococcus; 15: Ruminiclostridium 6; 16: Ruminococcaceae NK4A214 group; 17: Ruminococcaceae UCG002; 18: Intestinibacter; 19: Lachnoclostridium; 20: Ruminococcus 1; 21: Ruminococcaceae UCG013; 22: Tyzzerella 3; 23: *Eubacterium coprostanoligenes* group; 24: Blautia; 25: Dorea; 26: Anaerofilum; 27: Flavonifractor; 28: Dialister; 29: Butyrivibrio; 30: *Eubacterium nodatum* group; 31: *Eubacterium eligens* group; 32: *Eubacterium ventriosum* group; 33: *Eubacterium rectale* group; 34: Lachnospiraceae ND3007 group; 35: Allisonella; 36: *Eubacterium oxidoreducens* group; 37: *Eubacterium xylanophilum* group; 38: Sellimonas; 39: Streptococcus; 40: Butyricicoccus; 41: Faecalibacterium; 42: *Clostridium innocuum* group; 43: *Eubacterium hallii* group; 44: Bifidobacterium; 45: Collinsella; 46: Slackia; 47: Senegalimassilia; 48: Adlercreutzia; 49: Eggerthella; 50: Bacteroides; 51: Barnesiella; 52: Butyricimonas; 53: Rikenellaceae RC9 gut group; 54: Odoribacter; 55: Prevotella7; 56: Alloprevotella; 57: Prevotella9; 58: Parabacteroides; 59: Alistipes; 60: Oxalobacter; 61: Haemophilus; 62: Victivallis; 63: Akkermansia; 64: Desulfovibrio.

To the best of our knowledge, this is the first bidirectional MR analysis that comprehensively clarifies causal relationships between gut microbiota and sleep-related traits. As shown in Figures 2, 3, our findings indicate that a total of 68 bacterial taxa are causally associated with seven sleep-related traits. Furthermore, 13 bacterial taxa related to sleep-related features in forward MR analysis were regulated by sleep-related traits, including *Alistipes*, *Alloprevotella*, *Anaerofilum*, *Bacteroides*, *Catenibacterium*, *Coprococcus 3*, *Oxalobacter*, *Parabacteroides*, *Ruminiclostridium 6*, *Ruminococcaceae NK4A214 group*, *Ruminococcaceae UCG-013*, *Ruminococcus gnavus group*, and *Slackia*. Nevertheless, the potential causal effects of *Coprococcus 3*, *Oxalobacter*, and *Slackia* on daytime sleepiness, and the potential causal effect of *Parabacteroides* and *Bacteroides* on chronotype in forward MR analysis were not supported by the results of reverse MR analysis. However, these findings did not exclude the possibility that the effects are interactive. Moreover, owing to potential pleiotropy, some causal effects (*Eisenbergiella* on daytime napping, daytime napping on *Fusicatenibacter*, sleep duration on *Lactobacillus*, and binary chronotype on *Alistipes*) were not credible. After excluding the above uncertain causal effects, a total of 40 bacterial taxa had potential causal effects on seven sleep-related traits, which in the other direction may be related to 34 bacterial taxa, and most of the bacterial taxa were of *Bacillota* (Figure 4).

**Figure 5:**
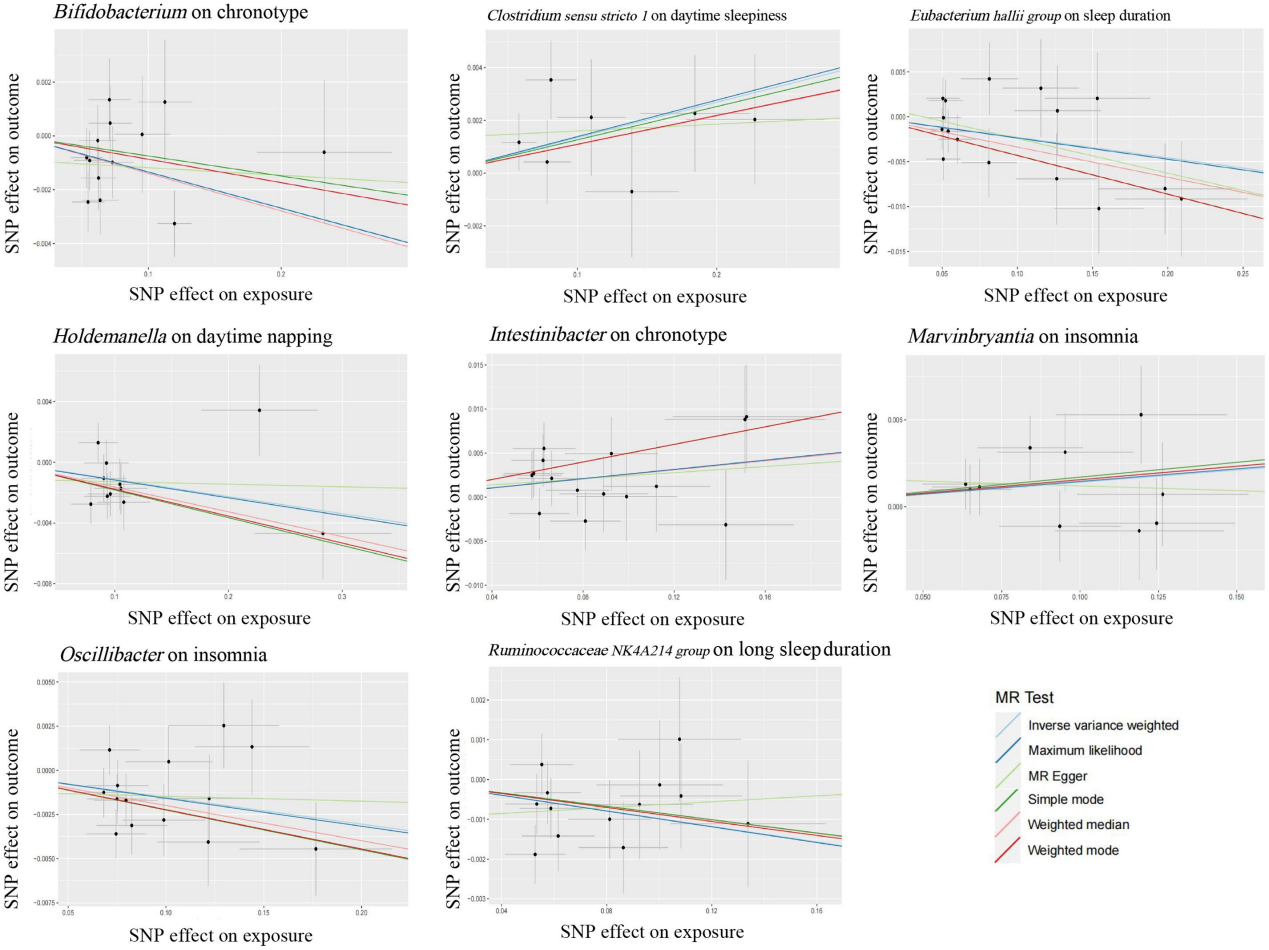
Scatter plots for the causal association between several bacteria associated with butyrate-producing and sleep.

**Figure 6:**
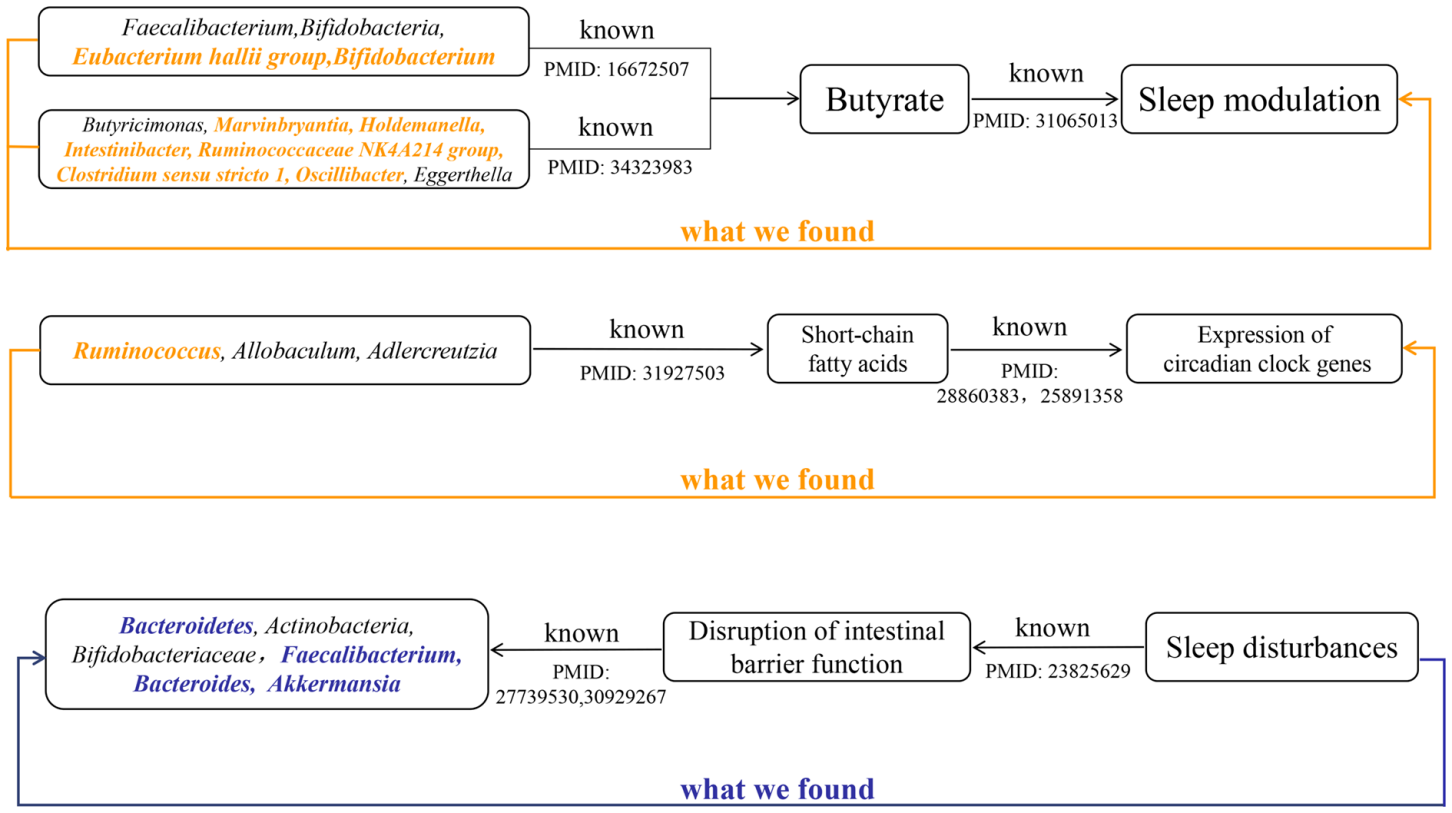
The summary of the findings in our study and the existing knowledge in literature about different mechanisms that affect the sleep. Black indicated known evidence, bold orange italics indicated the findings by forward MR analysis, and bold blue italics indicated the findings by reverse MR analysis.

Growing evidence from observational studies indicates that gut microbiota is correlated with sleep-related traits and disorders, and that the absence of gut microbes and their metabolites may alter sleep traits and architecture (Leone et al., 2015; Paulsen et al., 2017; Szentirmai et al., 2019). Szentirmai et al. found that the intestinal microbiome induces non-REM sleep through butyrate-sensitive mechanisms (Szentirmai et al., 2019). Cross-feeding is the central metabolic mechanism of the gut microbiota. *Faecalibacterium* can produce butyrate from acetate and lactate, which are produced by *Bifidobacteria* from fermented carbohydrates. Moreover, the coculture of *Eubacterium hallii group* with *Bifidobacterium* promotes the accumulation of butyrate through cross-feeding (Belenguer et al., 2006). *Butyricimonas*, *Marvinbryantia*, *Holdemanella*, *Intestinibacter*, *Ruminococcaceae NK4A214 group*, *Clostridium sensu stricto 1*, and *Oscillibacter* have also been associated with the production of butyrate, while *Eggerthella* is involved in the depletion of butyrate (Chen et al., 2021; Liu et al., 2022). Consistent with these previous studies, our study found that these butyrate-producing bacteria (*Bifidobacterium*, *Clostridium sensu stricto 1*, *Eubacterium hallii group*, *Holdemanella*, *Intestinibacter*, *Marvinbryantia*, *Oscillibacter*, and *Ruminococcaceae NK4A214 group*) all had a nominally significant causal association with sleep (Figure 5). This finding suggests that these intestinal microbiotas associated with butyrate metabolism are involved in sleep-related regulatory mechanisms (Figure 6). Lipopolysaccharides and peptidoglycans, which are components of bacterial cell walls, are released during the decomposition or division of bacteria and then produce an inflammatory response by activating the expression of proinflammatory factors (Motta et al., 2015; Krueger and Opp, 2016). Previous research has confirmed that these inflammatory responses are related to the sleep that occurs during bacterial infections. However, the butyrate produced by intestinal flora has a strong anti-inflammatory effect and can inhibit inflammation in the colon and liver as well as the expression of inflammatory factors induced by lipopolysaccharides and NF-κB activation (Perez et al., 1998; Thangaraju et al., 2009; D'Souza et al., 2017).

Metabolites of gut microbes such as short-chain fatty acids, butyrate and, acetate may also regulate circadian rhythms by influencing the expression of circadian clock genes (Figure 6). Wang et al. have demonstrated that the gut microbiome modulates the expression of the circadian transcription factor NFIL 3 (Wang et al., 2017). Furthermore, the absence of gut microbiota and gut microbial metabolites such as butyrate and acetic can led to significant differential expression of hepatic and central circadian clock genes regardless of dietary changes (Leone et al., 2015). An observational study determined that short-chain fatty acids, propionate, and butyrate in feces were associated with nighttime sleep duration in infants (Heath et al., 2020). Another observational study also found that changes in abundance of *Lachnospira*, *Bacteroides*, *Faecalibacterium*, and *Blautia* were significantly associated with sleep quality and disorders (Li et al., 2020), which correspond to our findings. Yu et al. found that after giving mice oral high doses of gamma-aminobutyric acid fermented milk, the relative abundance of *Ruminococcus*, *Allobaculum*, and *Adlercreutzia*, and the levels of short-chain fatty acids increased significantly and sleep time was significantly prolonged (Yu et al., 2020). This result suggests that diet may affect sleep by regulating the intestinal microbiota. These previous studies and our research show that the gut microbiota and its metabolites can participate in the regulation of sleep.

The relative abundance of gut microbiota is unique between individuals, and under healthy conditions, the gut microbiota displays resilience and stability. However, the “healthy” microbiome can be disrupted by changes in age, disease and environmental factors (Hou et al., 2022). Sleep disturbances or circadian rhythm disturbances have also been reported to disrupt the balance of the gut microbiota. Circadian rhythms are essential for maintaining normal physiological functions of the gastrointestinal tract, and circadian rhythm disorders are closely related to certain diseases of the digestive system (Schernhammer et al., 2003; Hoogerwerf et al., 2007; Nojkov et al., 2010). *In vitro* experiments by Summa et al. proved that circadian rhythm disturbance and sleep fragmentation lead to the destruction of the integrity of intestinal barrier function, which in turn increased intestinal permeability (Summa et al., 2013). This increased intestinal permeability may lead to translocations of gut microbiota and its metabolites, which alters the variety and abundance of gut microbiota. Poroyko et al. also demonstrated through *in vitro* experiments that sleep fragmentation can induce selective changes in intestinal flora, such as reducing the abundance of *Bacteroidetes*, *Actinobacteria*, and *Bifidobacteriaceae* (Poroyko et al., 2016). However, the disruption of the integrity of the intestinal barrier caused by sleep disorder may be related to its suppression of melatonin levels (Gao et al., 2019). Gao et al. found that the decrease in the abundance of *Faecalibacterium*, *Bacteroides*, and *Akkermansia* caused by sleep deprivation was associated with decreased levels of melatonin (Gao et al., 2019). Consistent with these conclusions, we also found nominally causal effects of sleep disorder or sleep fragmentation on these bacterial taxa (Figure 6).

The previous studies mentioned above demonstrate that gut microbiota are involved in circadian rhythm regulation and show that circadian rhythm disturbances may cause changes in gut microbiota abundance by damaging the intestinal barrier or inducing changes in melatonin. This is consistent with the results from 13 bacterial taxa found in our study that were both involved in and subject to sleep regulation, which suggests that regulation between gut microbiota and sleep may be bidirectional.

A major advantage of this study was that MR analysis effectively excluded the interference of reverse causation and possible confounding factors in inferring causal effects between gut microbiota and sleep-related traits. The SNPs of the intestinal microbiota came from the largest GWAS meta-analysis available, and the sample sizes were large enough to ensure the strength of the IVs and the robustness of the results. The design of the two-sample MR further avoided bias resulting from overlapping data on exposure and outcome pools. Utilization of various statistical models (such as IVW, weighted median, and maximum likelihood method) as well as sensitivity analyses (such as MR-PRESSO and MR-Egger regression intercept term tests) ensured the confidence of causal effect estimates.

However, our study also had some limitations. First, genus level was the lowest classification level in the data for gut microbiota, which limited the ability to uncover causal relationships between gut microbes and sleep at the species level. Second, the SNPs we utilized in the forward MR analysis did not meet the conventional GWAS threshold (*p* < 5 × 10^−8^), but were rescued by Bonferroni correction, which ruled out false positive results to the greatest extent. Third, the participants of gut microbiota and sleep in the GWAS meta-analysis were primarily of European ancestry. The same genetic variant may have different pleiotropic effects in different ethnic populations; therefore, the inference of causal effects derived in our study may not be applicable in non-European populations. Fourth, in the reverse MR analysis, estimates of effects may have been biased by weak IVs because of the small sample size of the GWAS meta-analysis for sleep-related traits. Finally, we applied a number of exclusion criteria to select IVs; however, many internal and external factors affect gut microbes and sleep. Thus, bias resulting from SNPs being associated with potential risk factors cannot be completely ruled out.

In conclusion, this study represents the first bidirectional MR analysis to systematically reveal the causal association between gut microbiota and sleep. Our findings suggest the possible causal effect of 42 bacterial genera on sleep-related traits. Conversely, sleep-related traits may also be involved in the regulation of the abundance of 39 bacterial genera. In addition, 13 of these bacterial genera overlapped, which provides suggestive evidence for a reciprocal role between gut microbiota and sleep. The demonstration of a causal relationship between sleep and gut microbiota provides support for techniques to modify sleep by manipulating the gut microbiome. However, the basic mechanism of gut microbiota on sleep is still unknown, and more research is needed to provide theoretical support for targeted intervention in sleep by regulating specific gut microbiota.

## Data availability statement

Publicly available datasets were analyzed in this study. This data can be found at: Summary statistics for the sleep traits are available at: (http://sleepdisordergenetics.org/). Summary statistics for the gut microbiota are available at: (https://mibiogen.gcc.rug.nl/).

## Author contributions

DO and JW designed the research. JW, BZ, and SZ collected and analyzed the data and drafted the manuscript. DO, XZ, and XL supervised the study. JW, ZH, and YX were involved in writing the manuscript. All authors contributed to the article and approved the submitted version.

## Funding

This work was supported by the National Natural Science Foundation of China (NO. 82271336) and Taizhou “500 Elite Plan” high-level talent project.

## Conflict of interest

The authors declare that the research was conducted in the absence of any commercial or financial relationships that could be construed as a potential conflict of interest.

## Publisher’s note

All claims expressed in this article are solely those of the authors and do not necessarily represent those of their affiliated organizations, or those of the publisher, the editors and the reviewers. Any product that may be evaluated in this article, or claim that may be made by its manufacturer, is not guaranteed or endorsed by the publisher.

## Abbreviations

MR: Mendelian randomization
OR: Odds ratio
CI: Confidence interval
REM: Rapid eye movement
RCT: Randomized controlled trials
SNP: Single-nucleotide polymorphism
GWAS: Genome-wide association study
MR-PRESS: MR pleiotropy residual sum and outlier
IVW: Inverse-variance weighted
IV: Instrumental variables
