# Supplementary material for: Associations between gut microbiota and sleep: a two-sample, bidirectional Mendelian randomization study
Source: Front Microbiol. 2023 Aug 14;14:1236847. doi: 10.3389/fmicb.2023.1236847 (PMC10461450; doi:10.3389/fmicb.2023.1236847)
Supplement: Supplementary file 1 [file Data_Sheet_1.docx]

**Supplemental Figure**

**Figure S1 Scatter plot (A), forest plot (B), funnel plot (C) and sensitivity analysis (D) of the causal effect of *Eubacterium fissicatena group* on short sleep duration.**

**
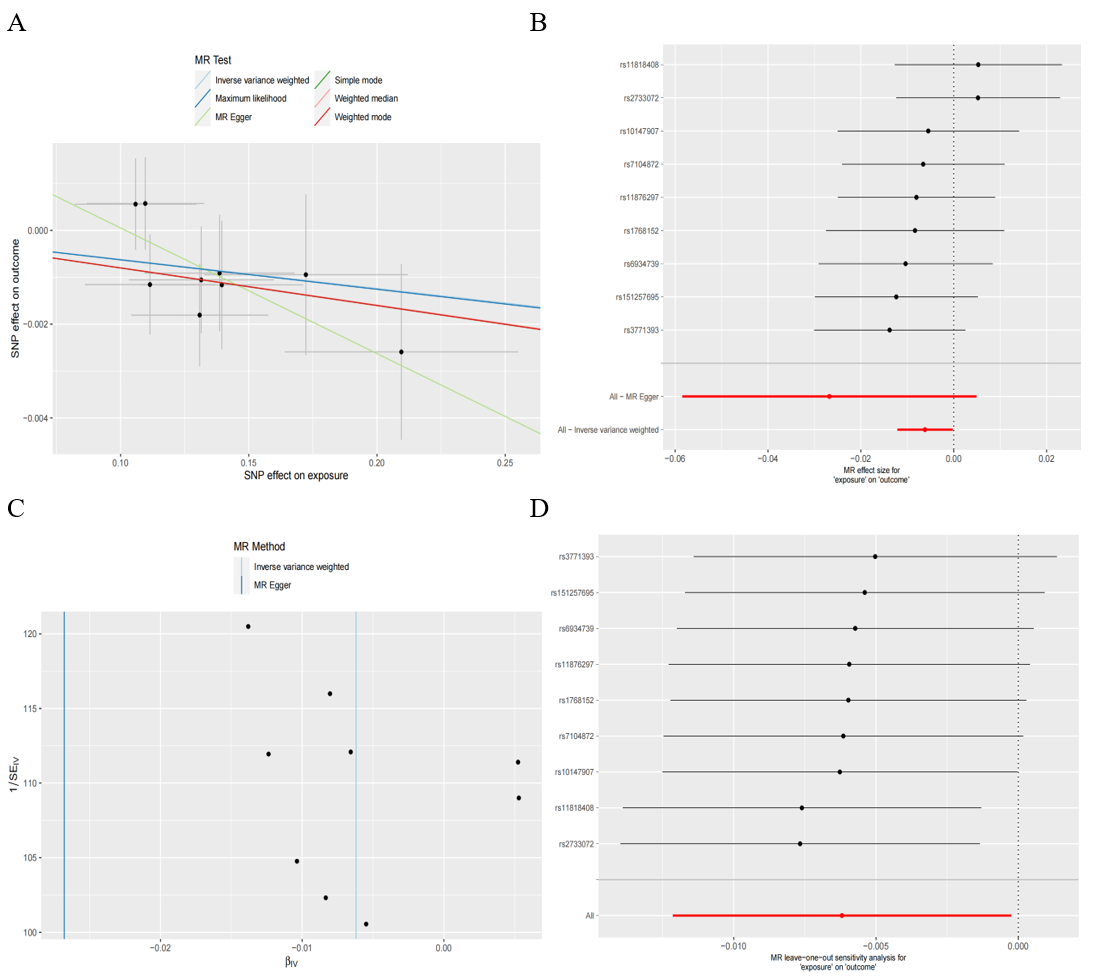
**

**Figure S2 Scatter plot (A), forest plot (B), funnel plot (C) and sensitivity analysis (D) of the causal effect of *Ruminococcus gnavus group* on daytime napping.**

**
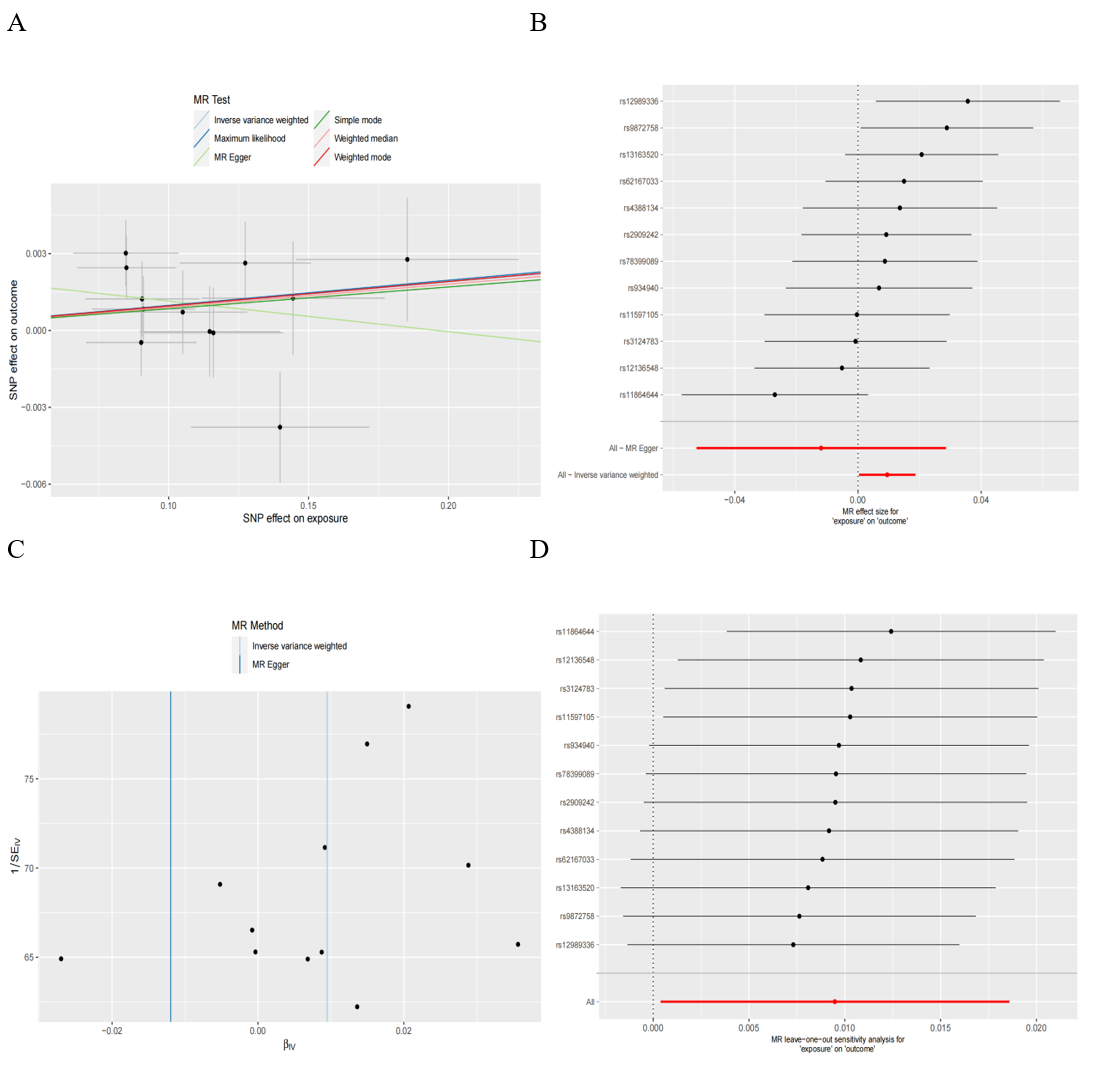
**

**Figure S3 Scatter plot (A), forest plot (B), funnel plot (C) and sensitivity analysis (D) of the causal effect of *Ruminococcus gnavus group* on daytime sleepiness.**

**
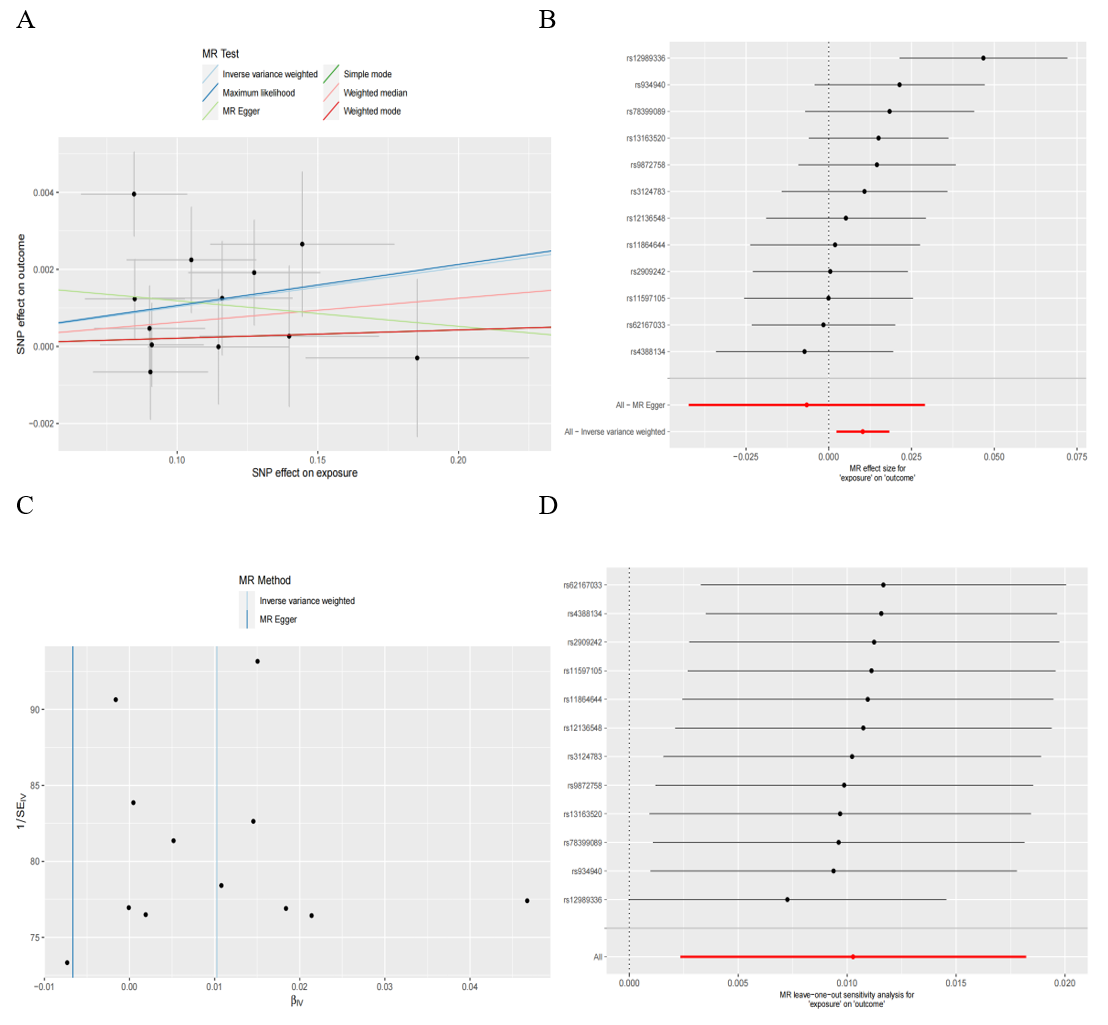
**

**Figure S4 Scatter plot (A), forest plot (B), funnel plot (C) and sensitivity analysis (D) of the causal effect of *Ruminococcus gnavus group* on daytime sleepiness adjusted for BMI.**

**
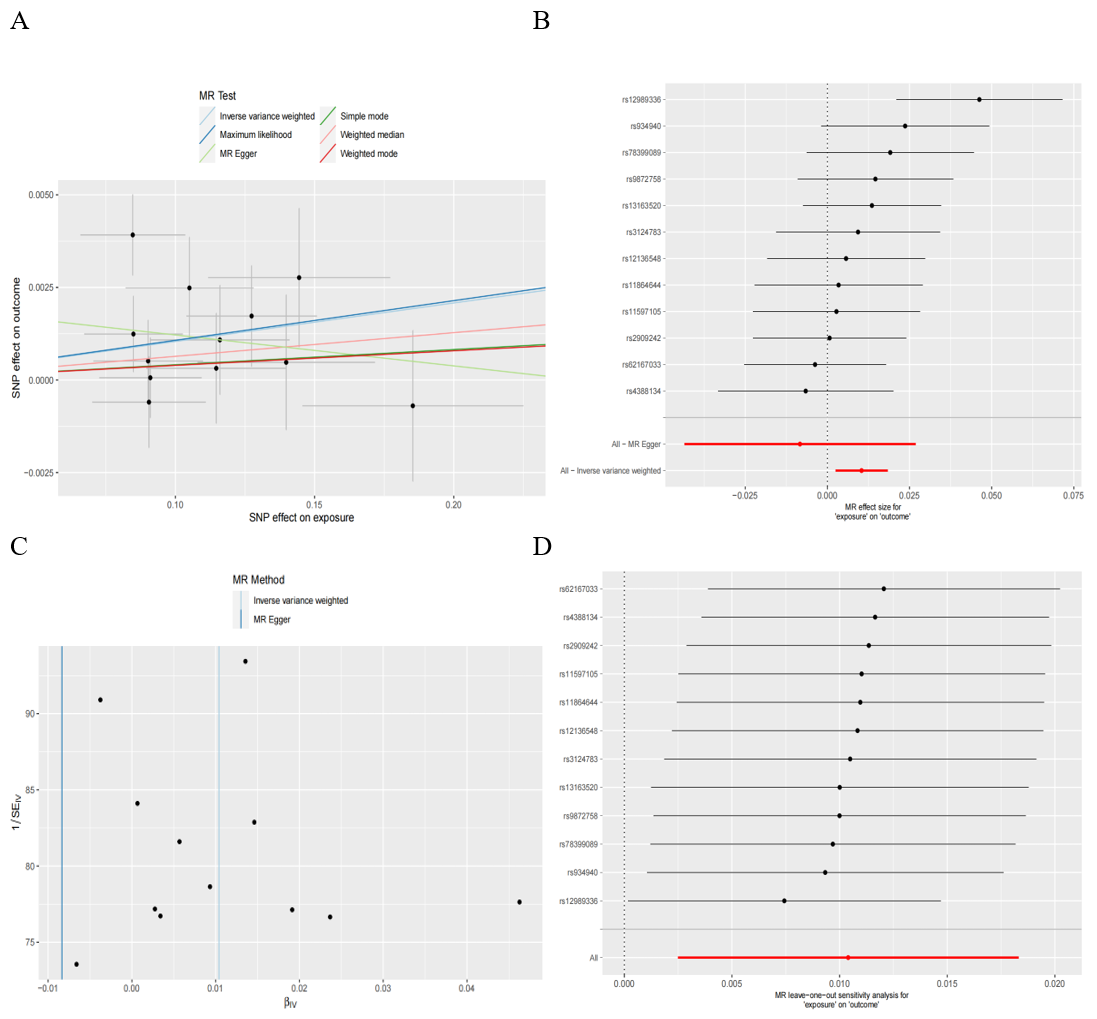
**

**Figure S5 Scatter plot (A), forest plot (B), funnel plot (C) and sensitivity analysis (D) of the causal effect of *Alloprevotella* on daytime sleepiness.**

**
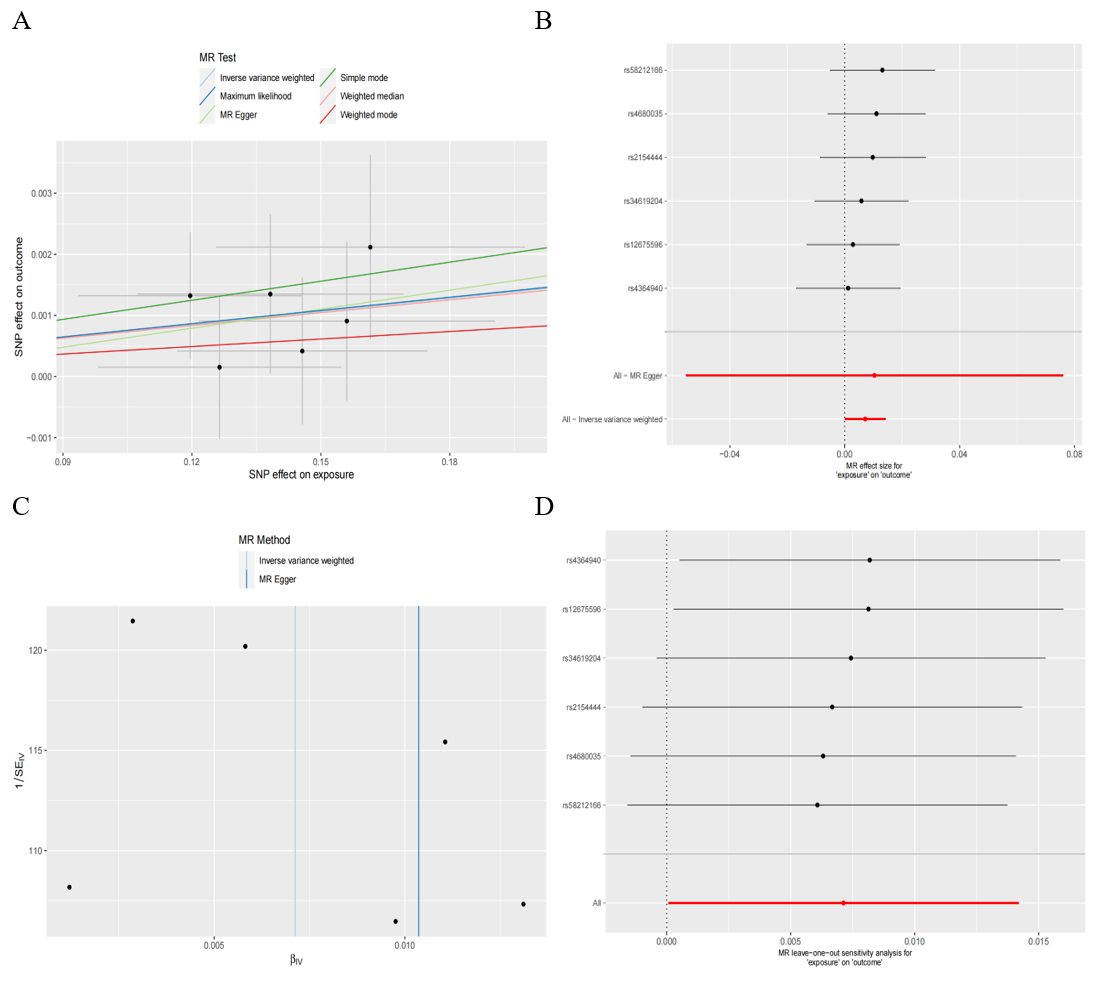
**

**Figure S6 Scatter plot (A), forest plot (B), funnel plot (C) and sensitivity analysis (D) of the causal effect of *Alloprevotella* on binary chronotype.**

**
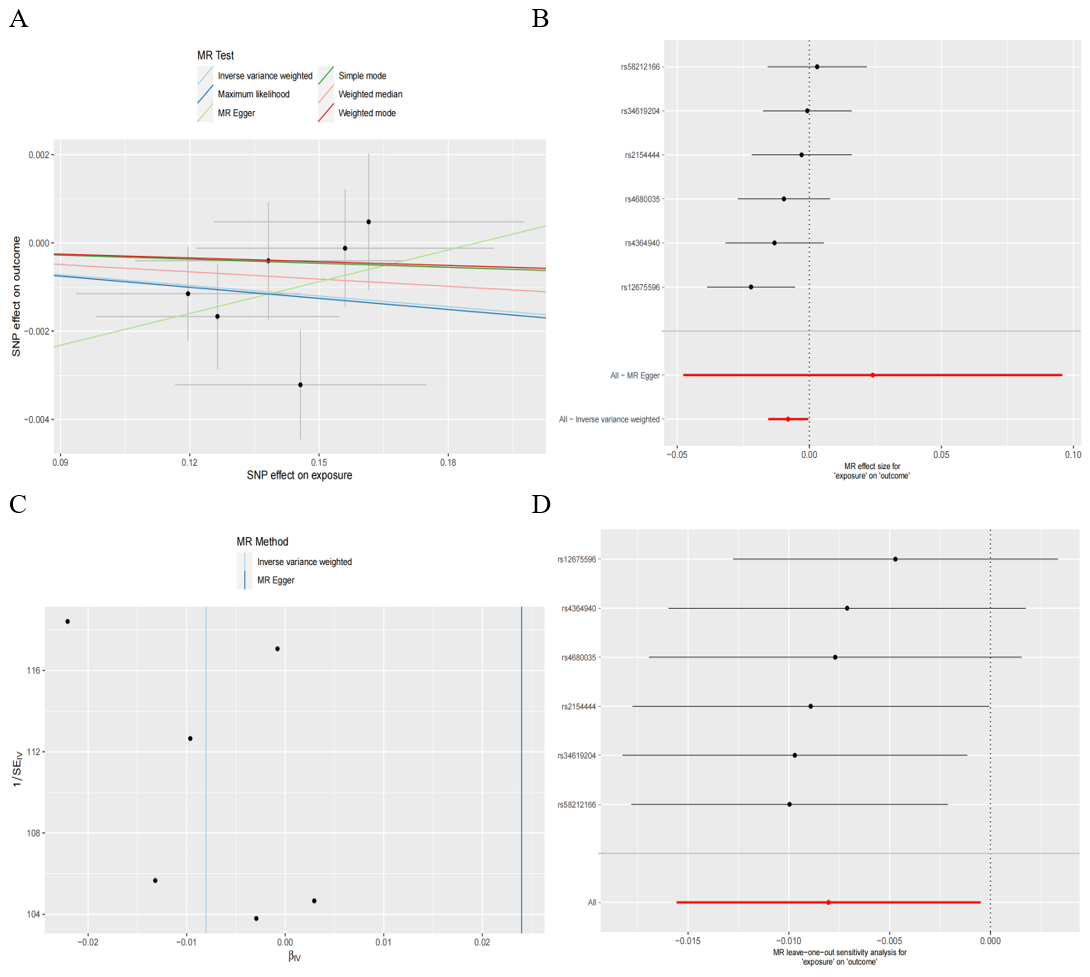
**

**Figure S7 Scatter plot (A), forest plot (B), funnel plot (C) and sensitivity analysis (D) of the causal effect of *Eubacterium coprostanoligenes group* on chronotype.**

**
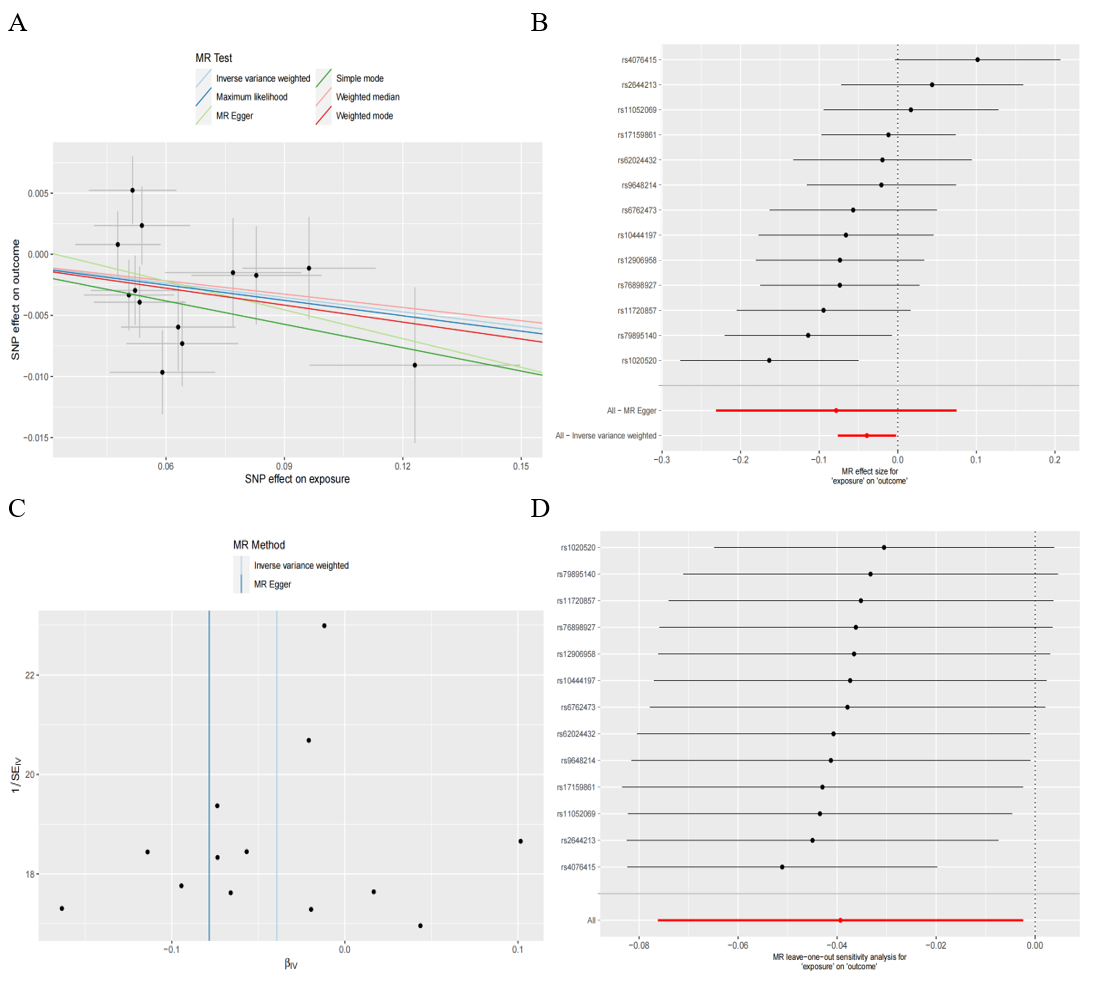
**

**Figure S8 Scatter plot (A), forest plot (B), funnel plot (C) and sensitivity analysis (D) of the causal effect of *Eubacterium eligens group* on daytime sleepiness.**

**
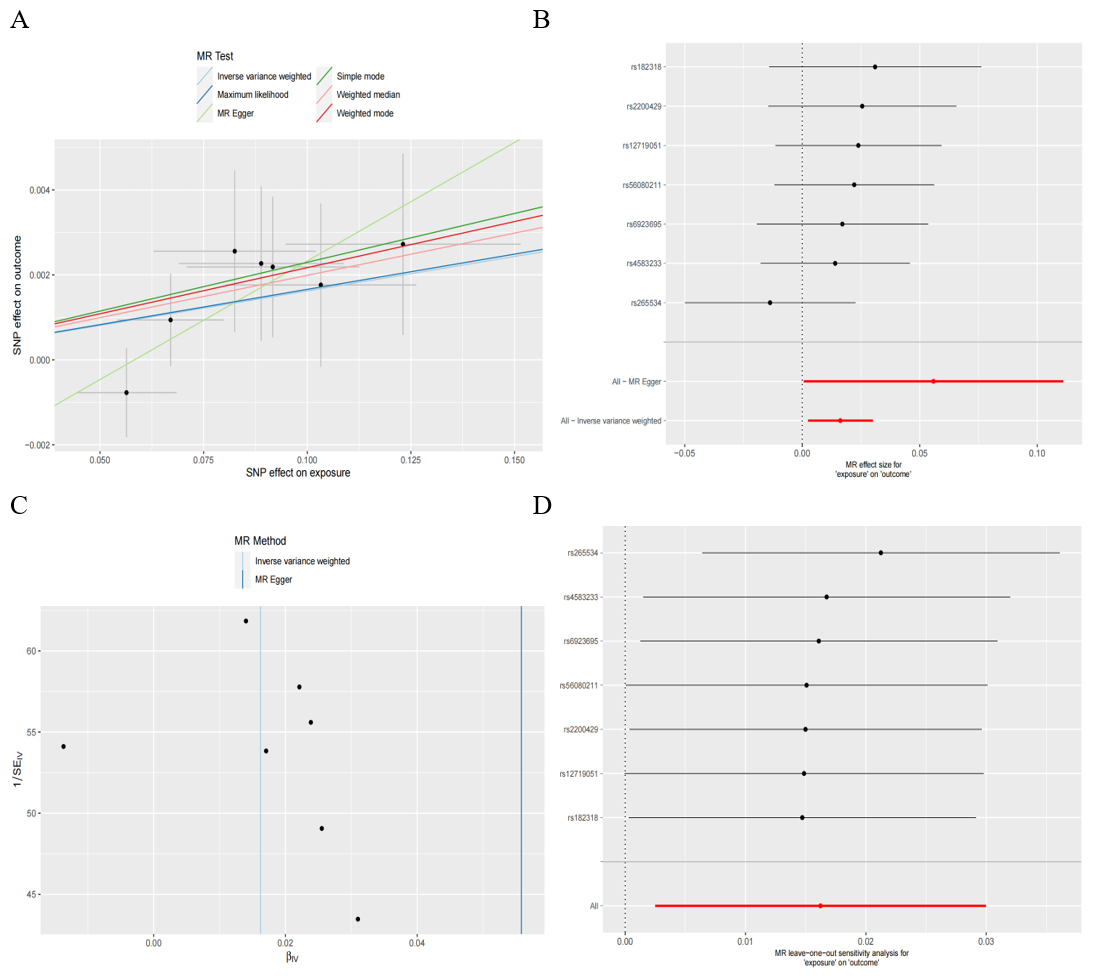
**

**Figure S9 Scatter plot (A), forest plot (B), funnel plot (C) and sensitivity analysis (D) of the causal effect of *Eubacterium eligens group* on daytime sleepiness adjusted for BMI.**

**
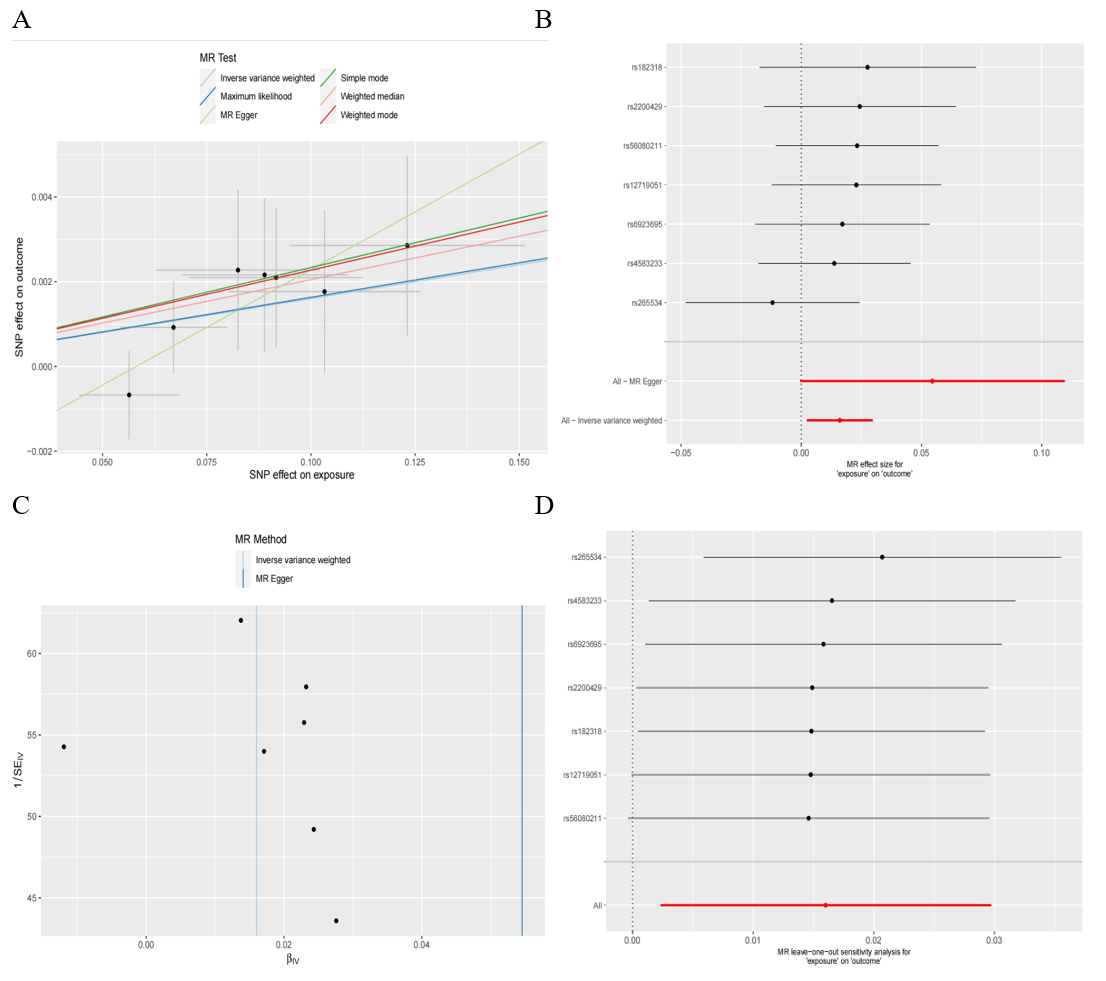
**

**Figure S10 Scatter plot (A), forest plot (B), funnel plot (C) and sensitivity analysis (D) of the causal effect of *Anaerofilum* on sleep duration.**

**
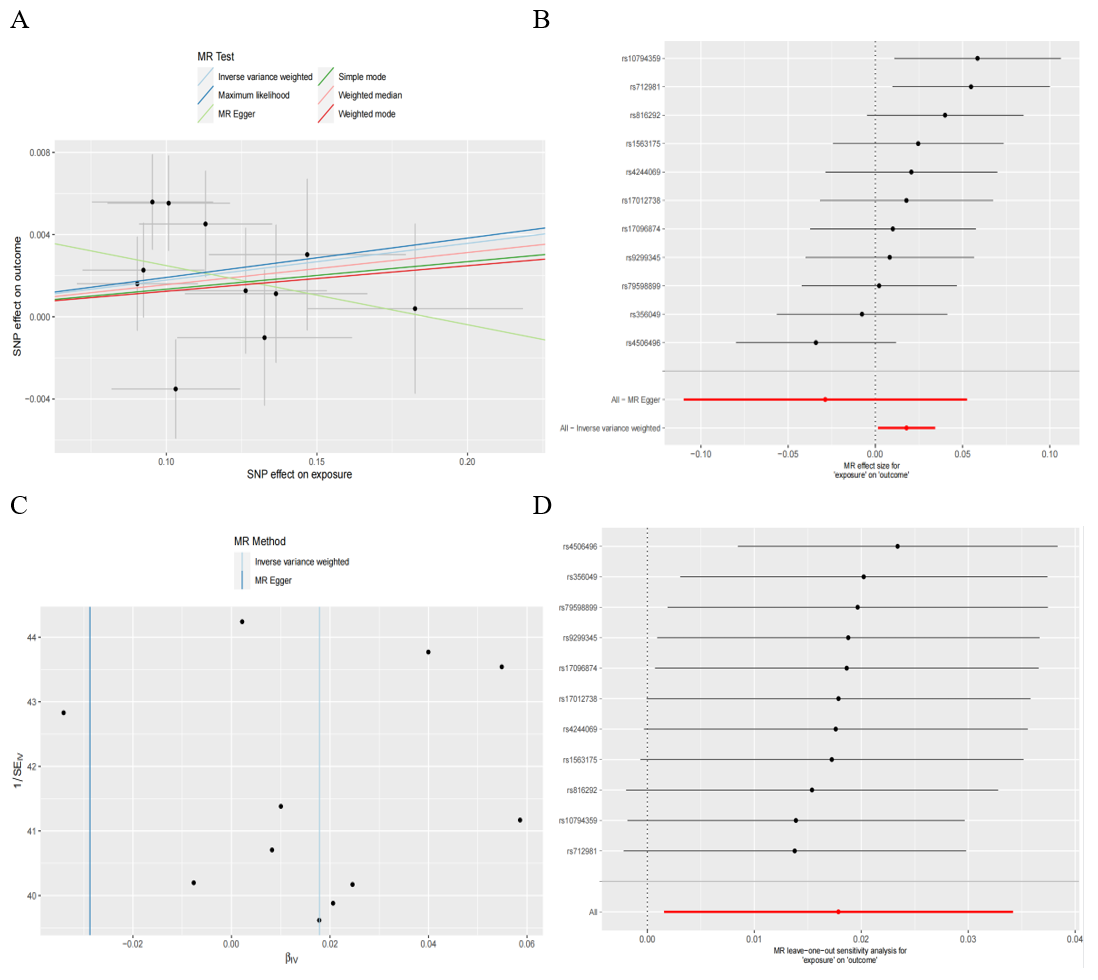
**

**Figure S11 Scatter plot (A), forest plot (B), funnel plot (C) and sensitivity analysis (D) of the causal effect of *Anaerofilum* on short sleep duration.**

**
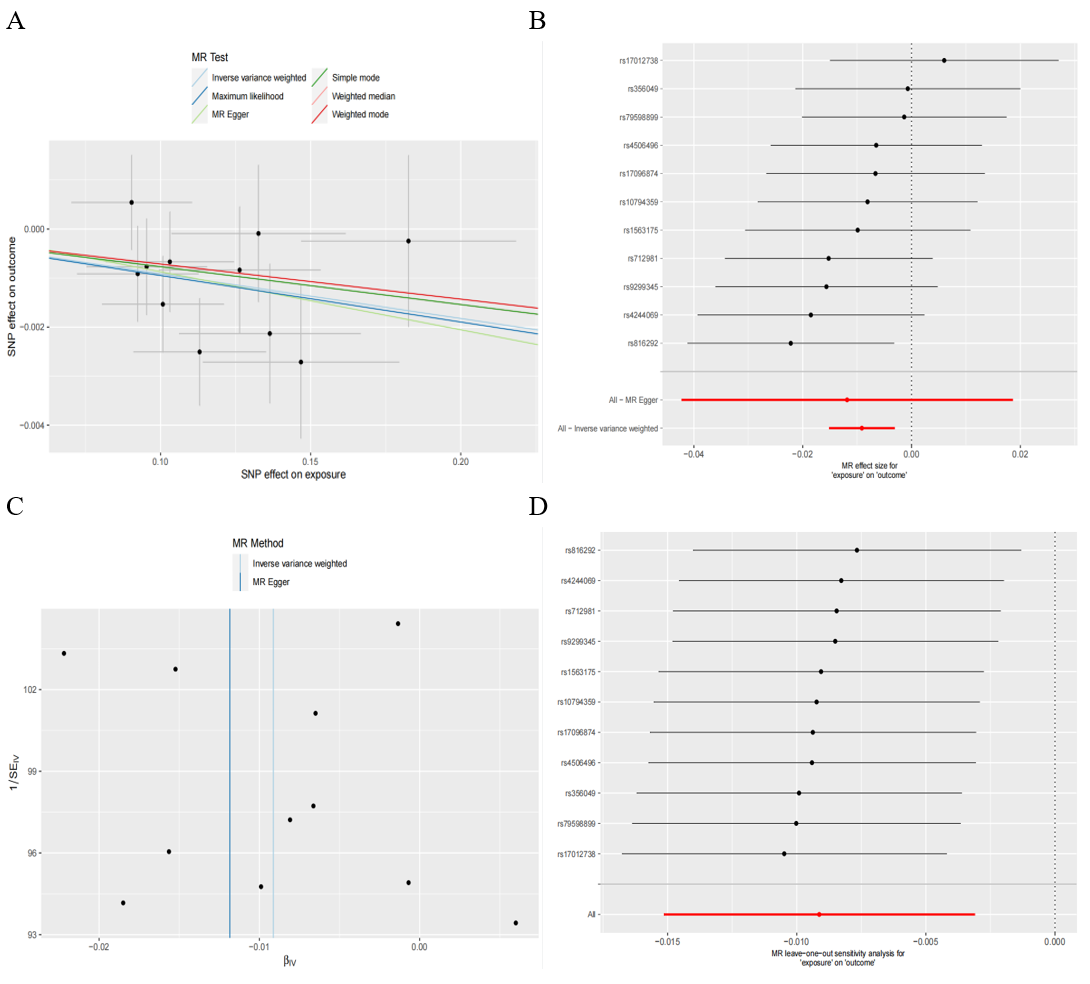
**

**Figure S12 Scatter plot (A), forest plot (B), funnel plot (C) and sensitivity analysis (D) of the causal effect of *Clostridium innocuum group* on insomnia.**

**
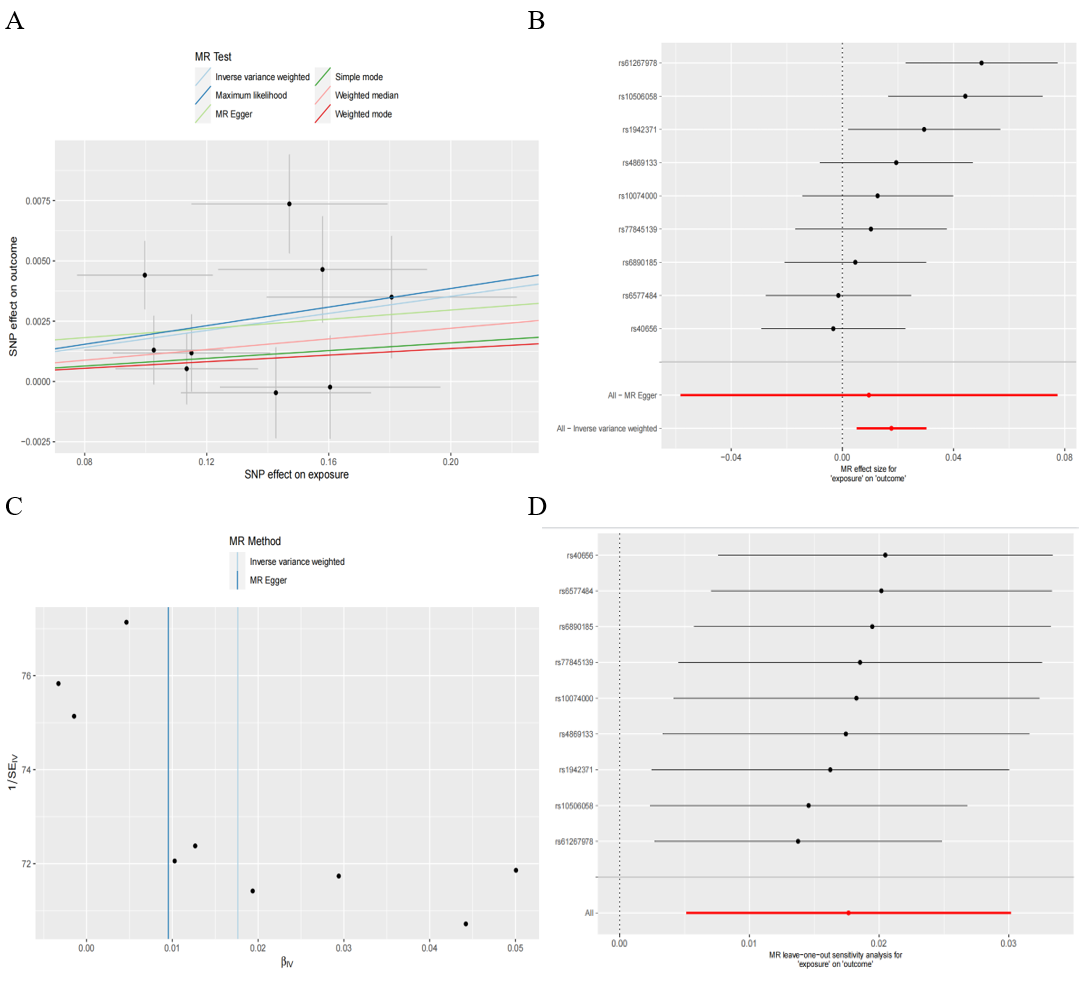
**

**Figure S13 Scatter plot (A), forest plot (B), funnel plot (C) and sensitivity analysis (D) of the causal effect of *Eubacterium hallii group* on sleep duration.**

**
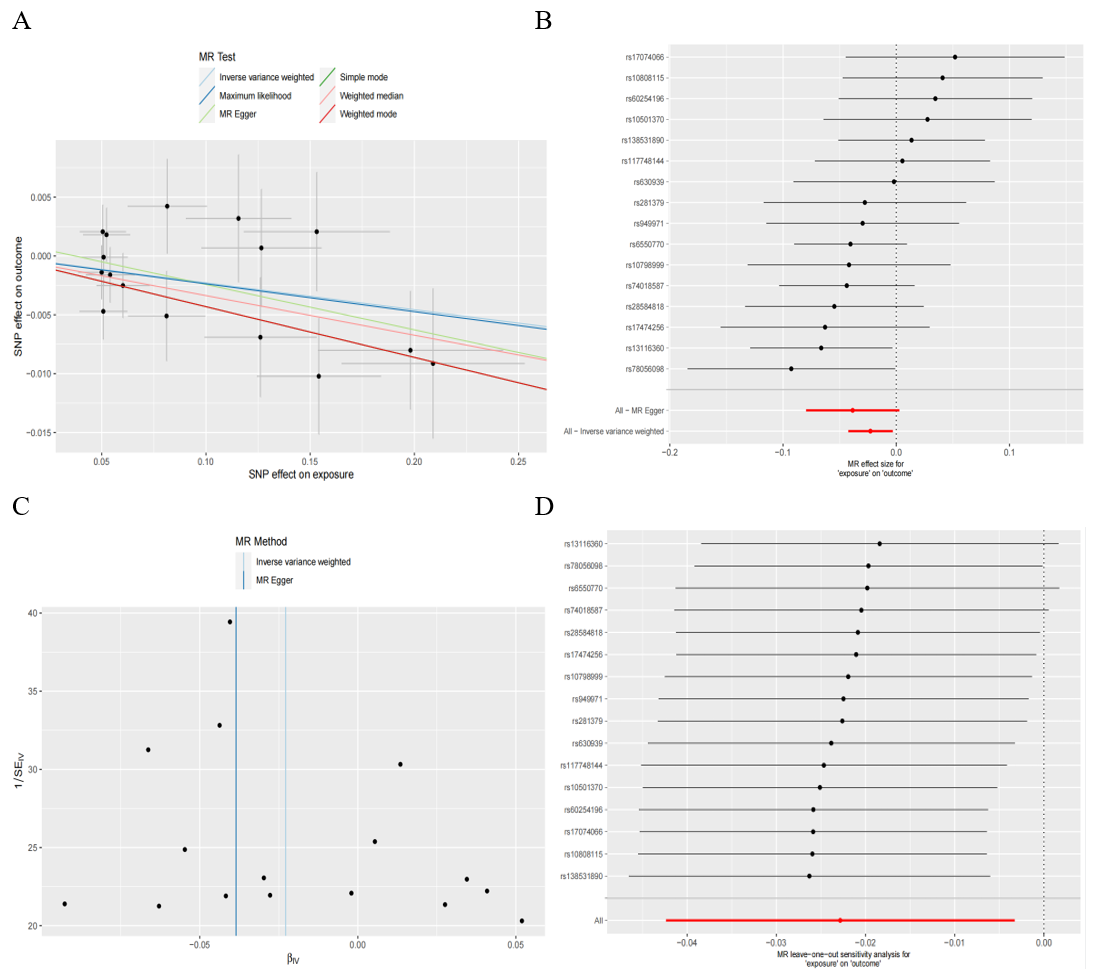
**

**Figure S14 Scatter plot (A), forest plot (B), funnel plot (C) and sensitivity analysis (D) of the causal effect of *Eubacterium hallii group* on short sleep duration.**

**
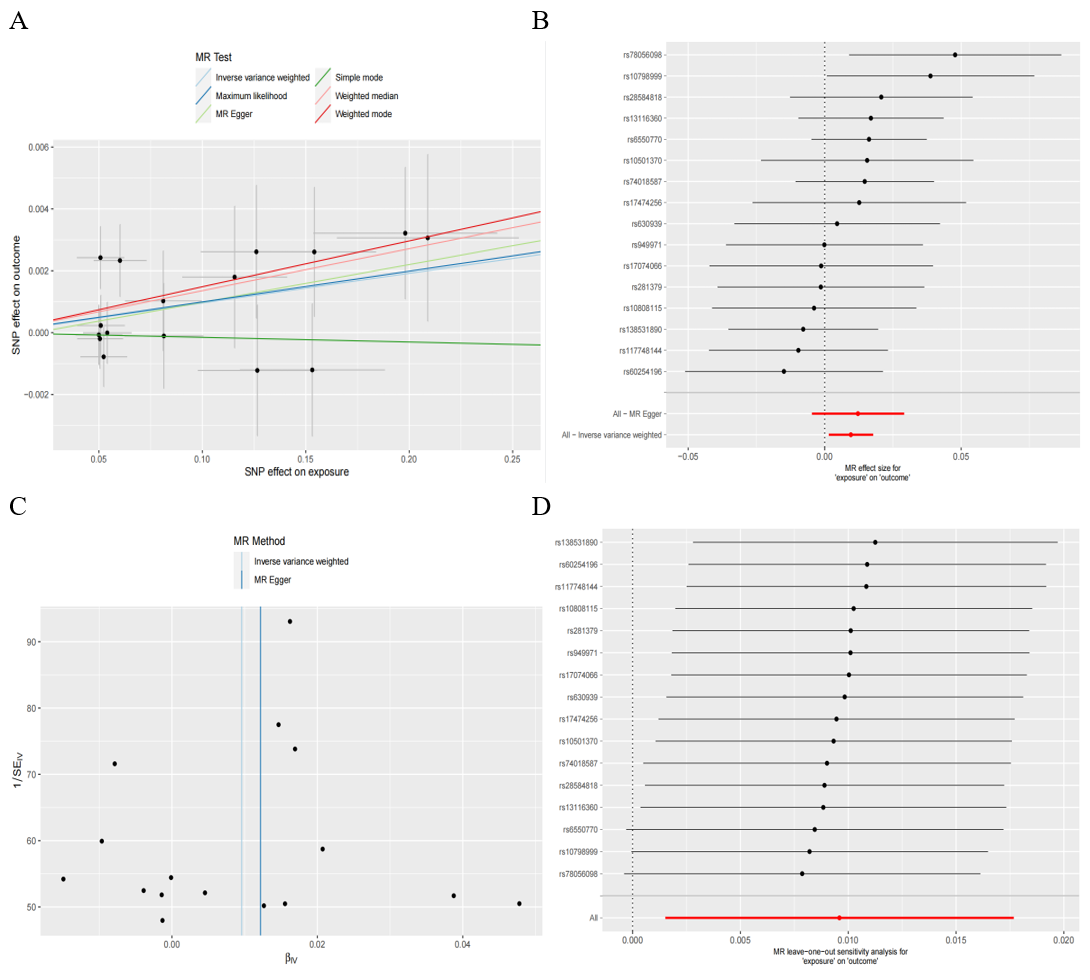
**

**Figure S15 Scatter plot (A), forest plot (B), funnel plot (C) and sensitivity analysis (D) of the causal effect of *Alistipes* on sleep duration.**

**
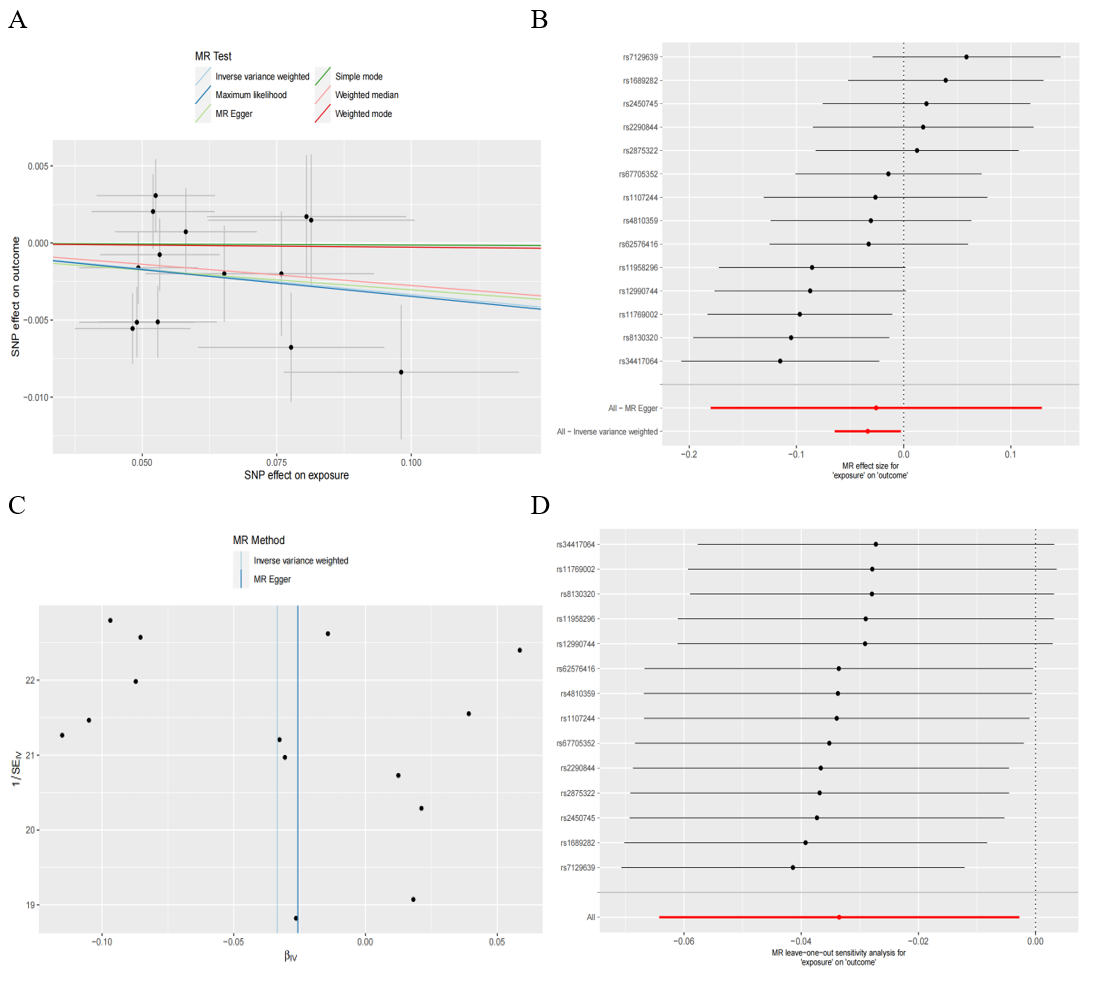
**

**Figure S16 Scatter plot (A), forest plot (B), funnel plot (C) and sensitivity analysis (D) of the causal effect of *Alistipes* on long sleep duration.**

**
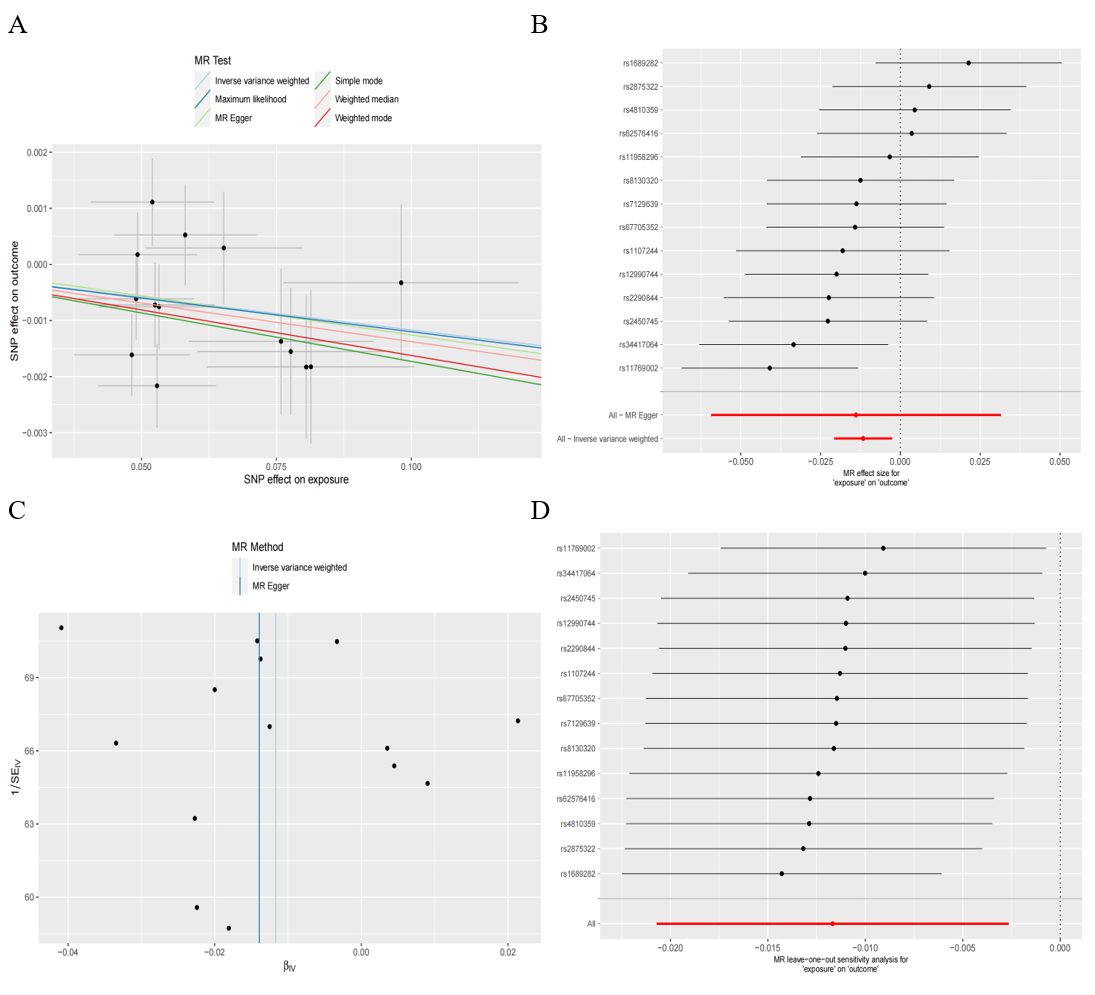
**

**Figure S17 Scatter plot (A), forest plot (B), funnel plot (C) and sensitivity analysis (D) of the causal effect of *Catenibacterium* on binary chronotype.**

**
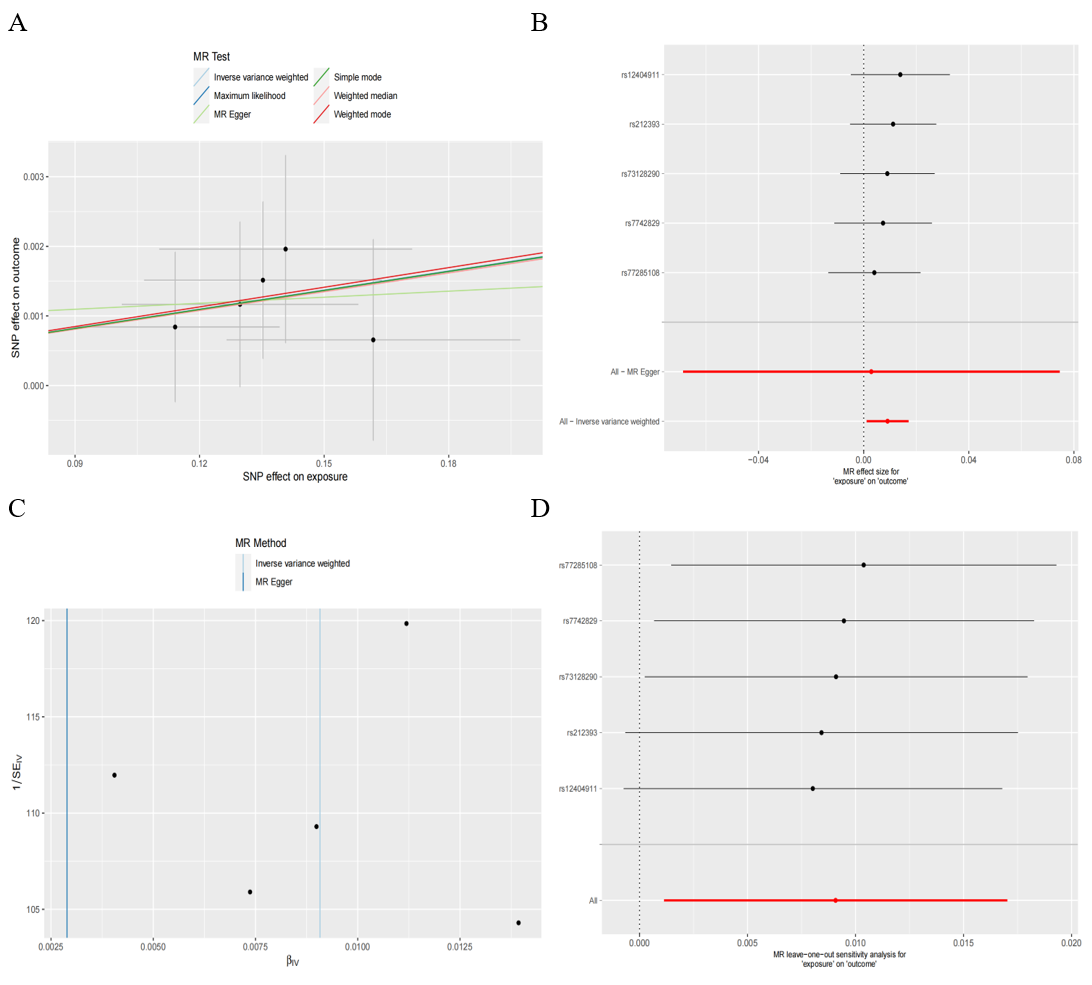
**

**Figure S18 Scatter plot (A), forest plot (B), funnel plot (C) and sensitivity analysis (D) of the causal effect of *Bacteroides* on chronotype.**

**
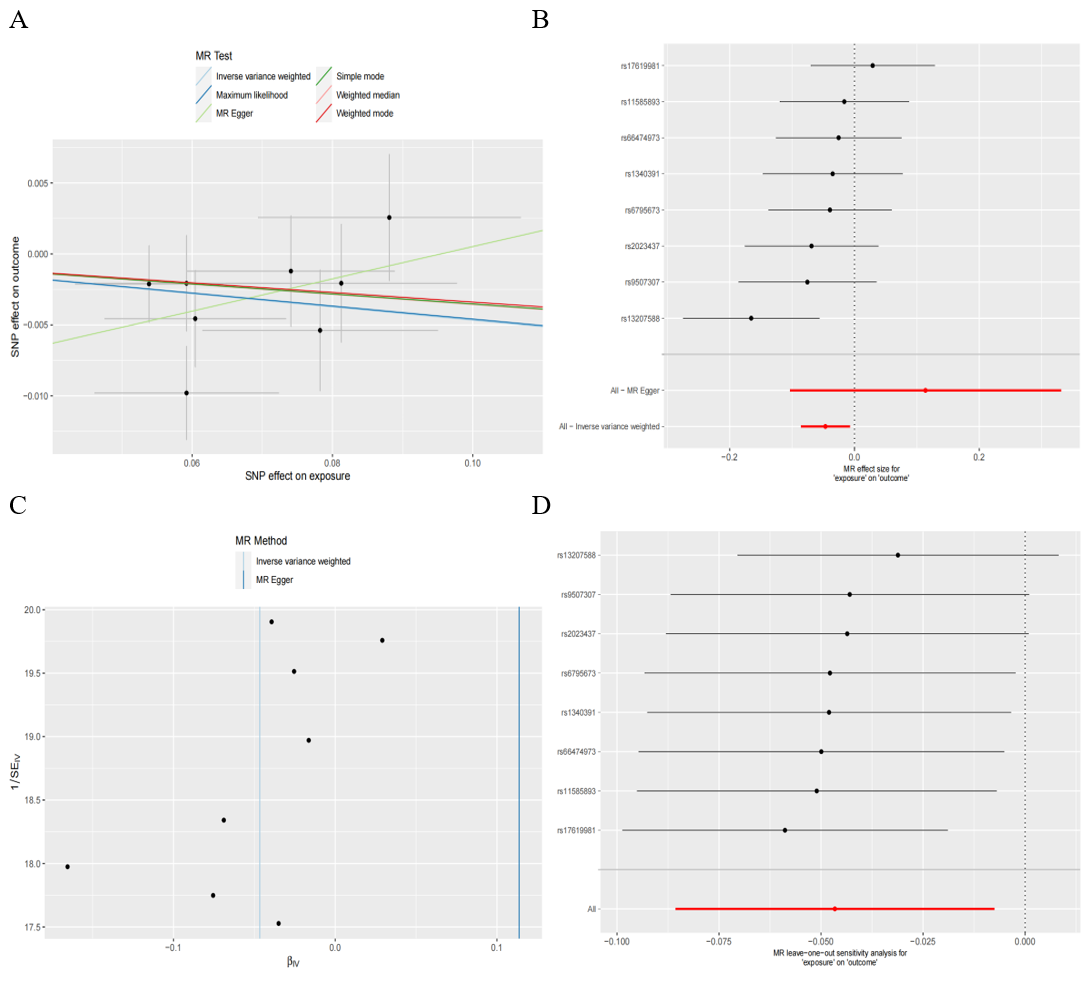
**

**Figure S19 Scatter plot (A), forest plot (B), funnel plot (C) and sensitivity analysis (D) of the causal effect of *Bacteroides* on binary chronotype.**

**
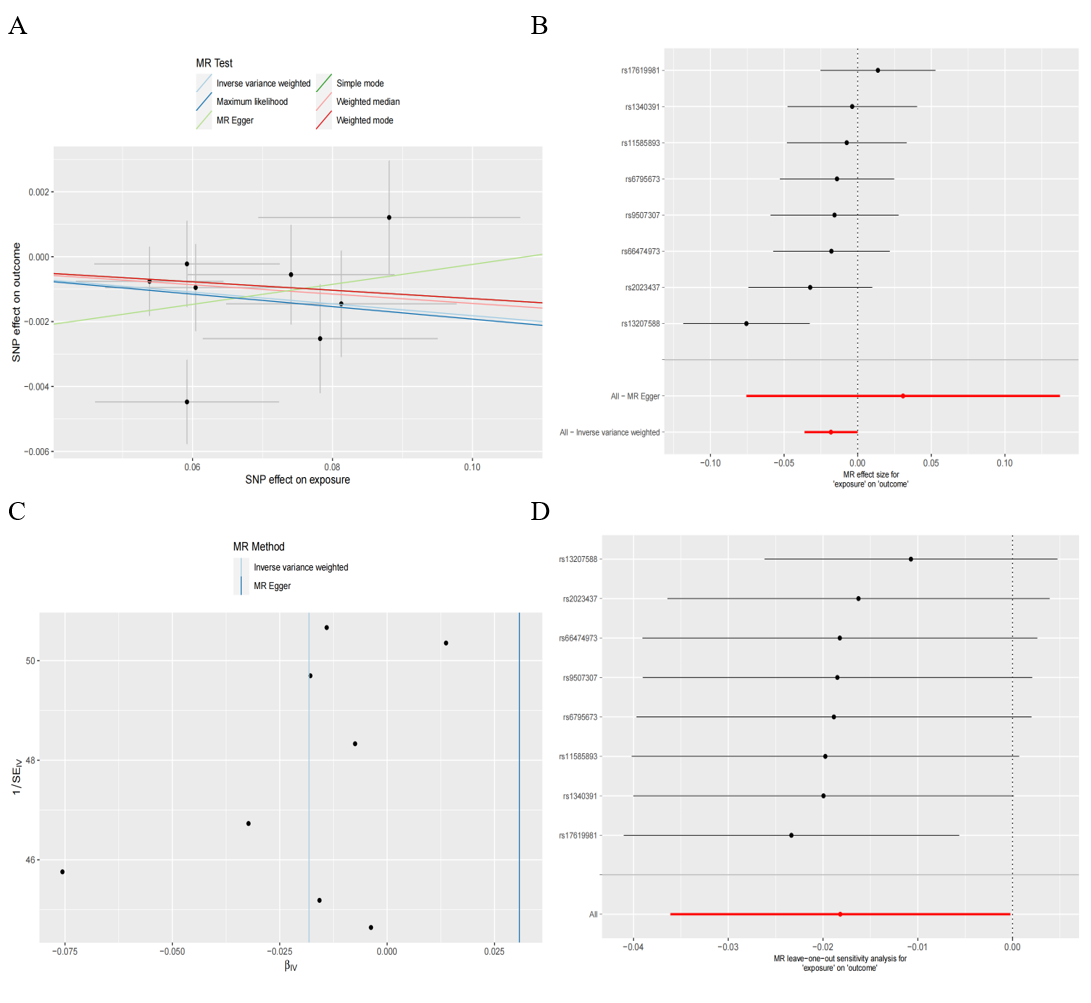
**

**Figure S20 Scatter plot (A), forest plot (B), funnel plot (C) and sensitivity analysis (D) of the causal effect of *Barnesiella* on short sleep duration.**

**
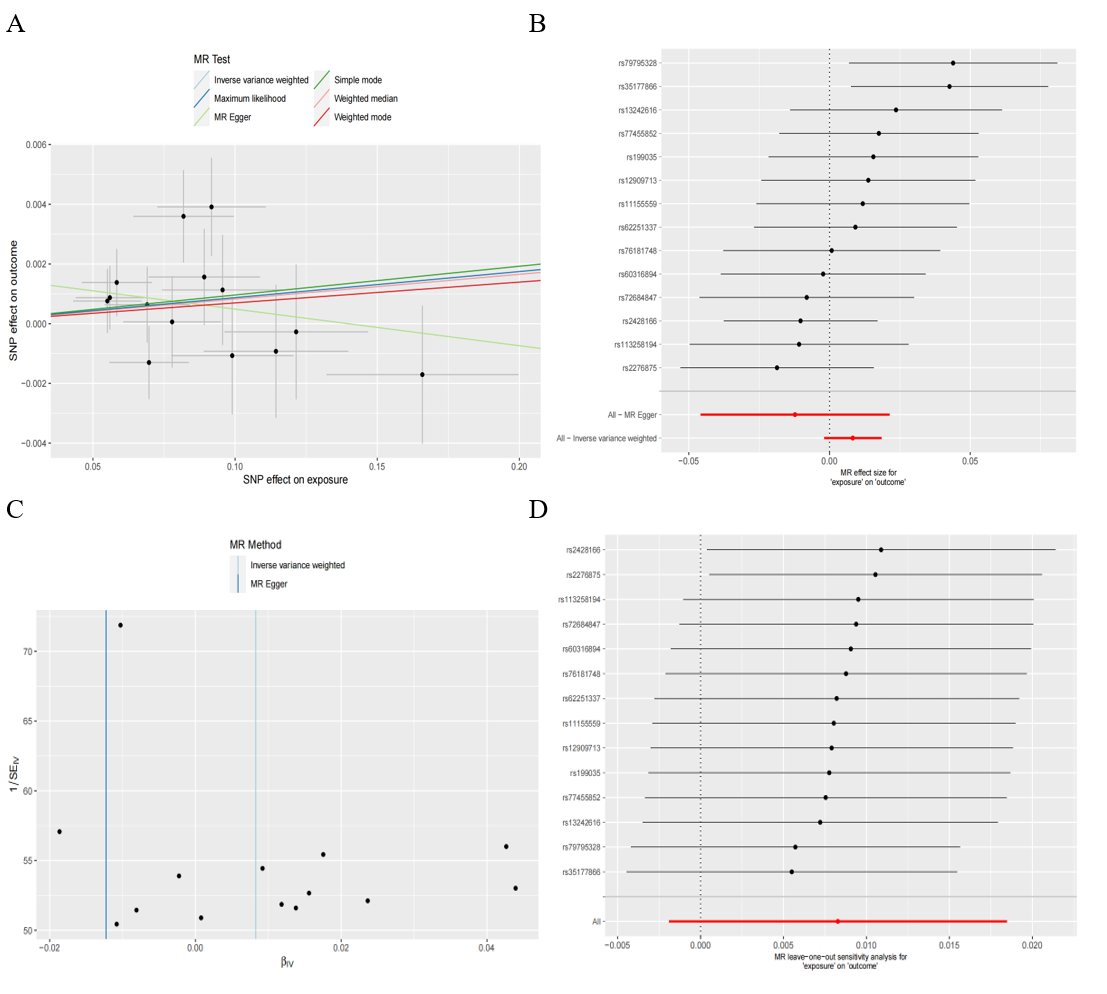
**

**Figure S21 Scatter plot (A), forest plot (B), funnel plot (C) and sensitivity analysis (D) of the causal effect of *Bifidobacterium* on binary chronotype.**

**
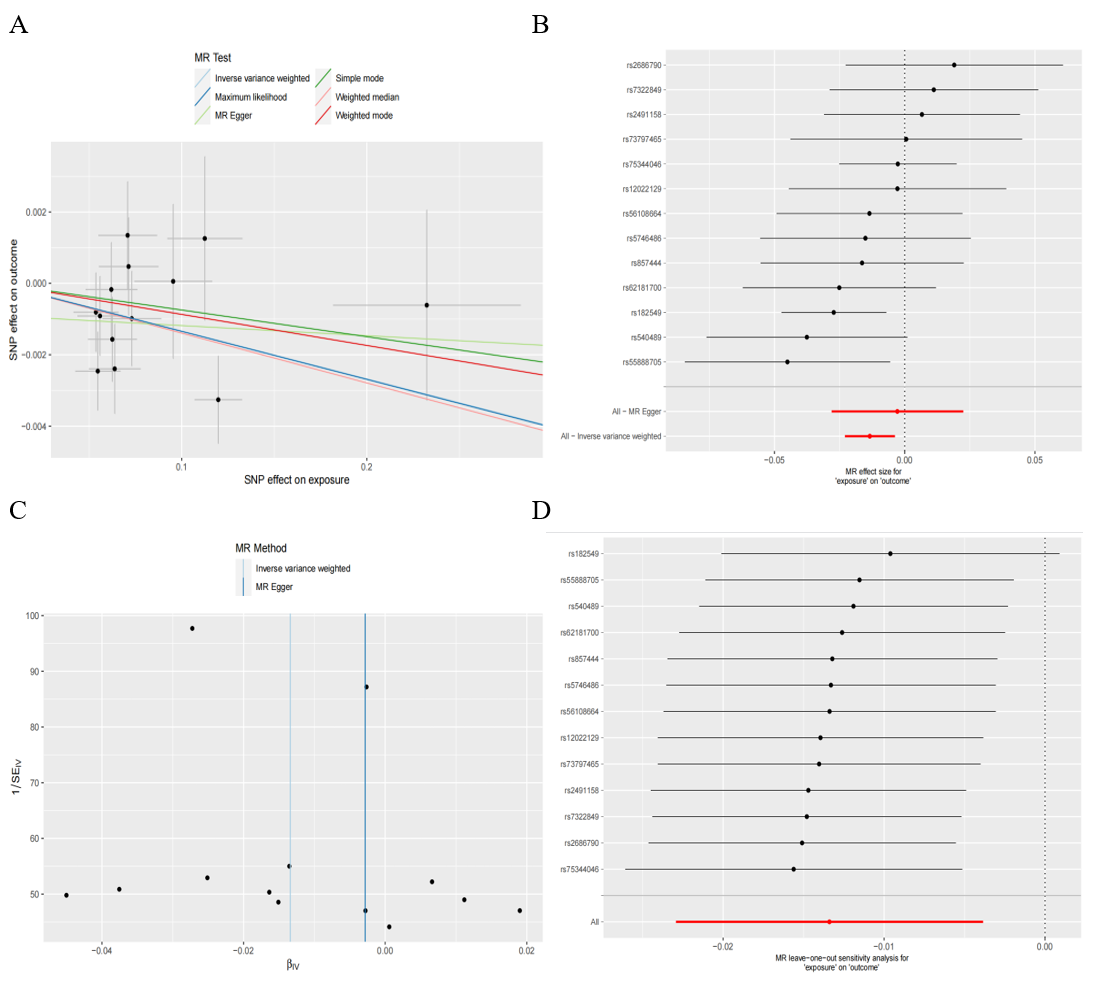
**

**Figure S22 Scatter plot (A), forest plot (B), funnel plot (C) and sensitivity analysis (D) of the causal effect of *Butyricimonas* on daytime napping.**

**
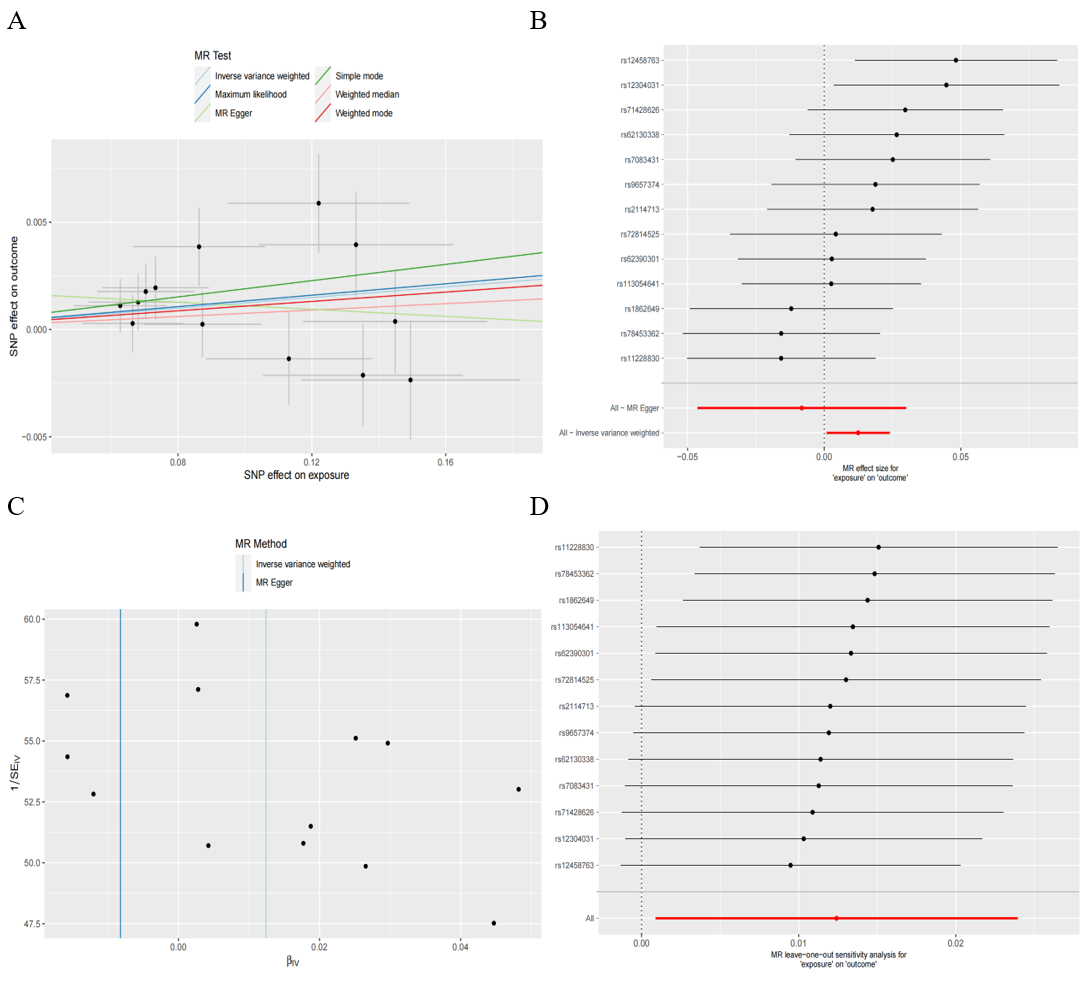
**

**Figure S23 Scatter plot (A), forest plot (B), funnel plot (C) and sensitivity analysis (D) of the causal effect of *Butyricimonas* on daytime sleepiness.**

**
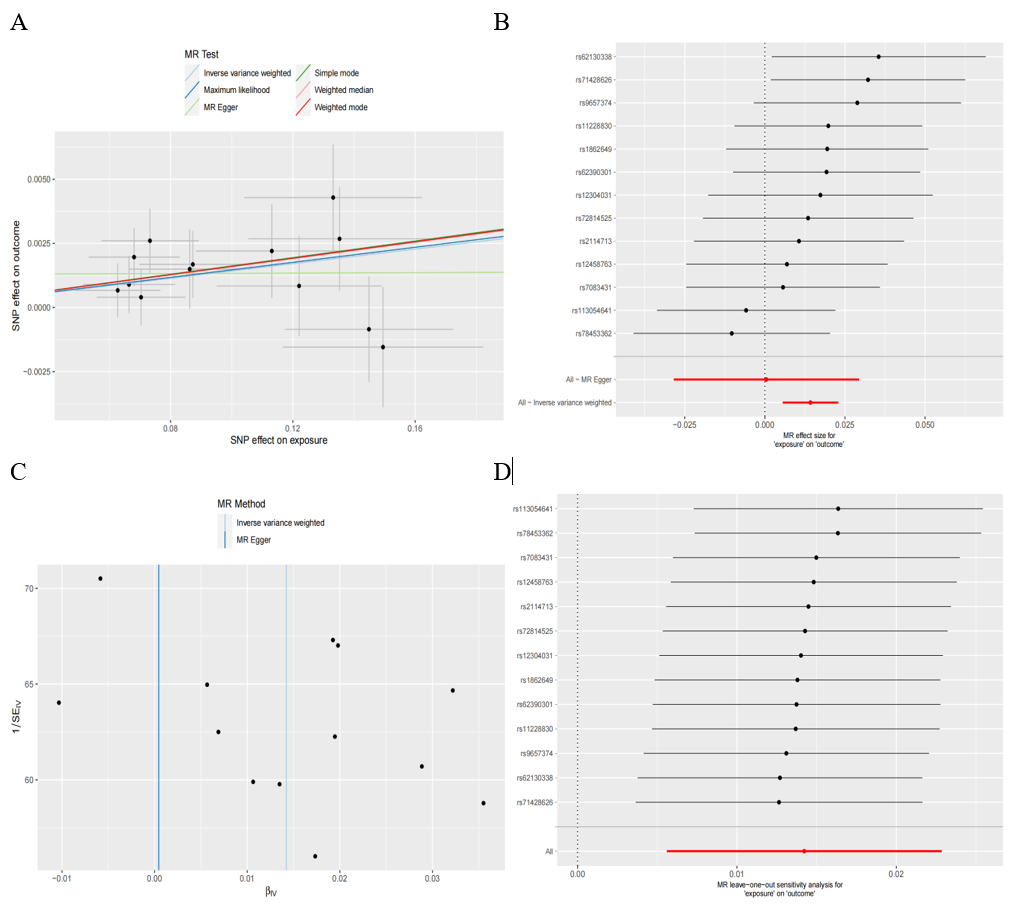
**

**Figure S24 Scatter plot (A), forest plot (B), funnel plot (C) and sensitivity analysis (D) of the causal effect of *Butyricimonas* on daytime sleepiness adjusted for BMI.**

**
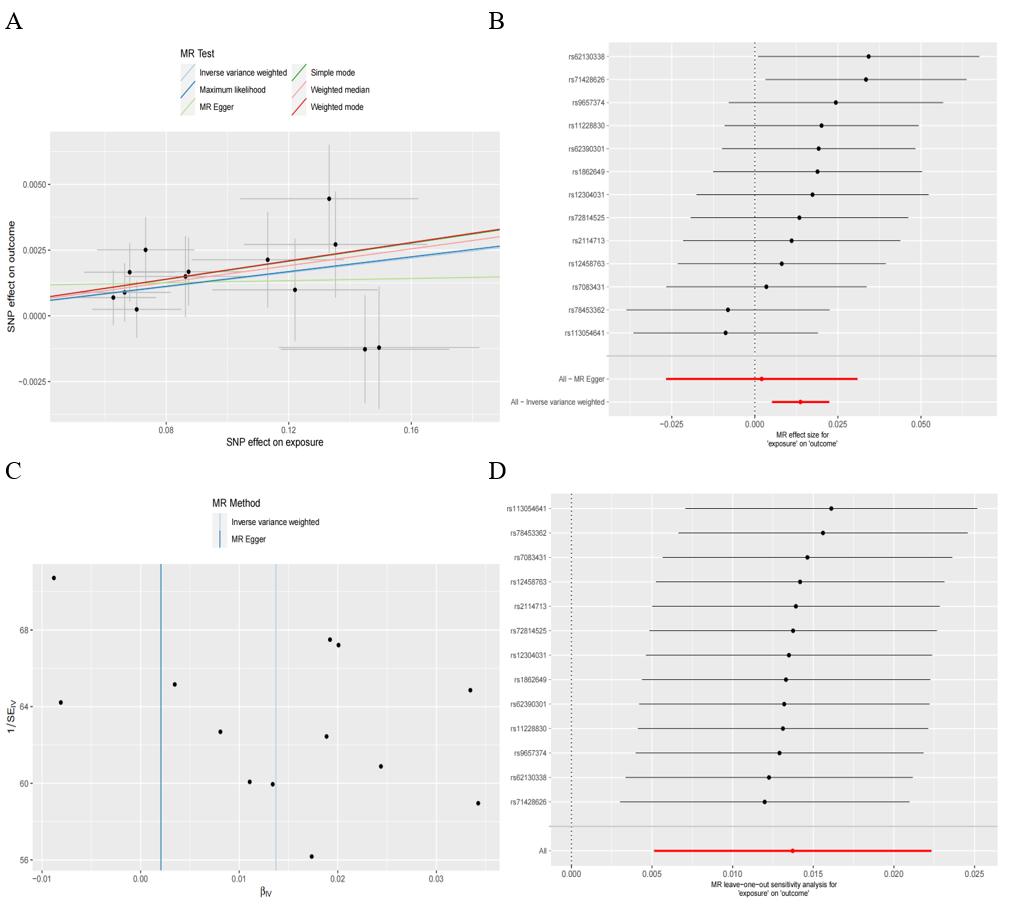
**

**Figure S25 Scatter plot (A), forest plot (B), funnel plot (C) and sensitivity analysis (D) of the causal effect of *Butyricimonas* on long sleep duration.**

**
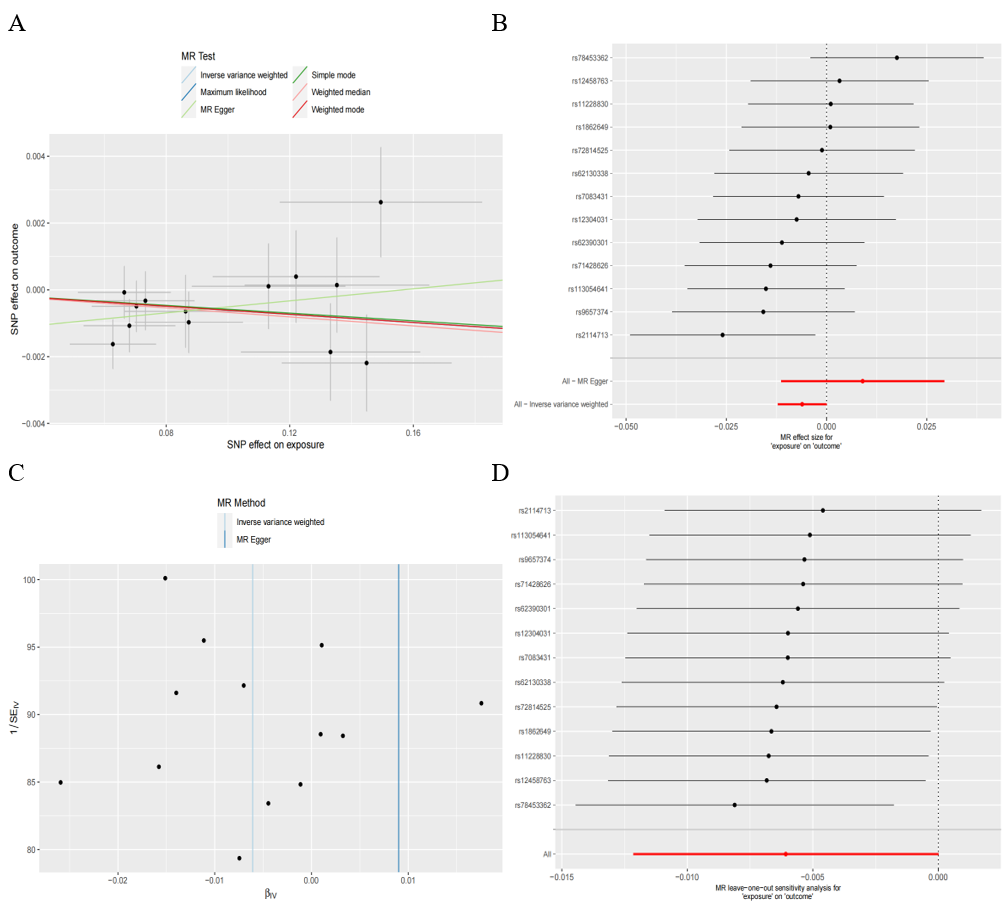
**

**Figure S26 Scatter plot (A), forest plot (B), funnel plot (C) and sensitivity analysis (D) of the causal effect of *Coprococcus 1* on short sleep duration.**

**
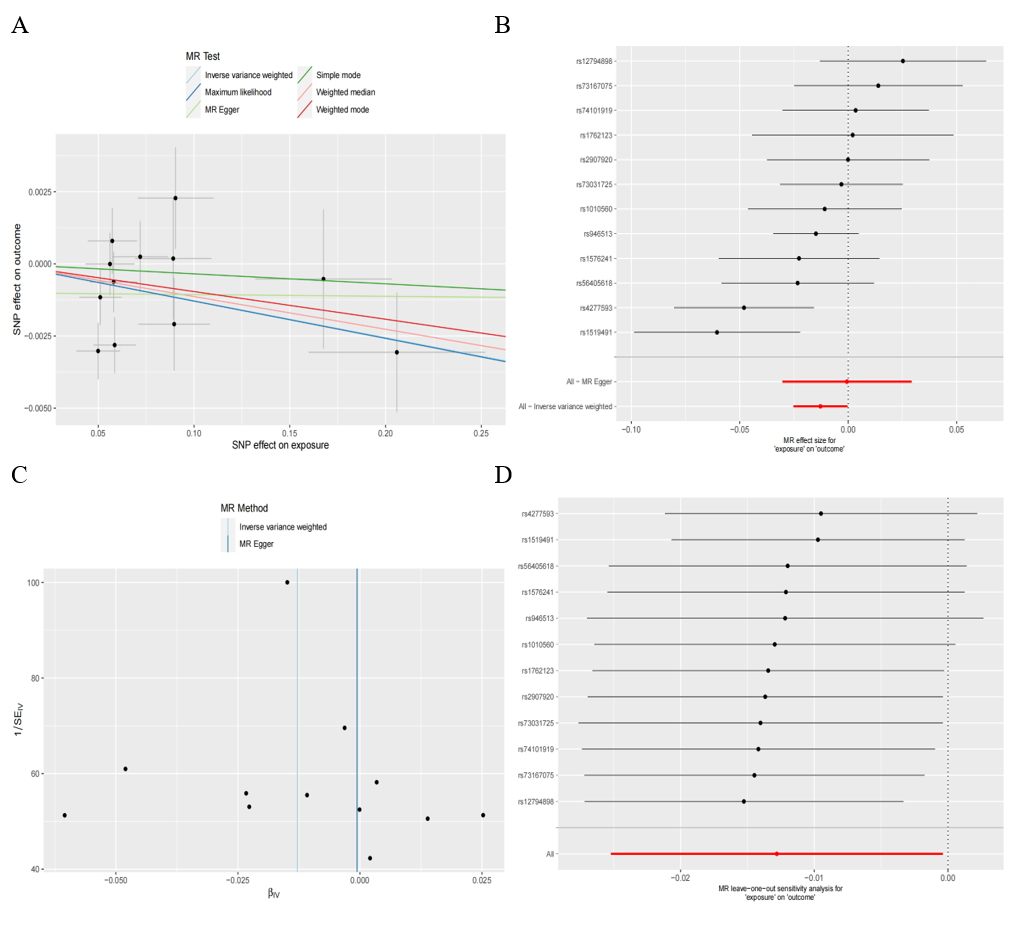
**

**Figure S27 Scatter plot (A), forest plot (B), funnel plot (C) and sensitivity analysis (D) of the causal effect of *Eisenbergiella* on daytime napping.**

**
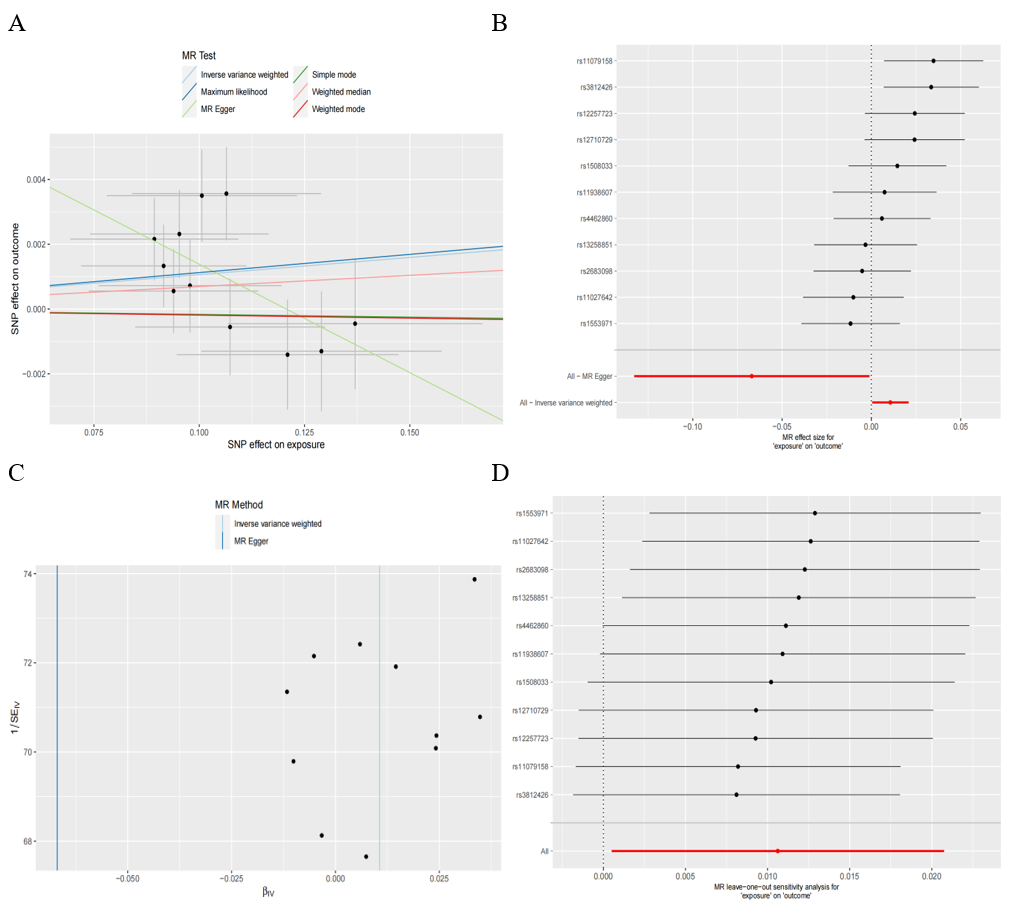
**

**Figure S28 Scatter plot (A), forest plot (B), funnel plot (C) and sensitivity analysis (D) of the causal effect of *Clostridium sensu stricto 1* on daytime sleepiness.**

**
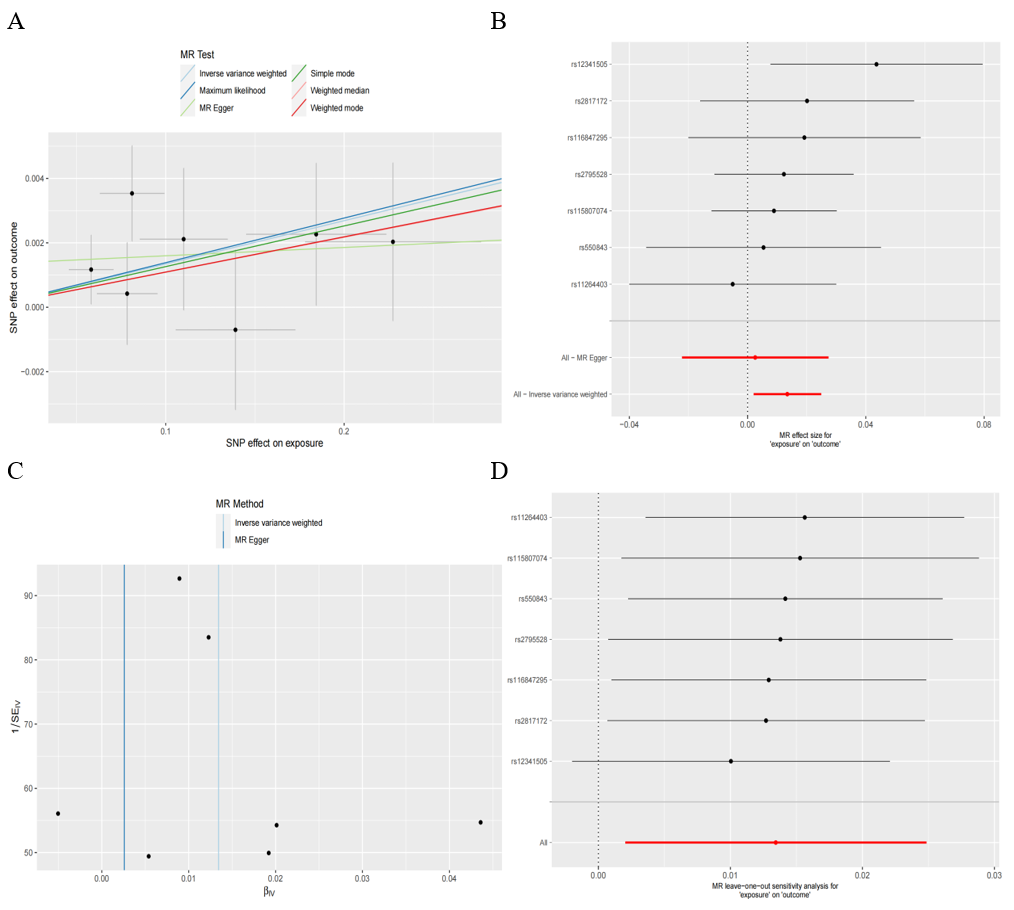
**

**Figure S29 Scatter plot (A), forest plot (B), funnel plot (C) and sensitivity analysis (D) of the causal effect of *Clostridium sensu stricto 1* on daytime sleepiness adjusted for BMI.**

**
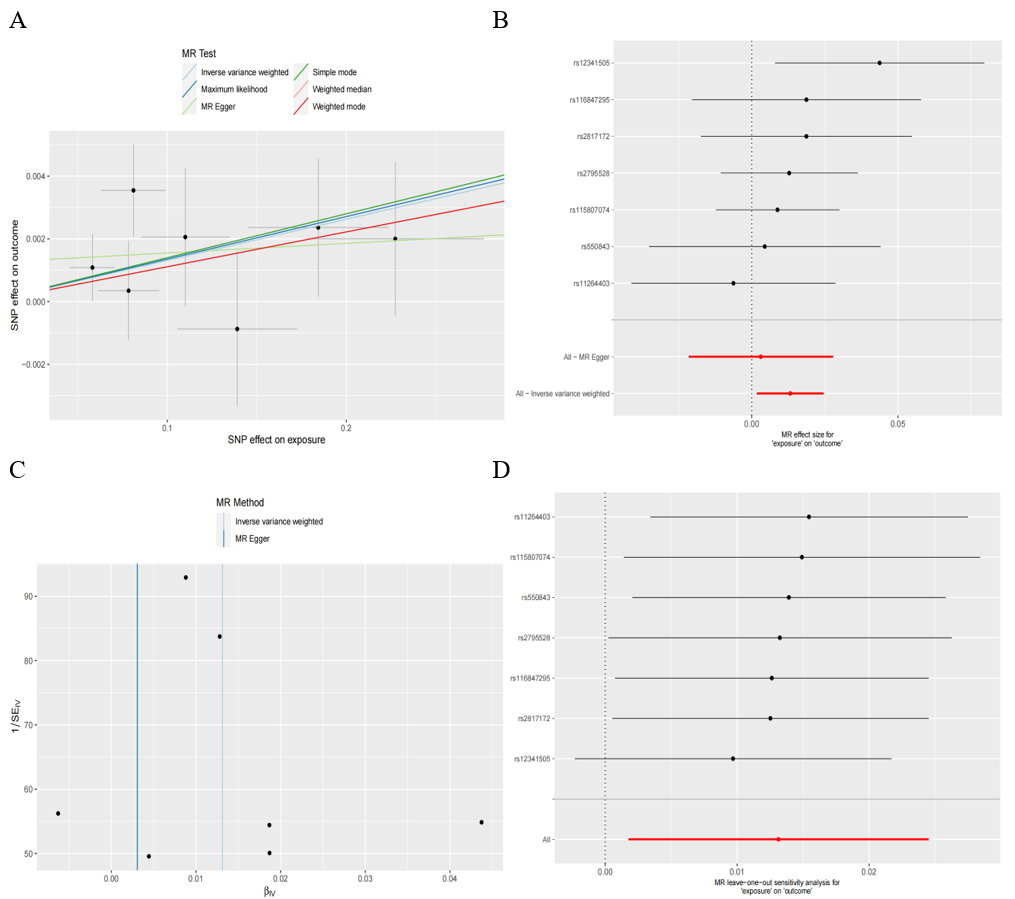
**

**Figure S30 Scatter plot (A), forest plot (B), funnel plot (C) and sensitivity analysis (D) of the causal effect of *Defluviitaleaceae UCG011* on daytime napping.**

**
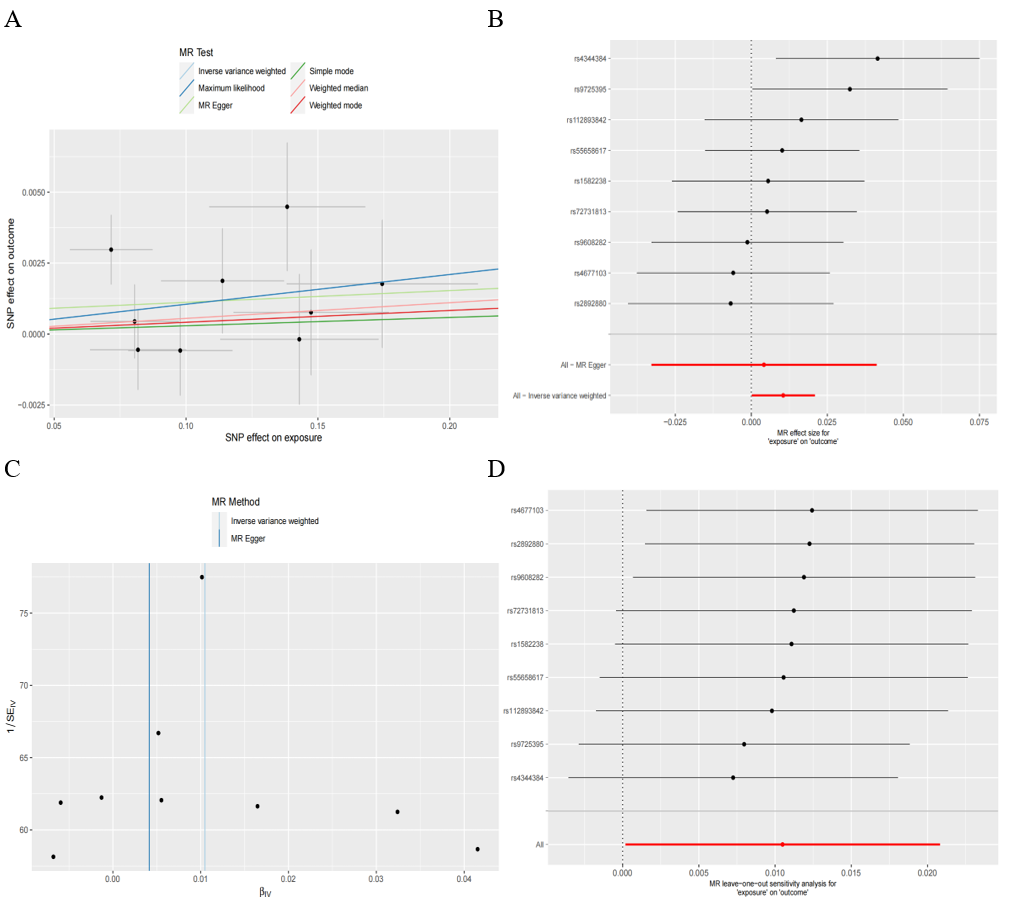
**

**Figure S31 Scatter plot (A), forest plot (B), funnel plot (C) and sensitivity analysis (D) of the causal effect of *Victivallis* on sleep duration.**

**
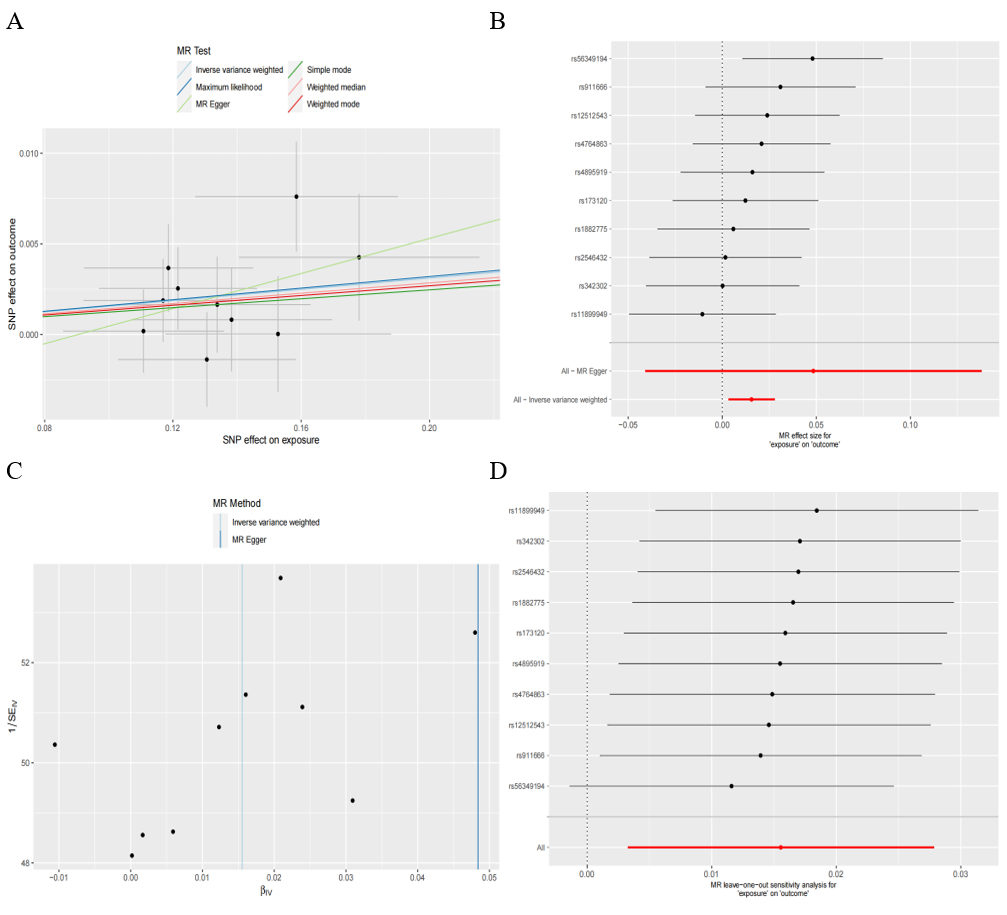
**

**Figure S32 Scatter plot (A), forest plot (B), funnel plot (C) and sensitivity analysis (D) of the causal effect of *Victivallis* on binary chronotype.**

**
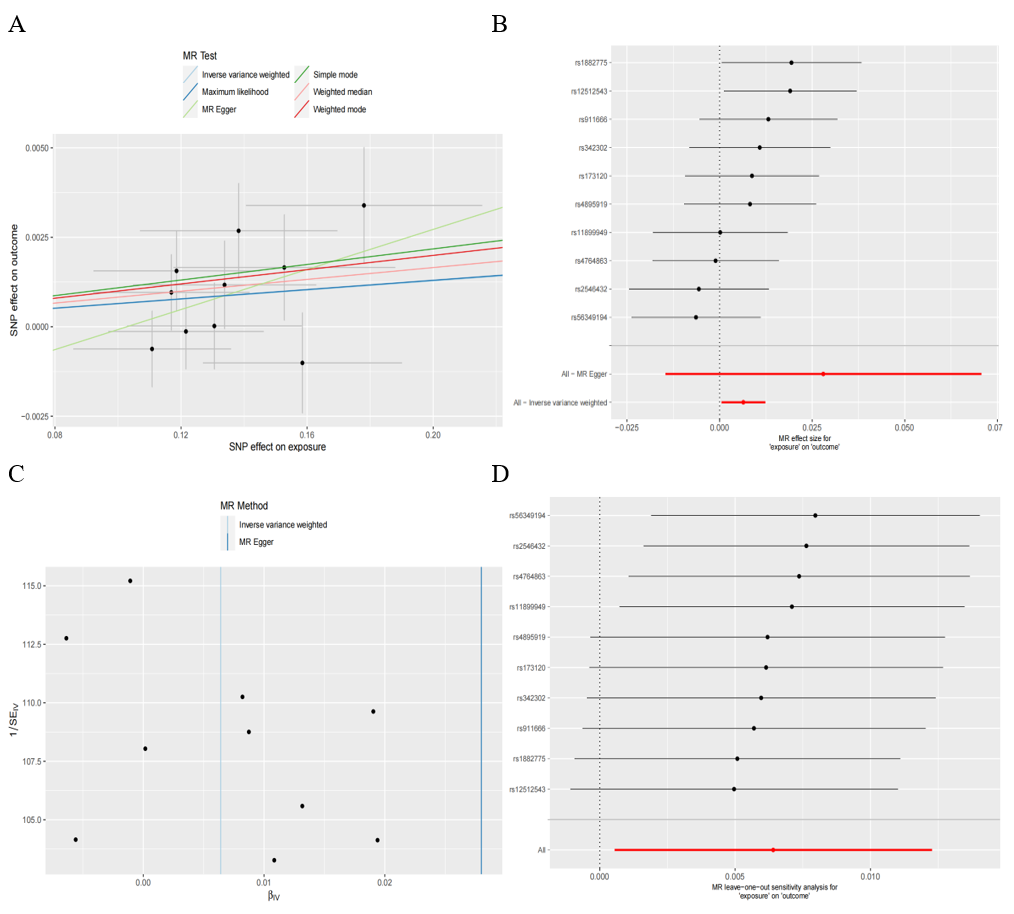
**

**Figure S33 Scatter plot (A), forest plot (B), funnel plot (C) and sensitivity analysis (D) of the causal effect of *Collinsella* on daytime sleepiness.**

**
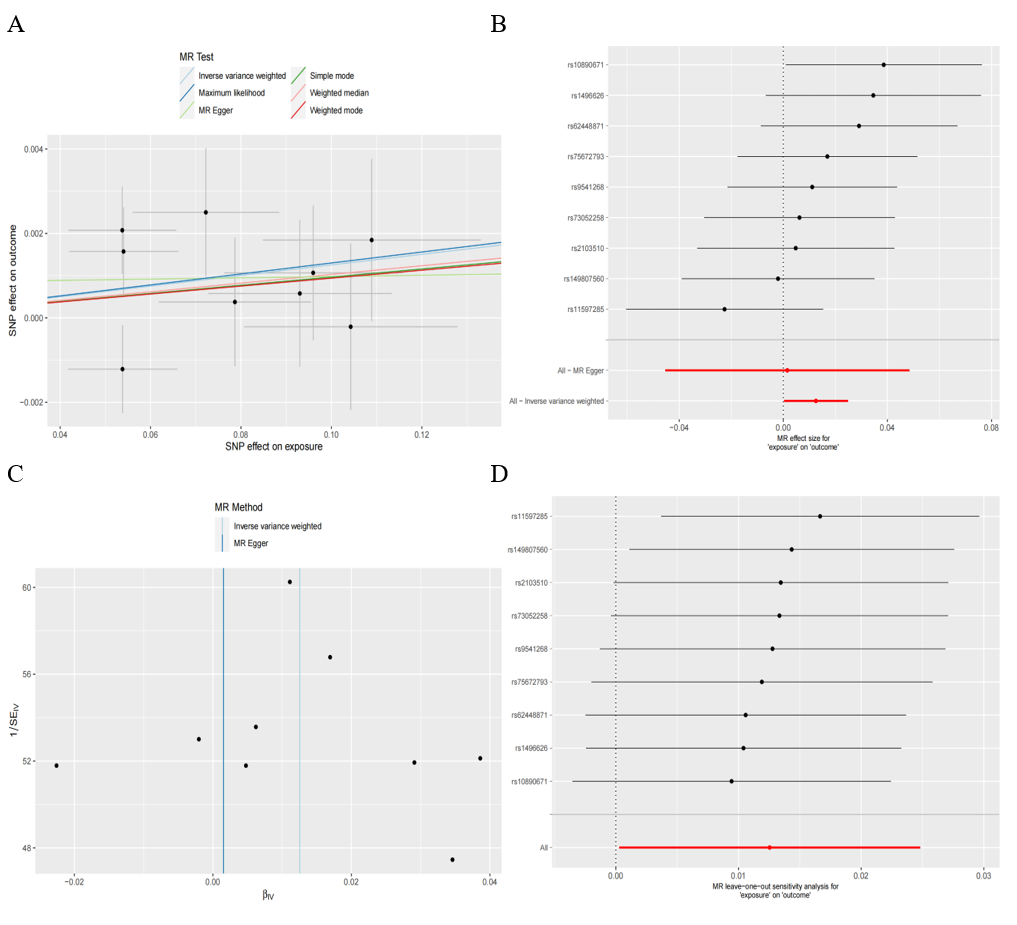
**

**Figure S34 Scatter plot (A), forest plot (B), funnel plot (C) and sensitivity analysis (D) of the causal effect of *Collinsella* on daytime sleepiness adjusted for BMI**

**
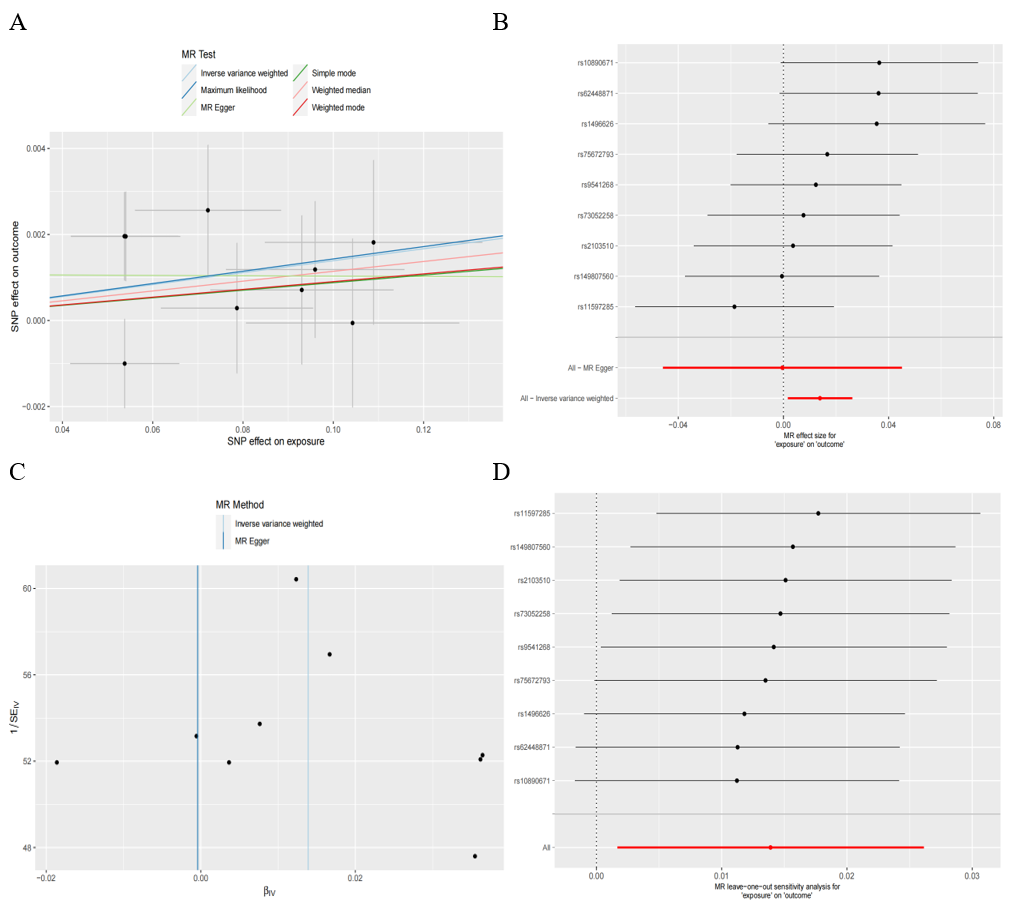
**

**Figure S35 Scatter plot (A), forest plot (B), funnel plot (C) and sensitivity analysis (D) of the causal effect of *Collinsella* on short sleep duration.**

**
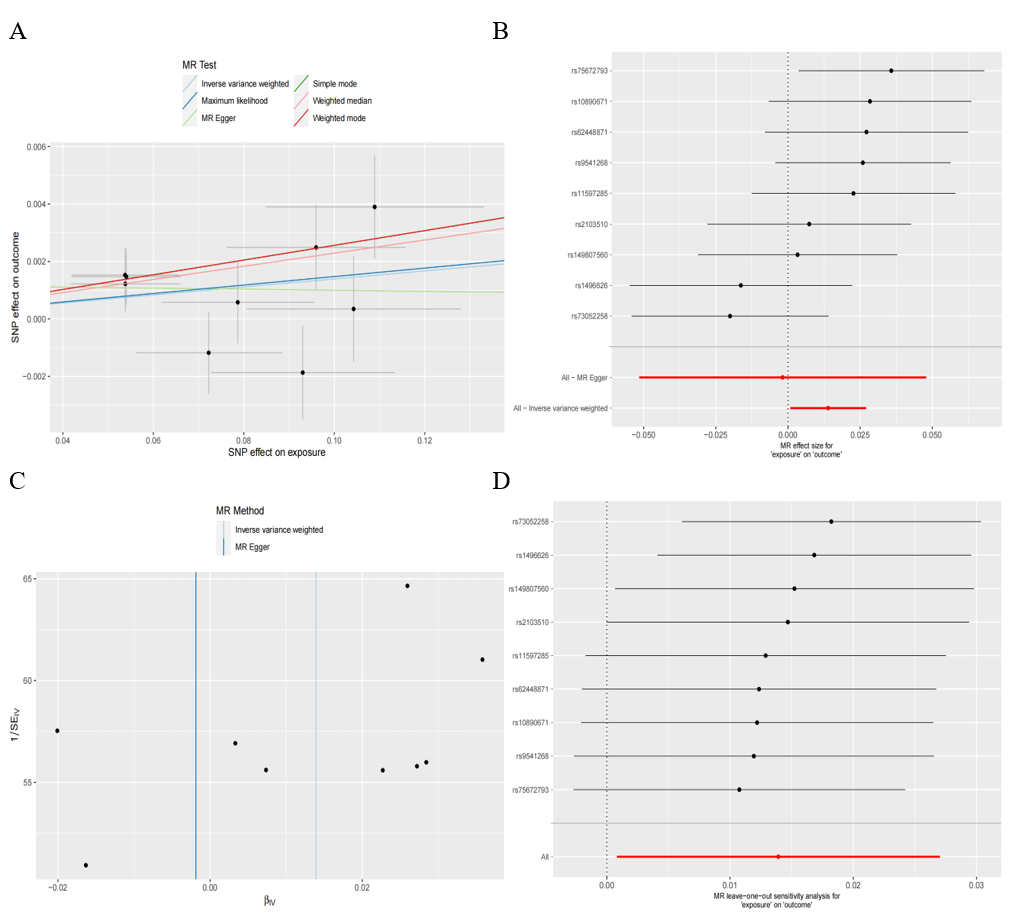
**

**Figure S36 Scatter plot (A), forest plot (B), funnel plot (C) and sensitivity analysis (D) of the causal effect of *Coprococcus 2* on daytime sleepiness.**

**
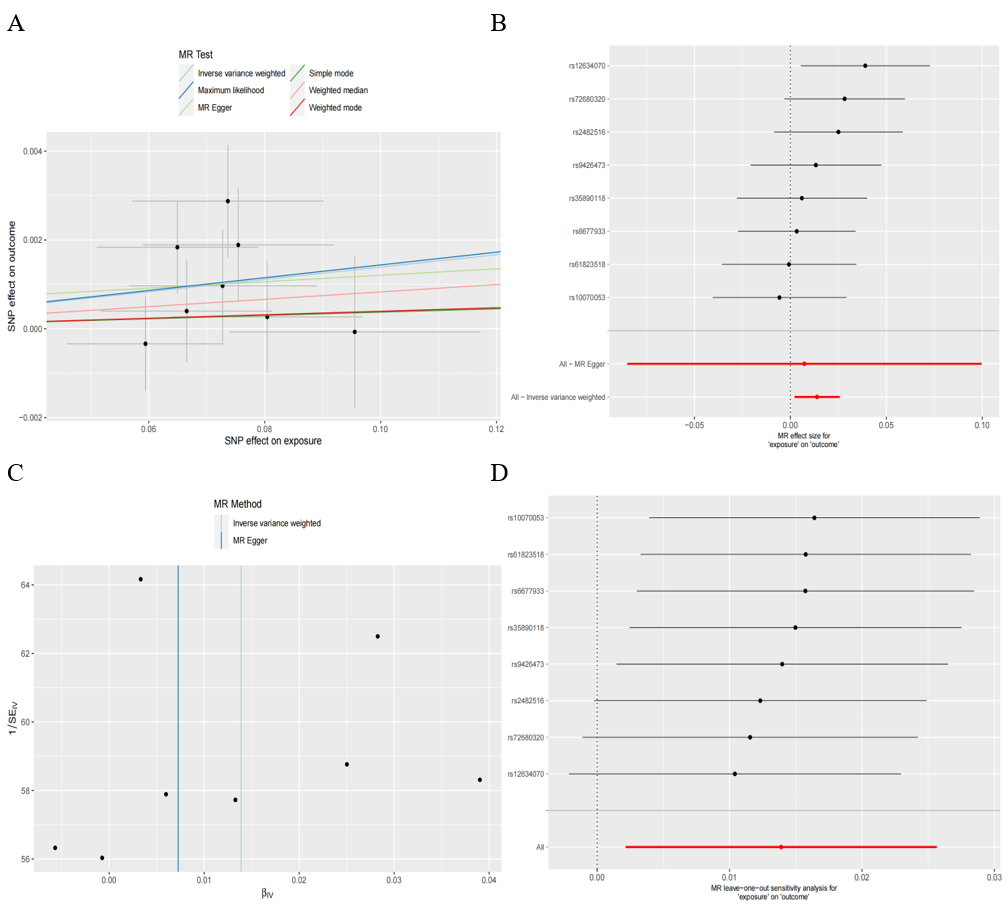
**

**Figure S37 Scatter plot (A), forest plot (B), funnel plot (C) and sensitivity analysis (D) of the causal effect of *Coprococcus 2* on daytime sleepiness adjusted for BMI.**

**
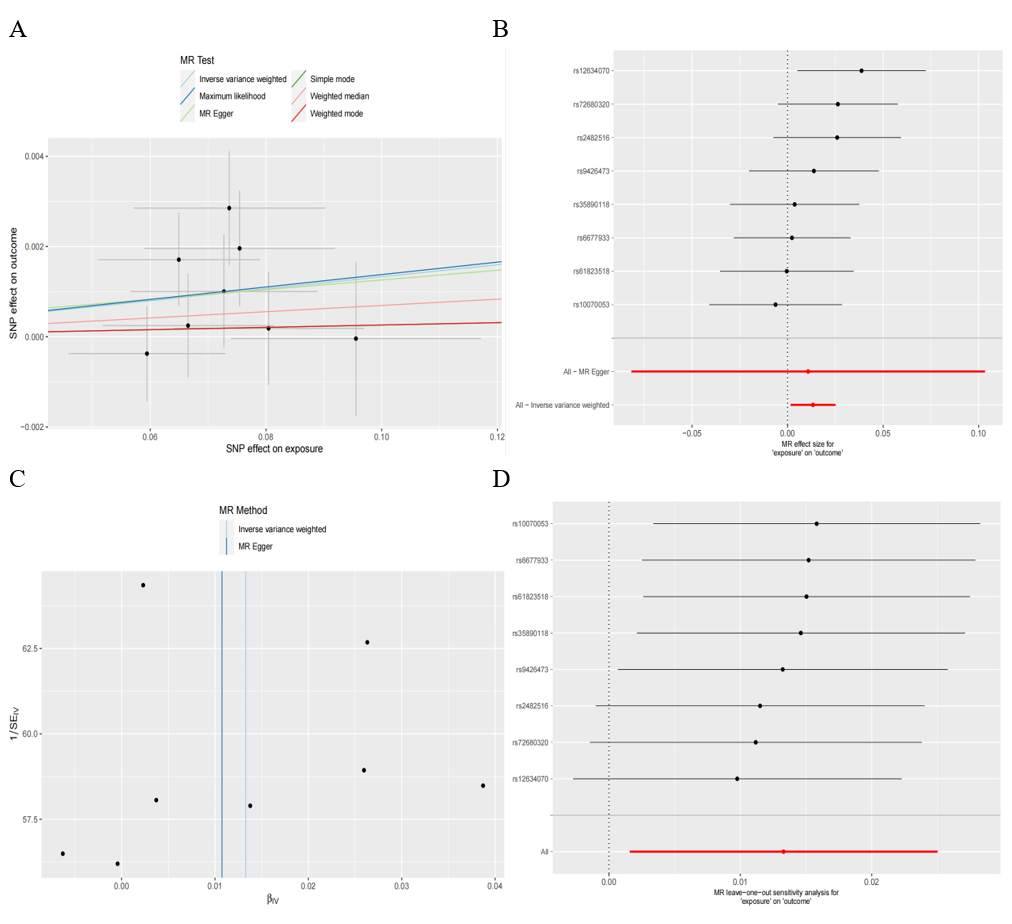
**

**Figure S38 Scatter plot (A), forest plot (B), funnel plot (C) and sensitivity analysis (D) of the causal effect of *Coprococcus 3* on daytime sleepiness.**

**
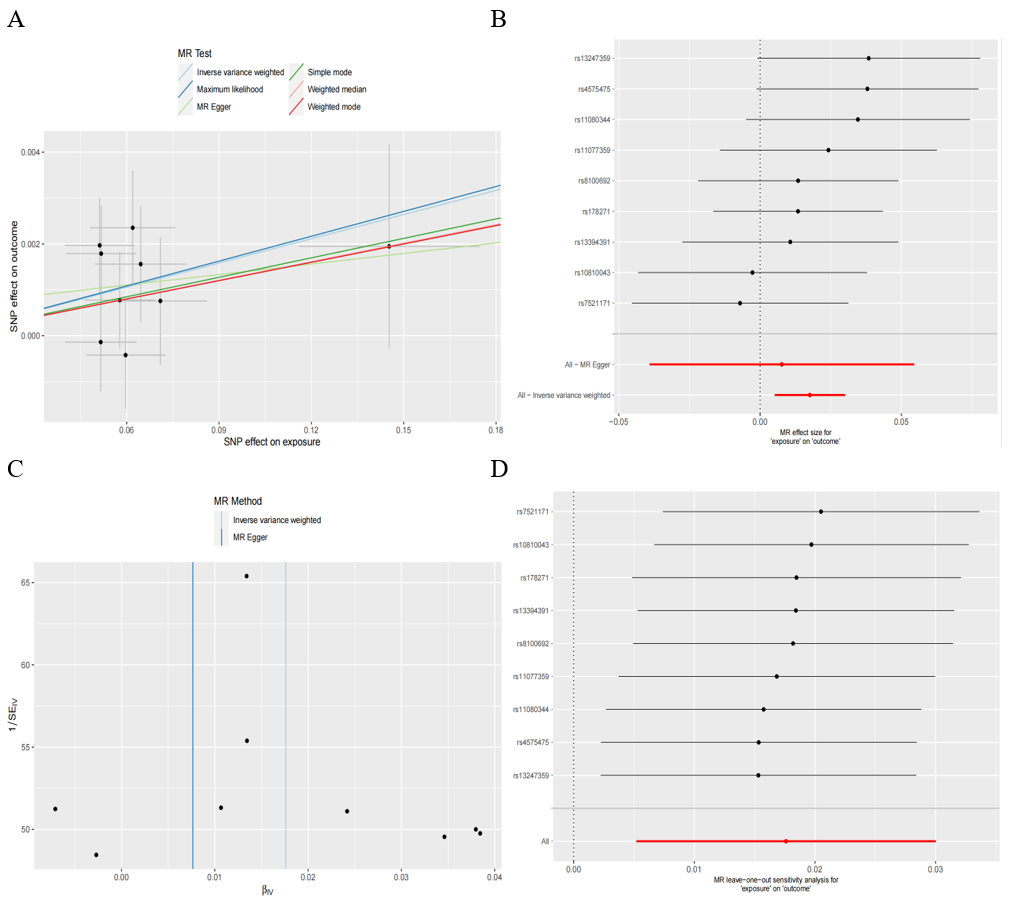
**

**Figure S39 Scatter plot (A), forest plot (B), funnel plot (C) and sensitivity analysis (D) of the causal effect of *Coprococcus 3* on daytime sleepiness adjusted for BMI.**

**
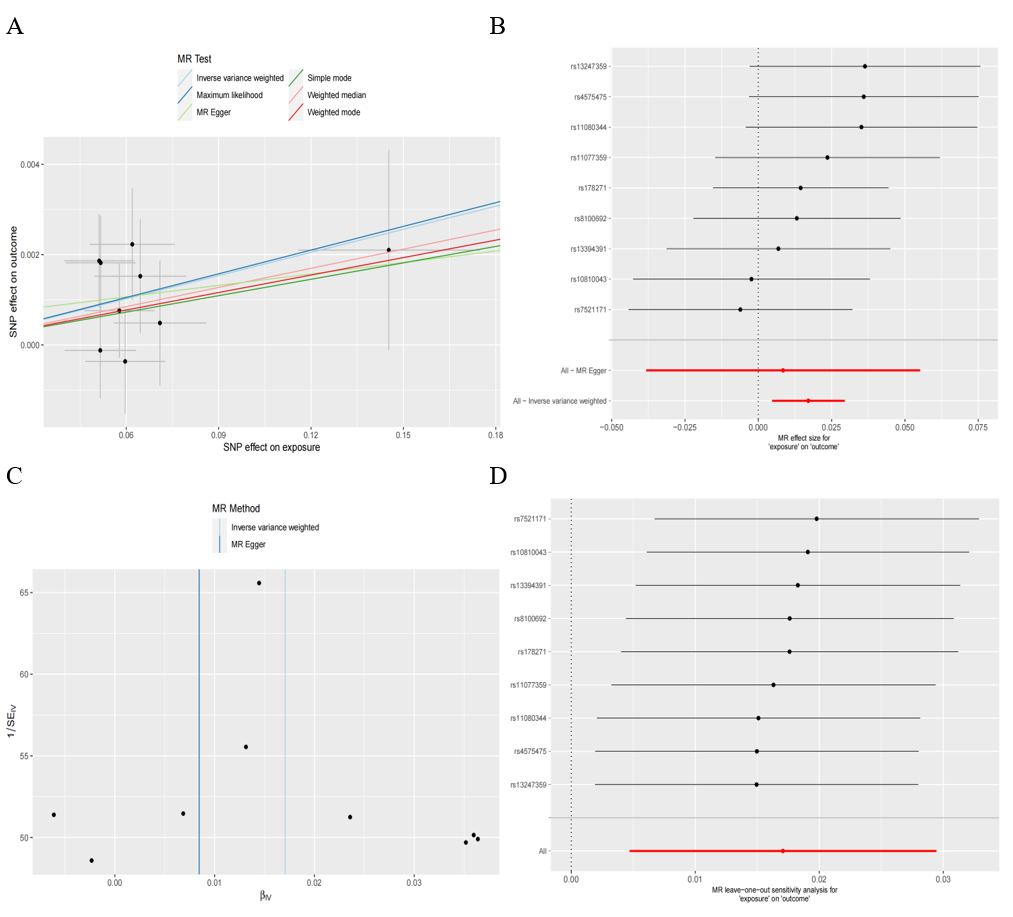
**

**Figure S40 Scatter plot (A), forest plot (B), funnel plot (C) and sensitivity analysis (D) of the causal effect of *Holdemanella* on daytime napping.**

**
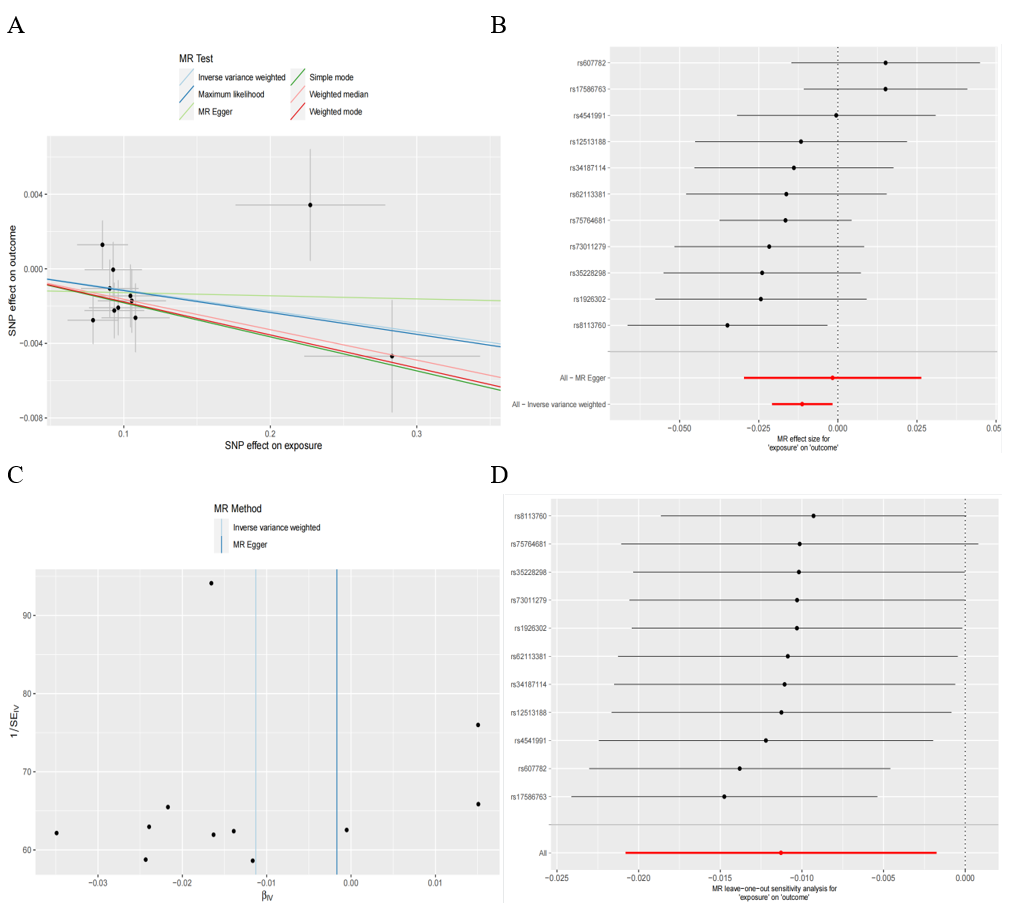
**

**Figure S41 Scatter plot (A), forest plot (B), funnel plot (C) and sensitivity analysis (D) of the causal effect of *Rikenellaceae RC9 gut group* on insomnia.**

**
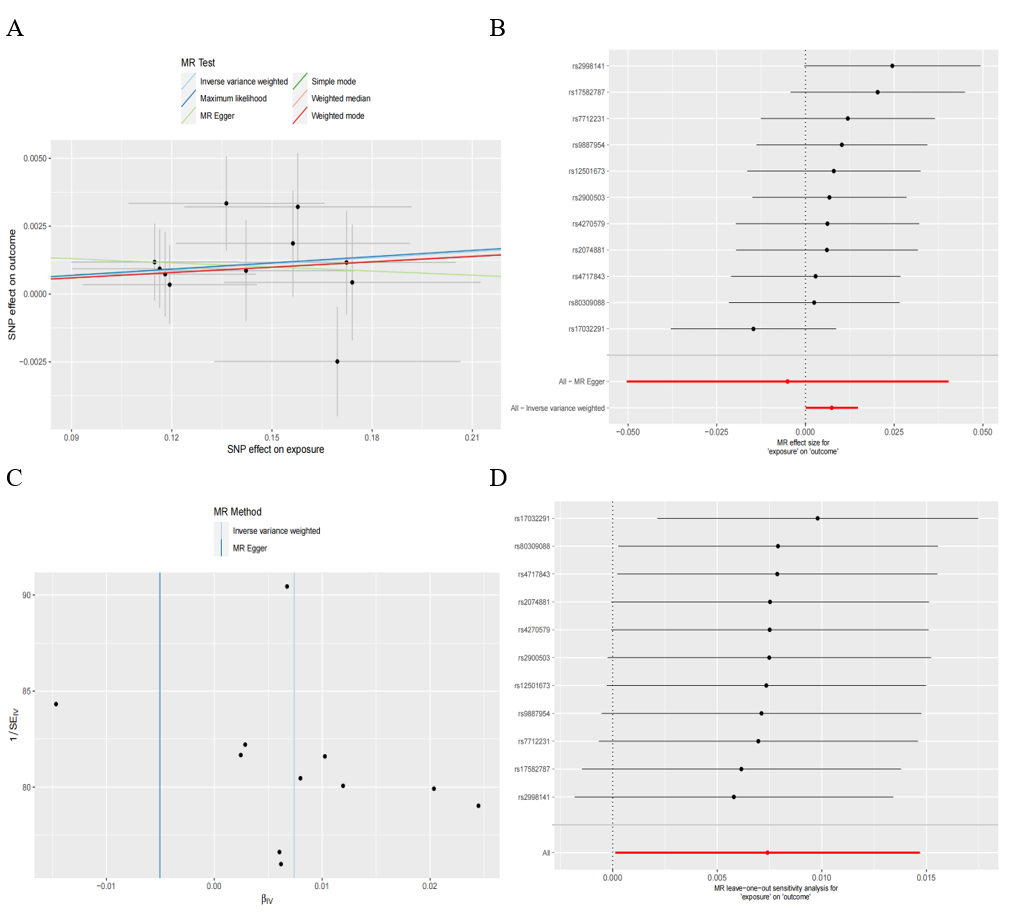
**

**Figure S42 Scatter plot (A), forest plot (B), funnel plot (C) and sensitivity analysis (D) of the causal effect of *Marvinbryantia* on insomnia.**

**
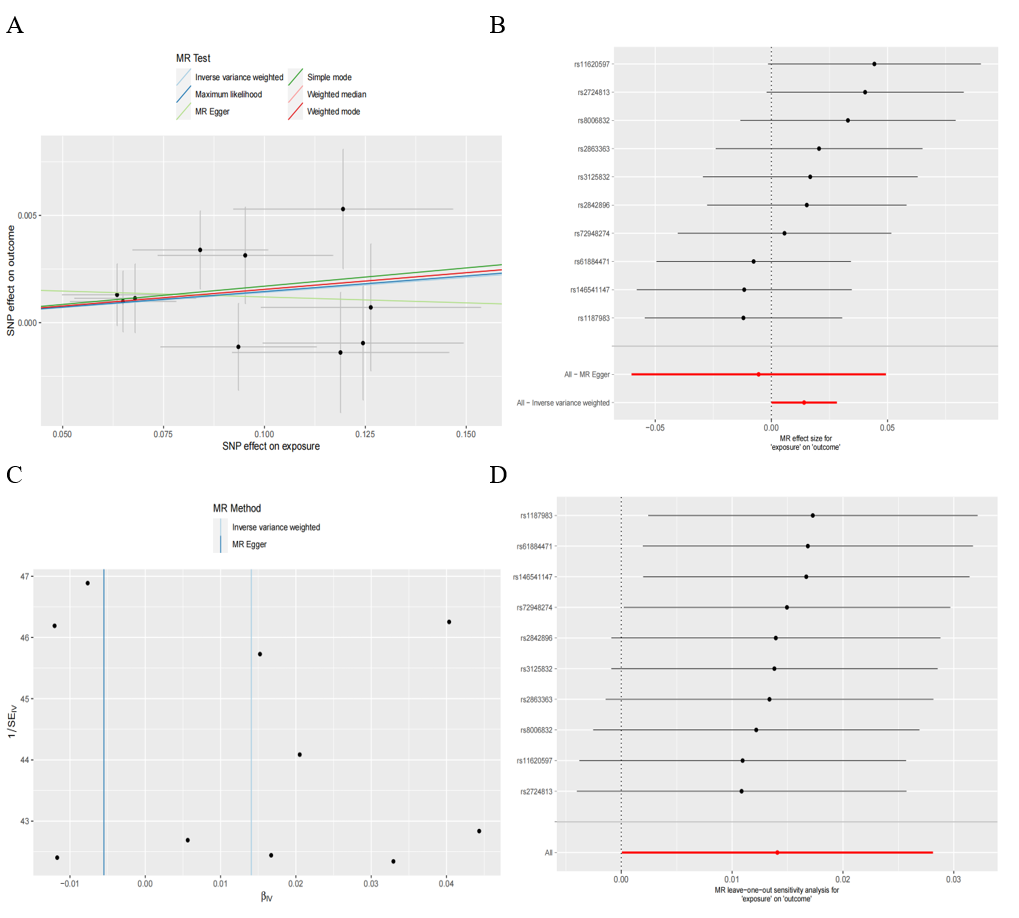
**

**Figure S43 Scatter plot (A), forest plot (B), funnel plot (C) and sensitivity analysis (D) of the causal effect of *Lachnospiraceae UCG004* on sleep duration.**

**
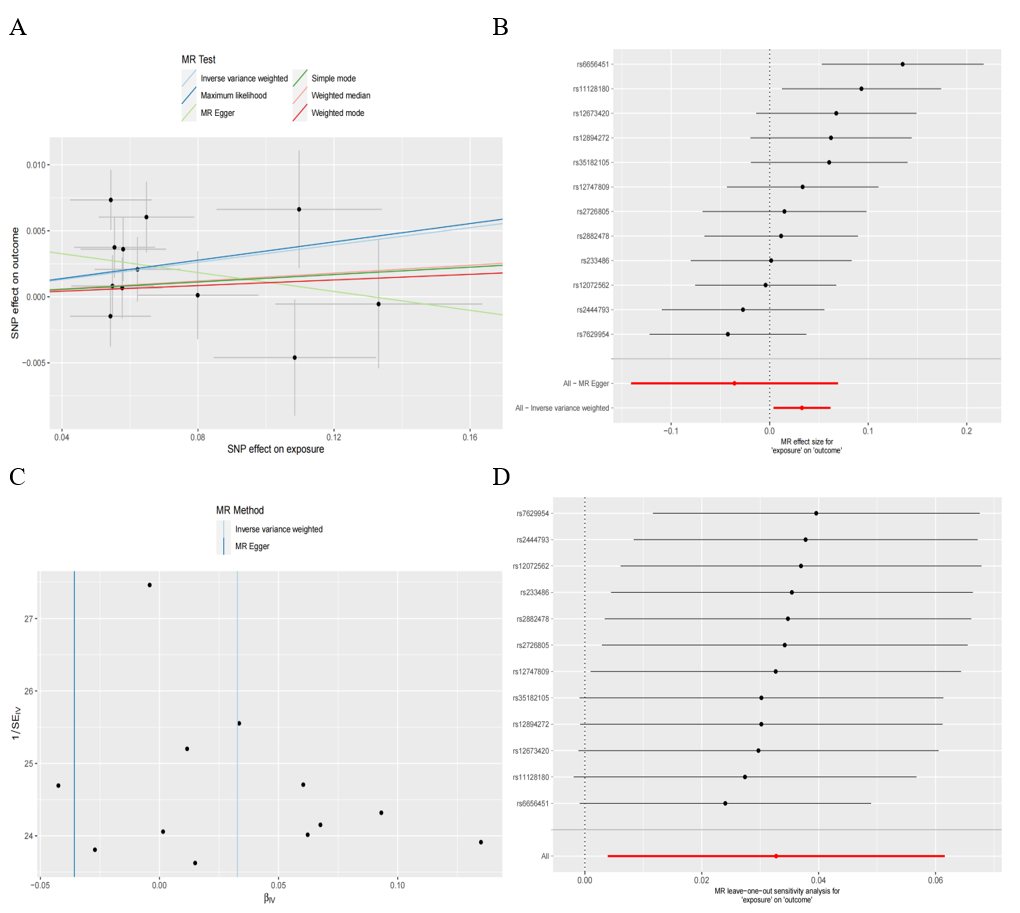
**

**Figure S44 Scatter plot (A), forest plot (B), funnel plot (C) and sensitivity analysis (D) of the causal effect of *Lachnospiraceae UCG004* on short sleep duration.**

**
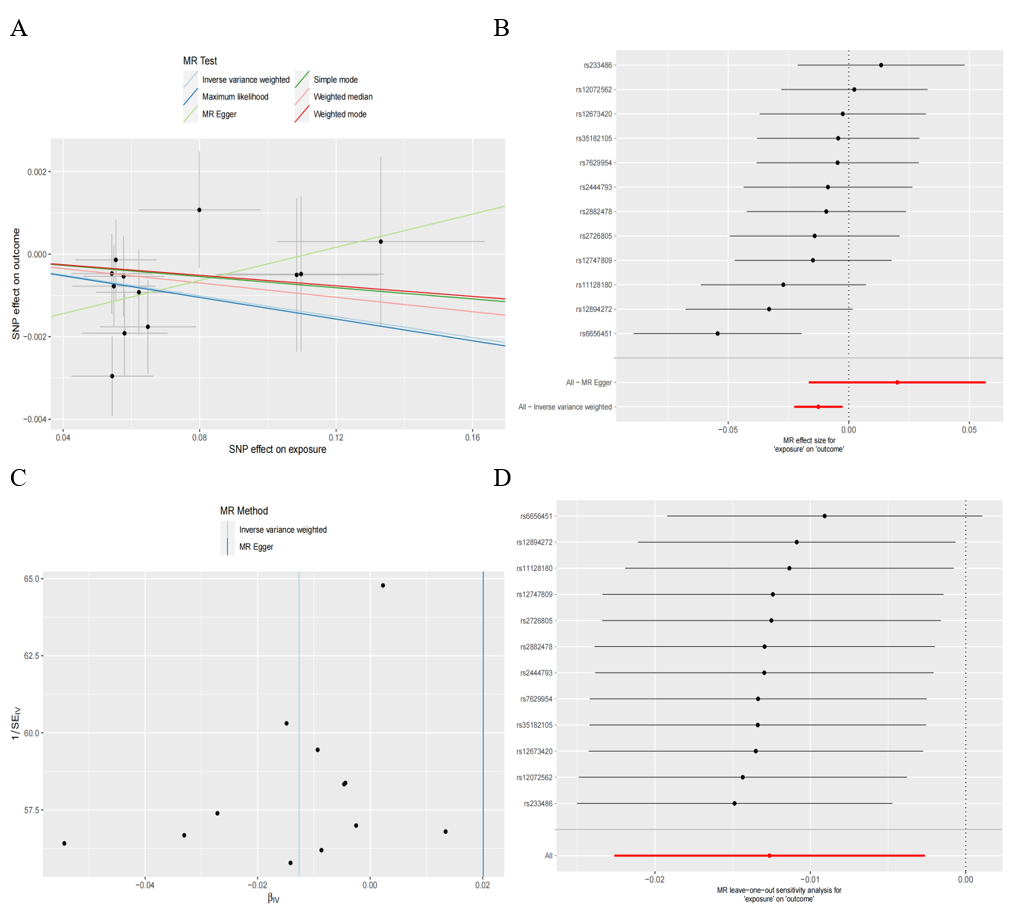
**

**Figure S45 Scatter plot (A), forest plot (B), funnel plot (C) and sensitivity analysis (D) of the causal effect of *Lachnospiraceae UCG010* on daytime napping.**

**
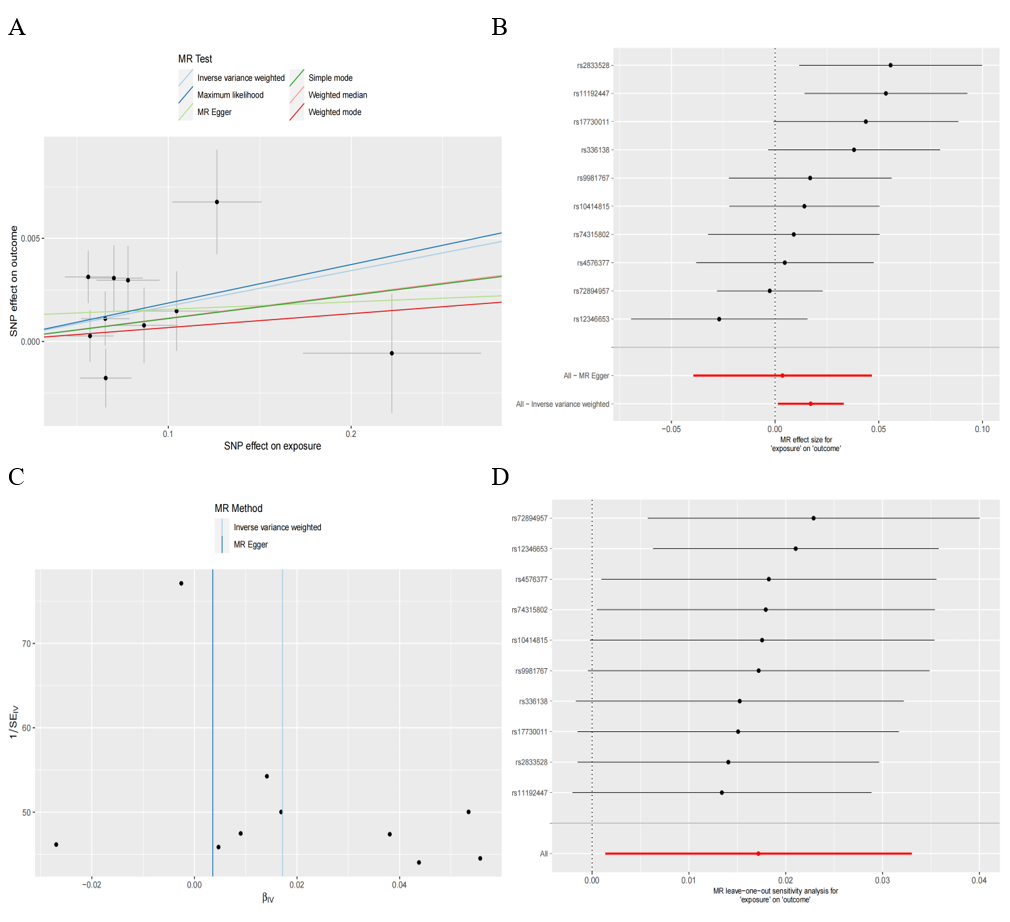
**

**Figure S46 Scatter plot (A), forest plot (B), funnel plot (C) and sensitivity analysis (D) of the causal effect of *Oscillibacter* on insomnia.**

**
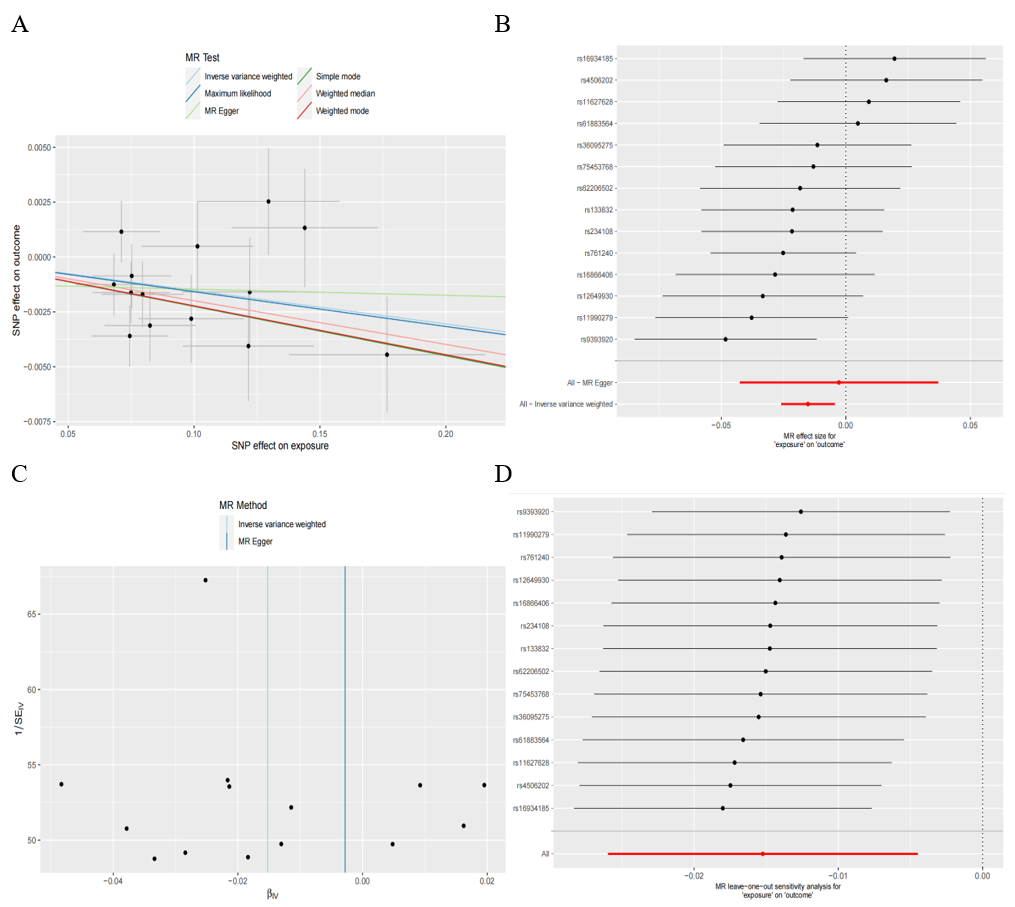
**

**Figure S47 Scatter plot (A), forest plot (B), funnel plot (C) and sensitivity analysis (D) of the causal effect of *Oscillibacter* on sleep duration.**

**
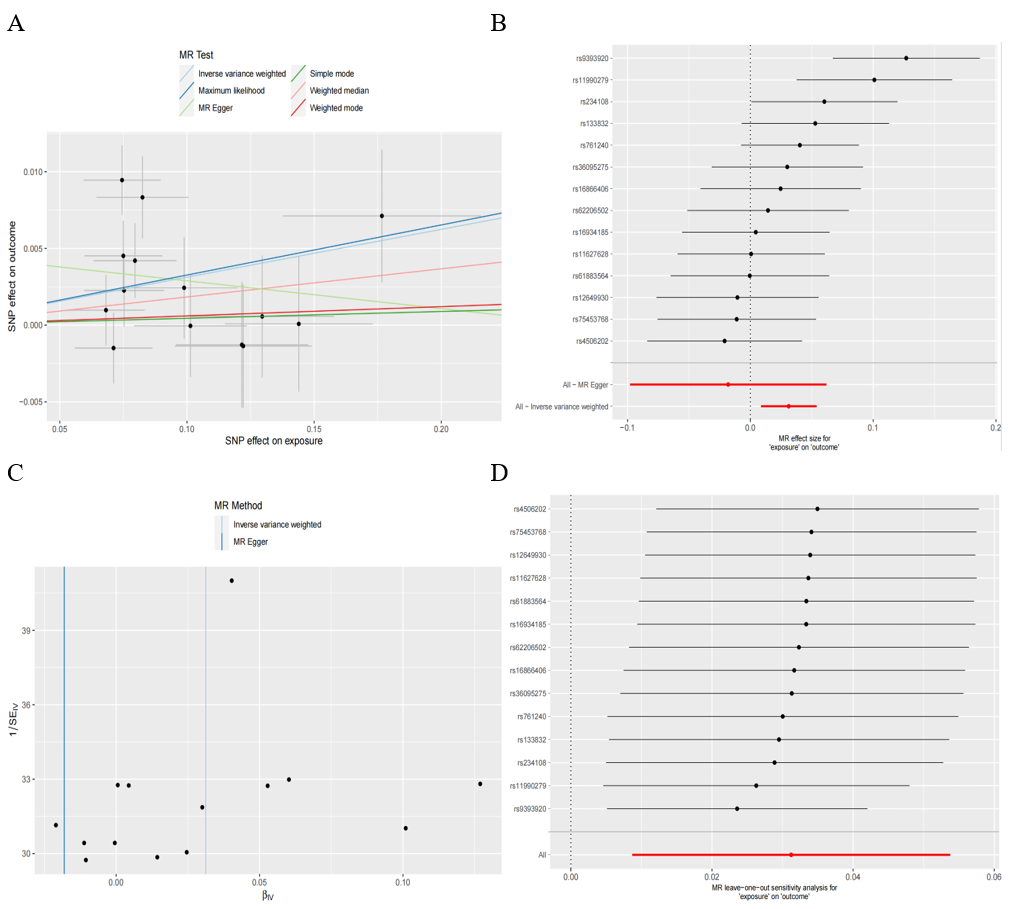
**

**Figure S48 Scatter plot (A), forest plot (B), funnel plot (C) and sensitivity analysis (D) of the causal effect of *Oscillibacter* on short sleep duration.**

**
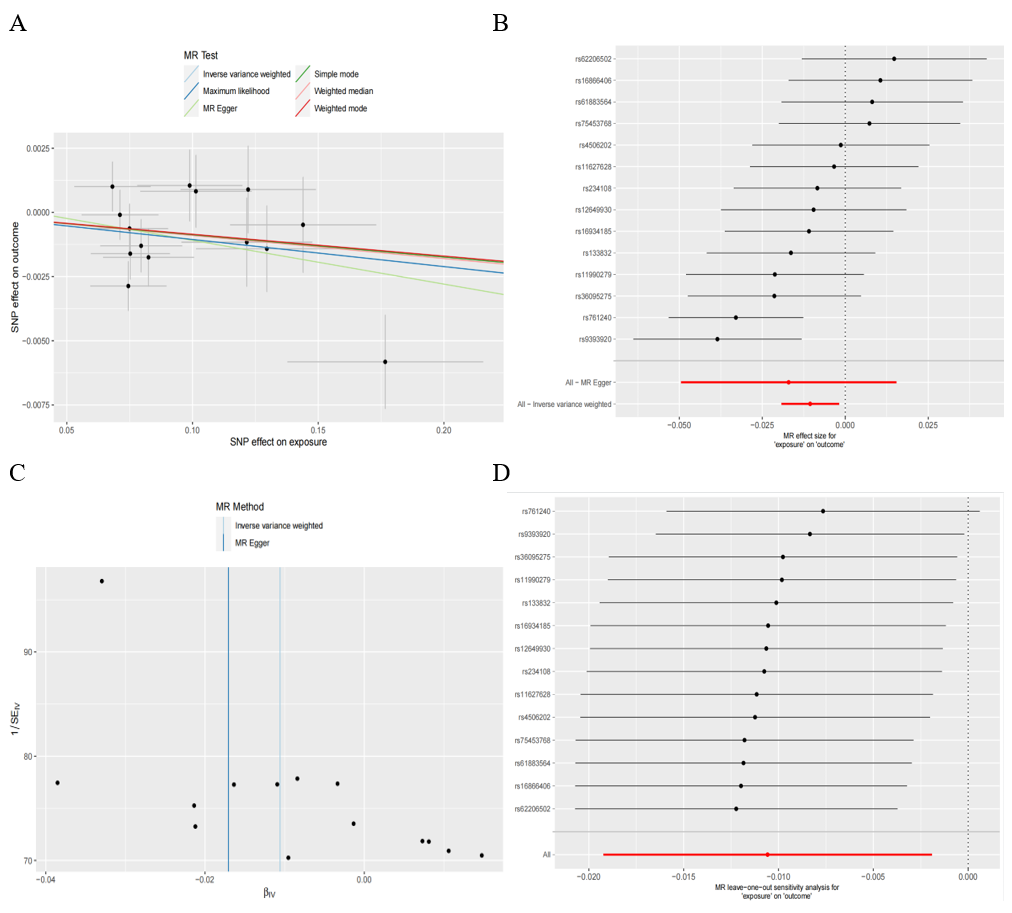
**

**Figure S49 Scatter plot (A), forest plot (B), funnel plot (C) and sensitivity analysis (D) of the causal effect of *Oxalobacter* on daytime napping.**

**
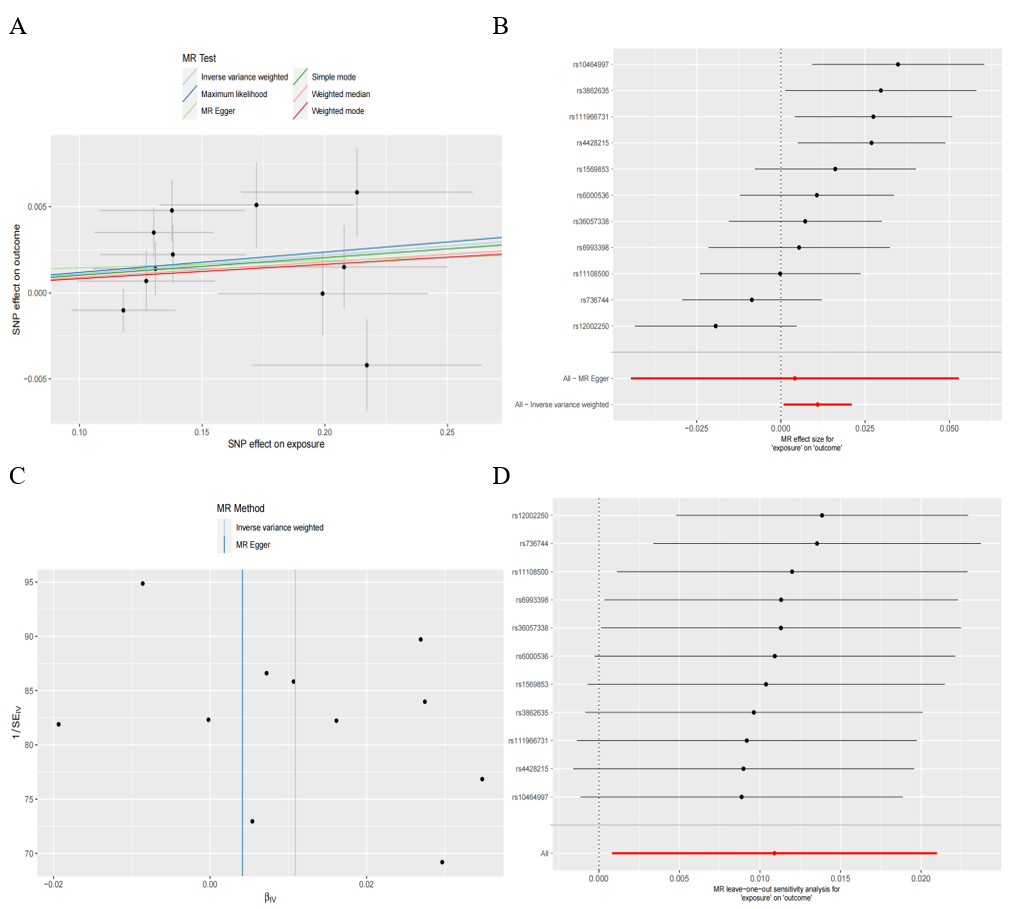
**

**Figure S50 Scatter plot (A), forest plot (B), funnel plot (C) and sensitivity analysis (D) of the causal effect of *Oxalobacter* on daytime sleepiness.**

**
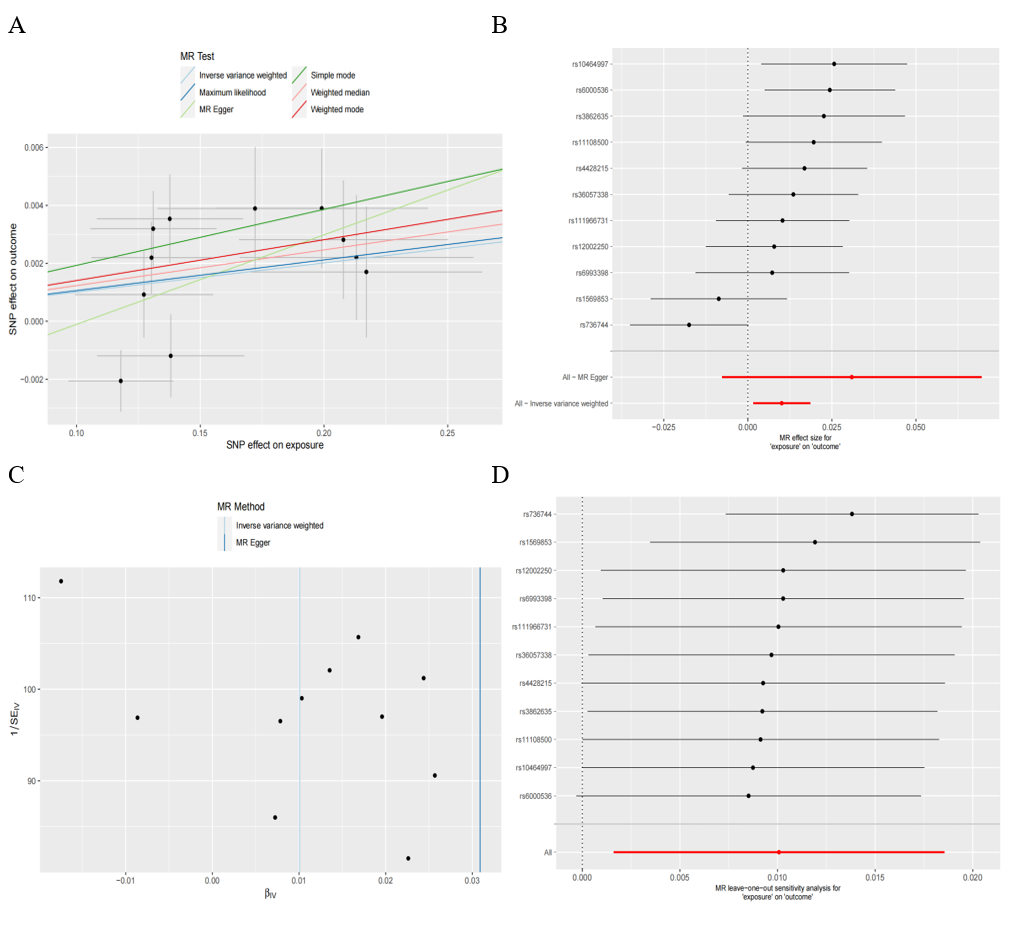
**

**Figure S51 Scatter plot (A), forest plot (B), funnel plot (C) and sensitivity analysis (D) of the causal effect of *Oxalobacter* on daytime sleepiness adjusted for BMI.**

**
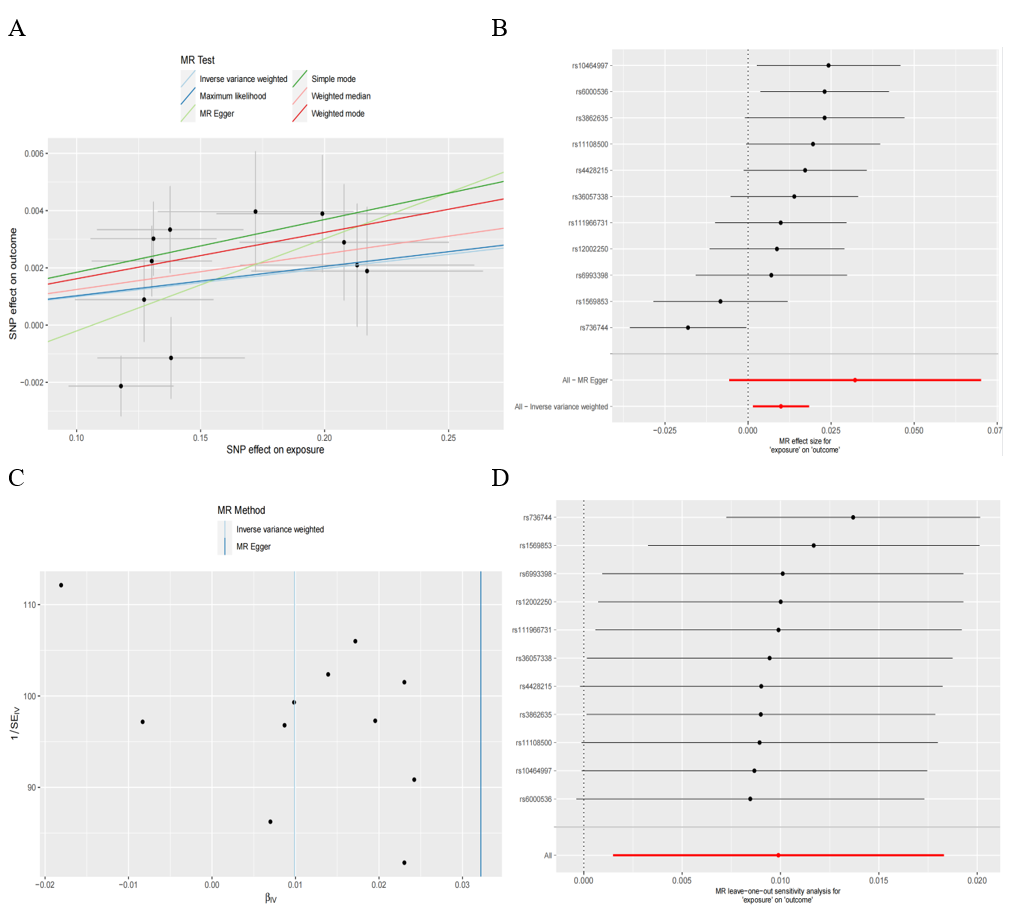
**

**Figure S52 Scatter plot (A), forest plot (B), funnel plot (C) and sensitivity analysis (D) of the causal effect of *Parabacteroides* on chronotype.**

**
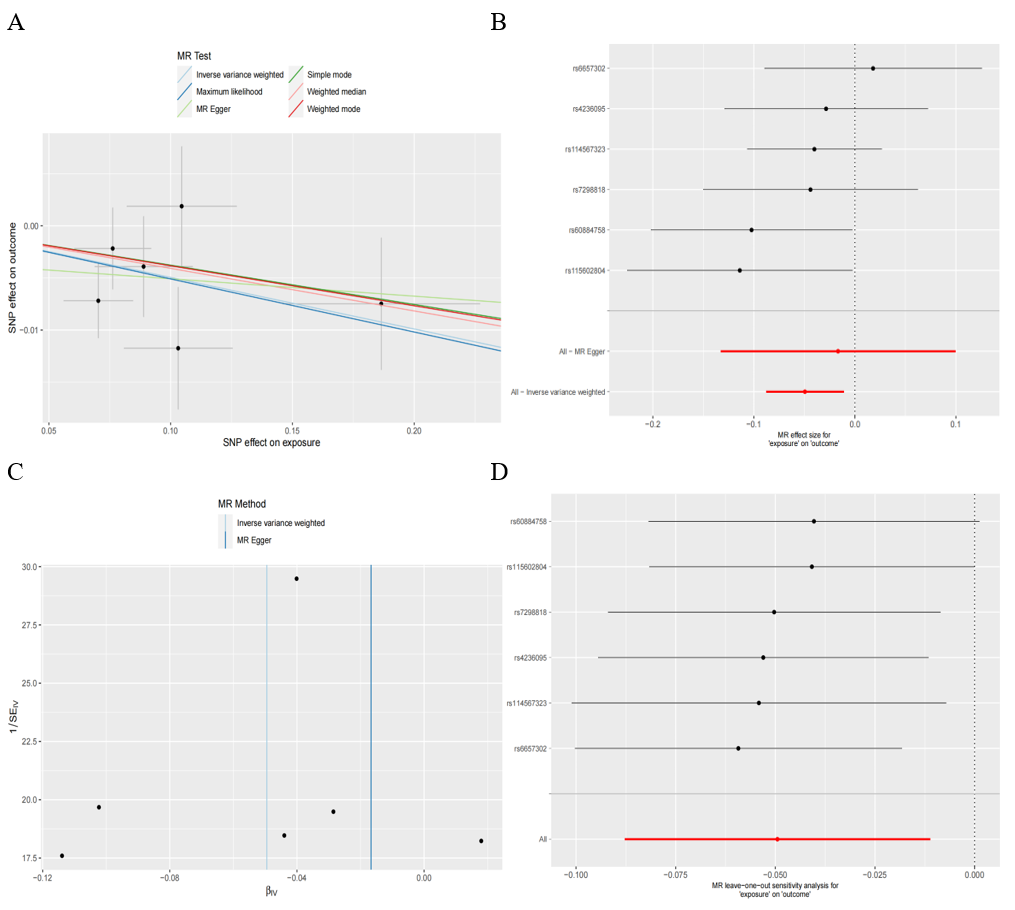
**

**Figure S53 Scatter plot (A), forest plot (B), funnel plot (C) and sensitivity analysis (D) of the causal effect of *Parabacteroides* on binary chronotype.**

**
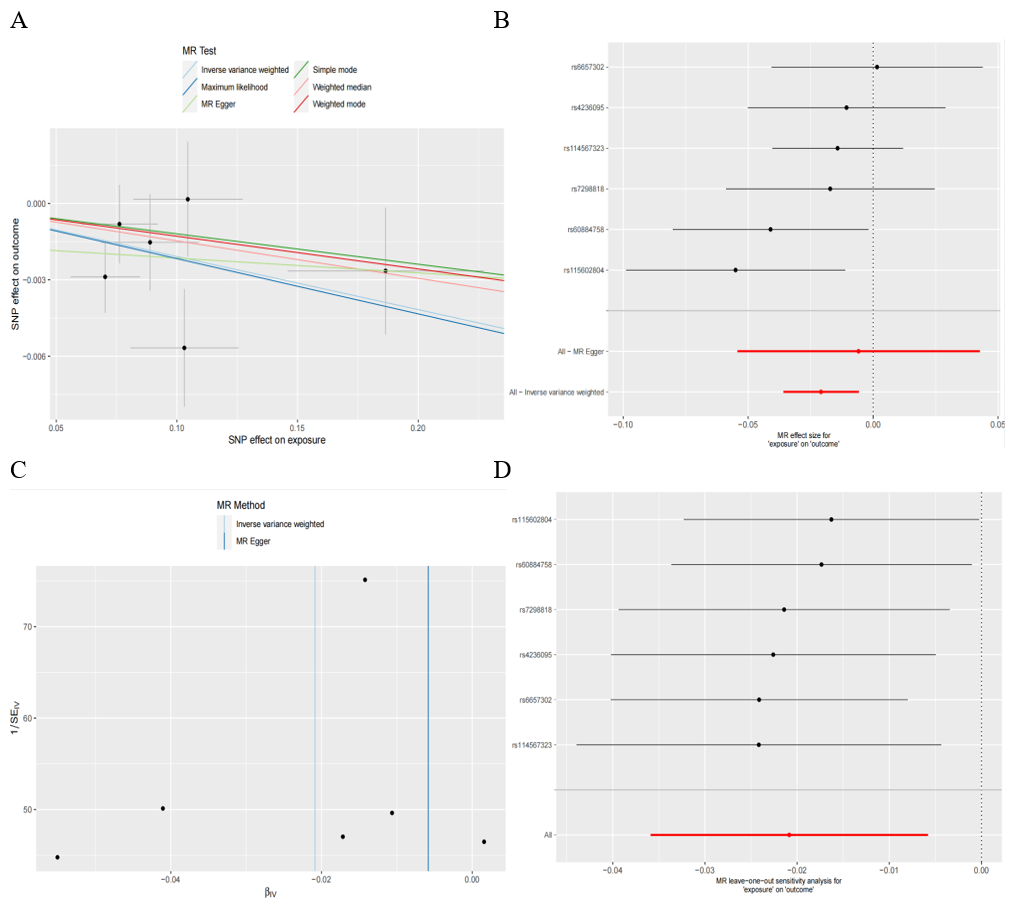
**

**Figure S54 Scatter plot (A), forest plot (B), funnel plot (C) and sensitivity analysis (D) of the causal effect of *Odoribacter* on insomnia.**

**
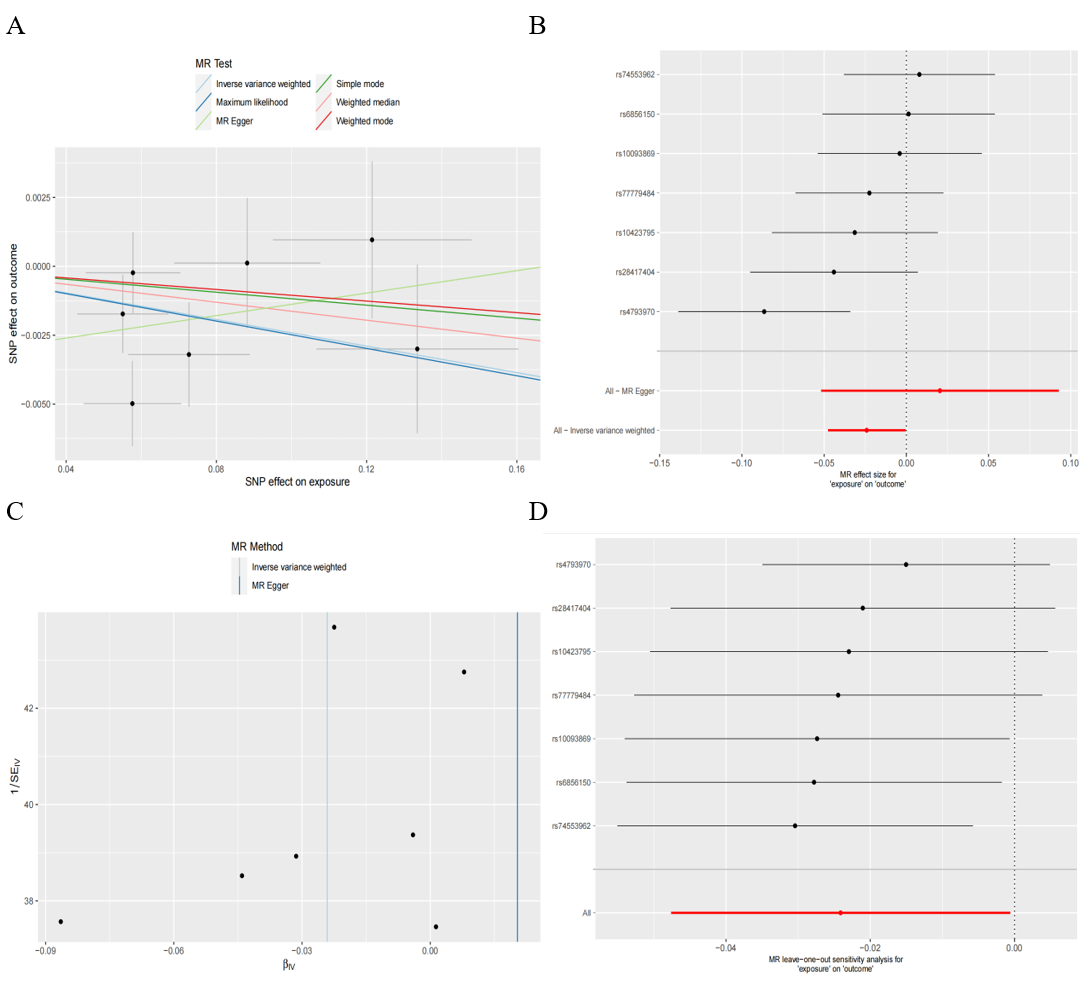
**

**Figure S55 Scatter plot (A), forest plot (B), funnel plot (C) and sensitivity analysis (D) of the causal effect of *Odoribacter* on sleep duration.**

**
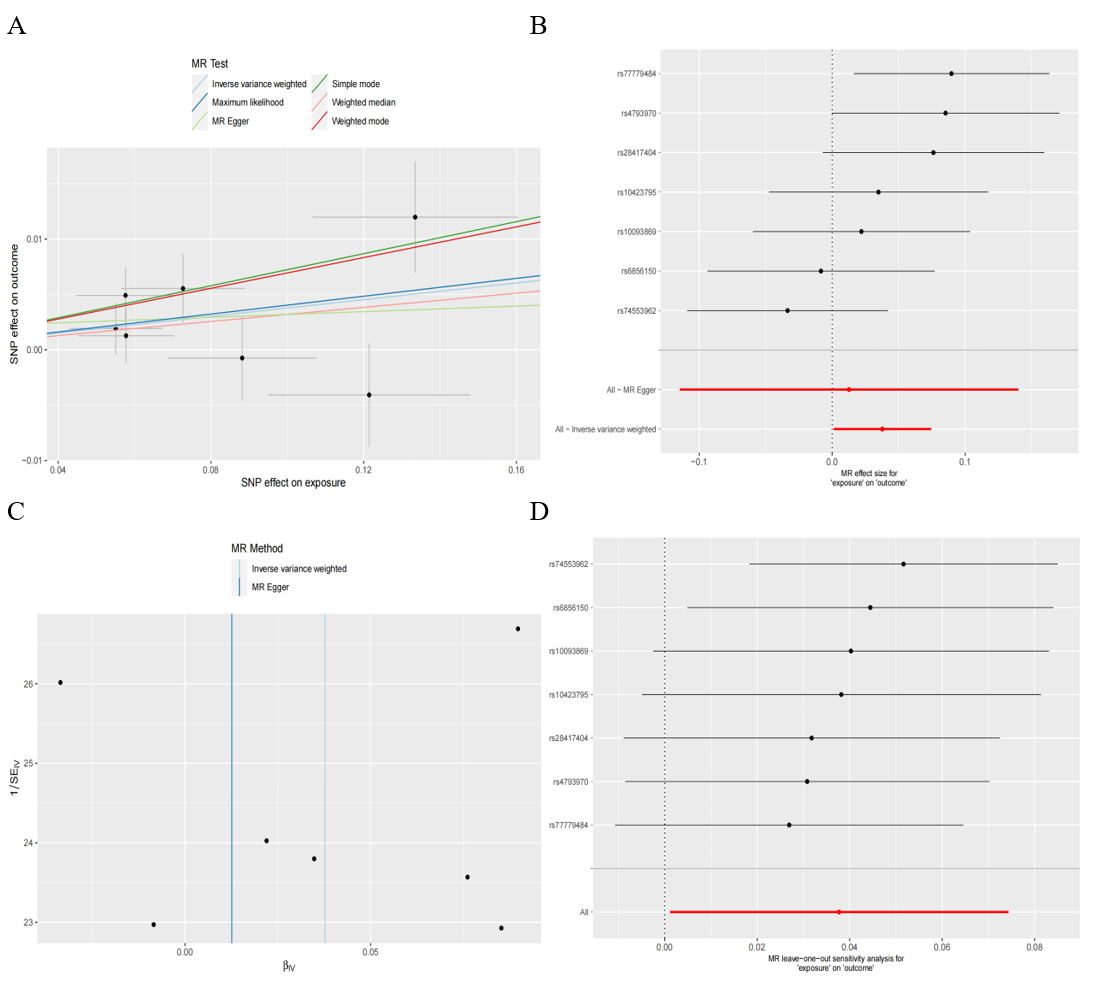
**

**Figure S56 Scatter plot (A), forest plot (B), funnel plot (C) and sensitivity analysis (D) of the causal effect of *Prevotella 7* on insomnia.**

**
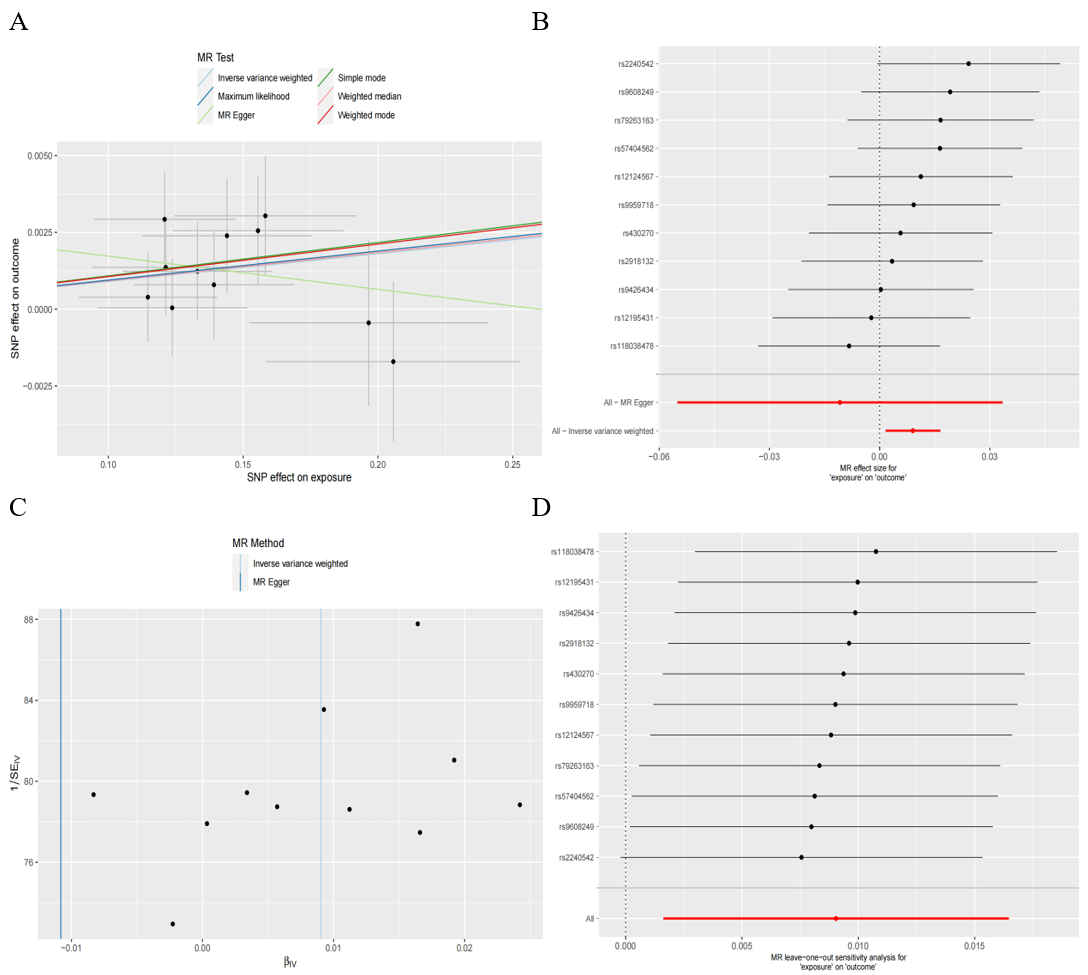
**

**Figure S57 Scatter plot (A), forest plot (B), funnel plot (C) and sensitivity analysis (D) of the causal effect of *Prevotella 7* on chronotype.**

**
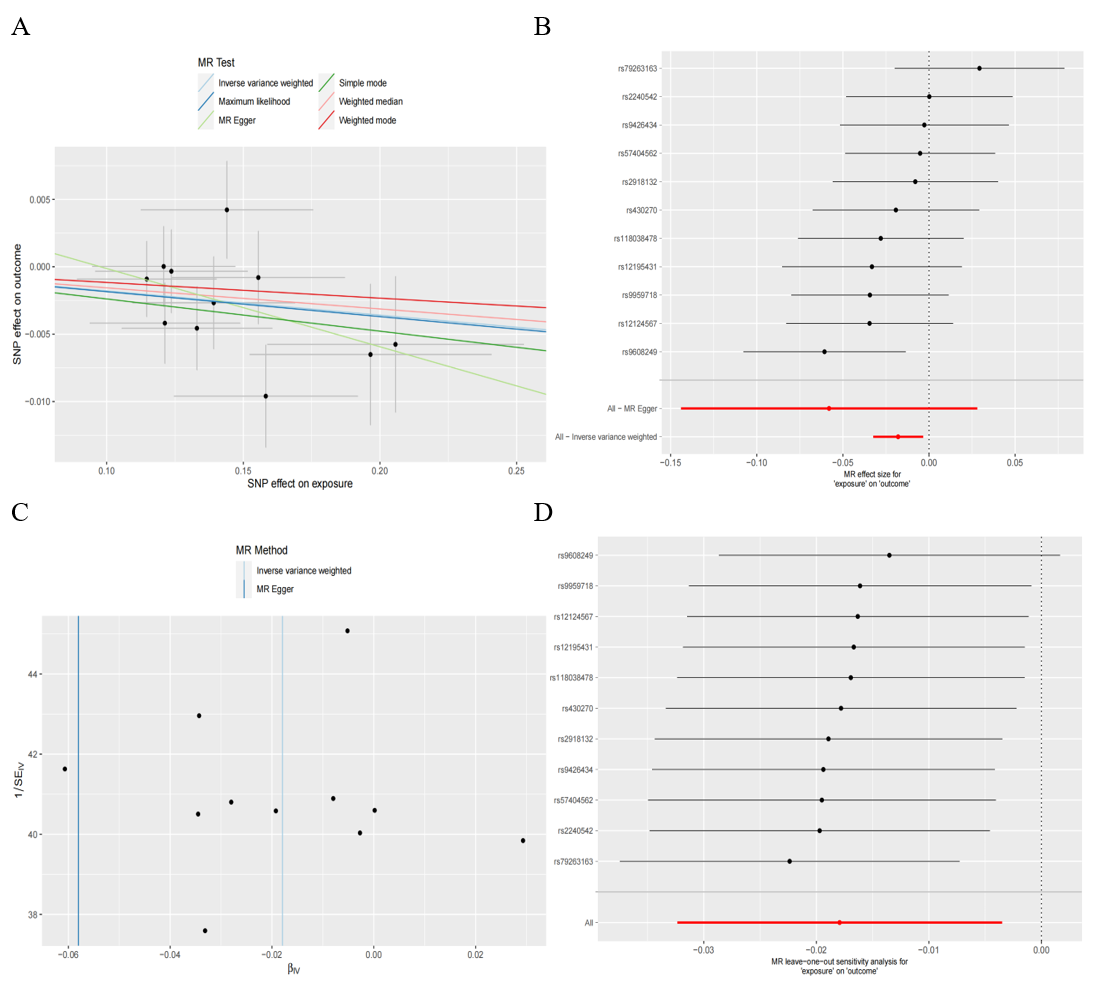
**

**Figure S58 Scatter plot (A), forest plot (B), funnel plot (C) and sensitivity analysis (D) of the causal effect of *Prevotella 7* on binary chronotype.**

**
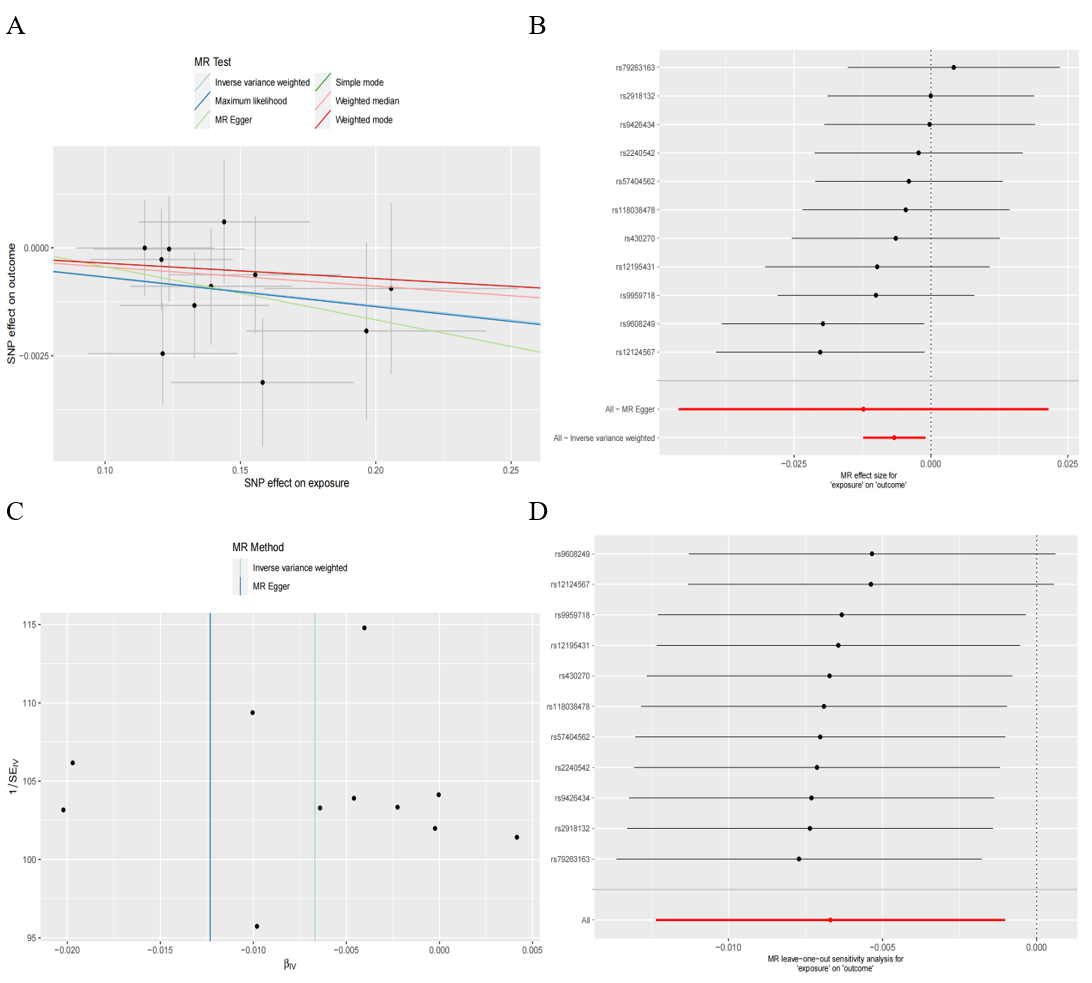
**

**Figure S59 Scatter plot (A), forest plot (B), funnel plot (C) and sensitivity analysis (D) of the causal effect of *Peptococcus* on daytime sleepiness.**

**
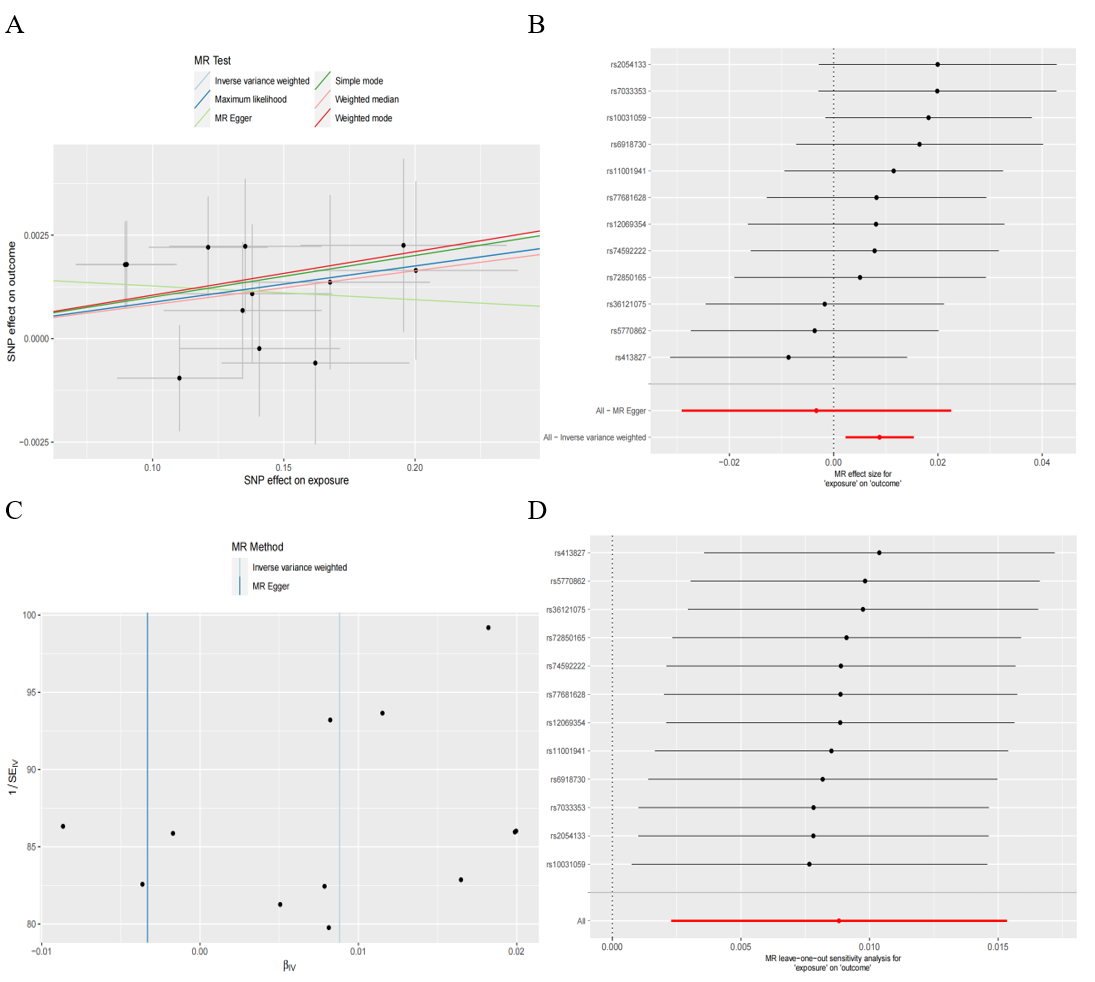
**

**Figure S60 Scatter plot (A), forest plot (B), funnel plot (C) and sensitivity analysis (D) of the causal effect of *Peptococcus* on daytime sleepiness adjusted for BMI.**

**
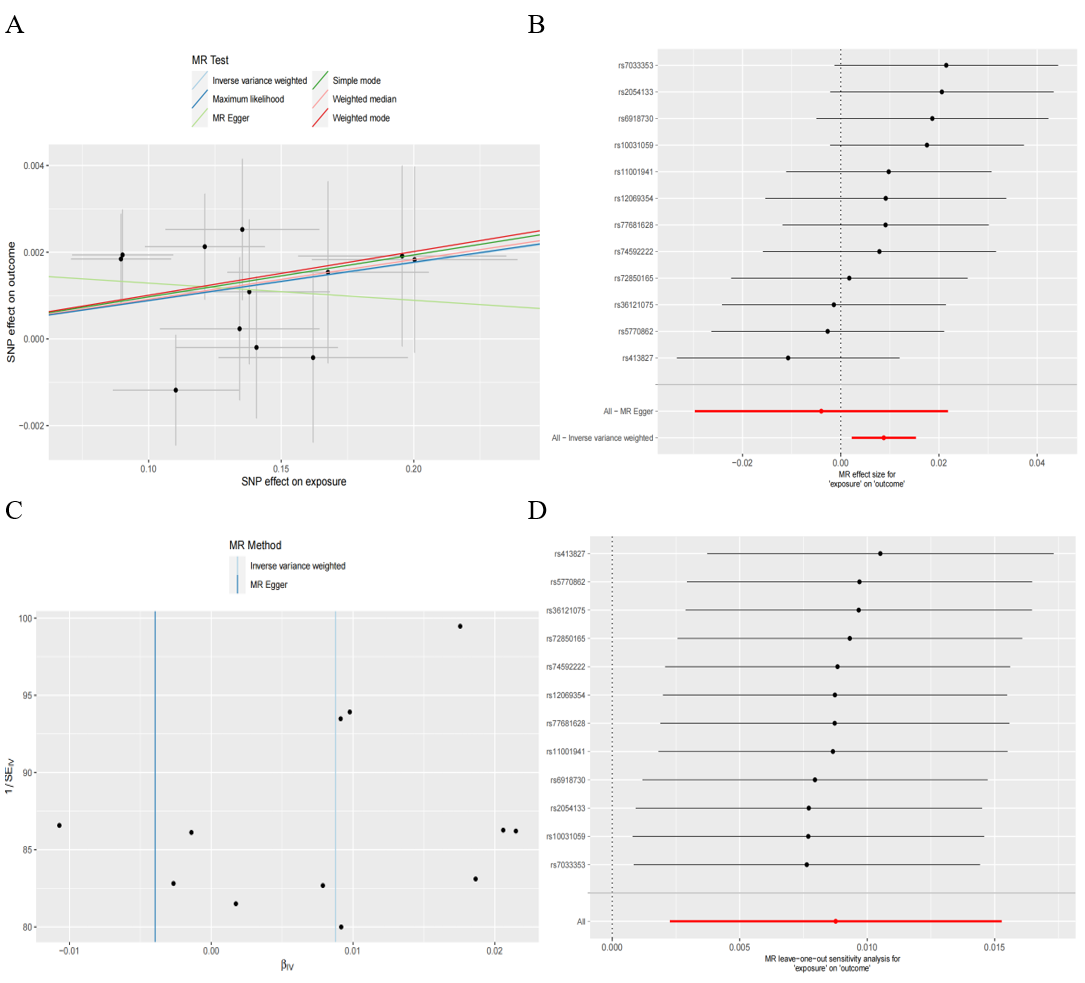
**

**Figure S61 Scatter plot (A), forest plot (B), funnel plot (C) and sensitivity analysis (D) of the causal effect of *Ruminiclostridium 6* on long sleep duration.**

**
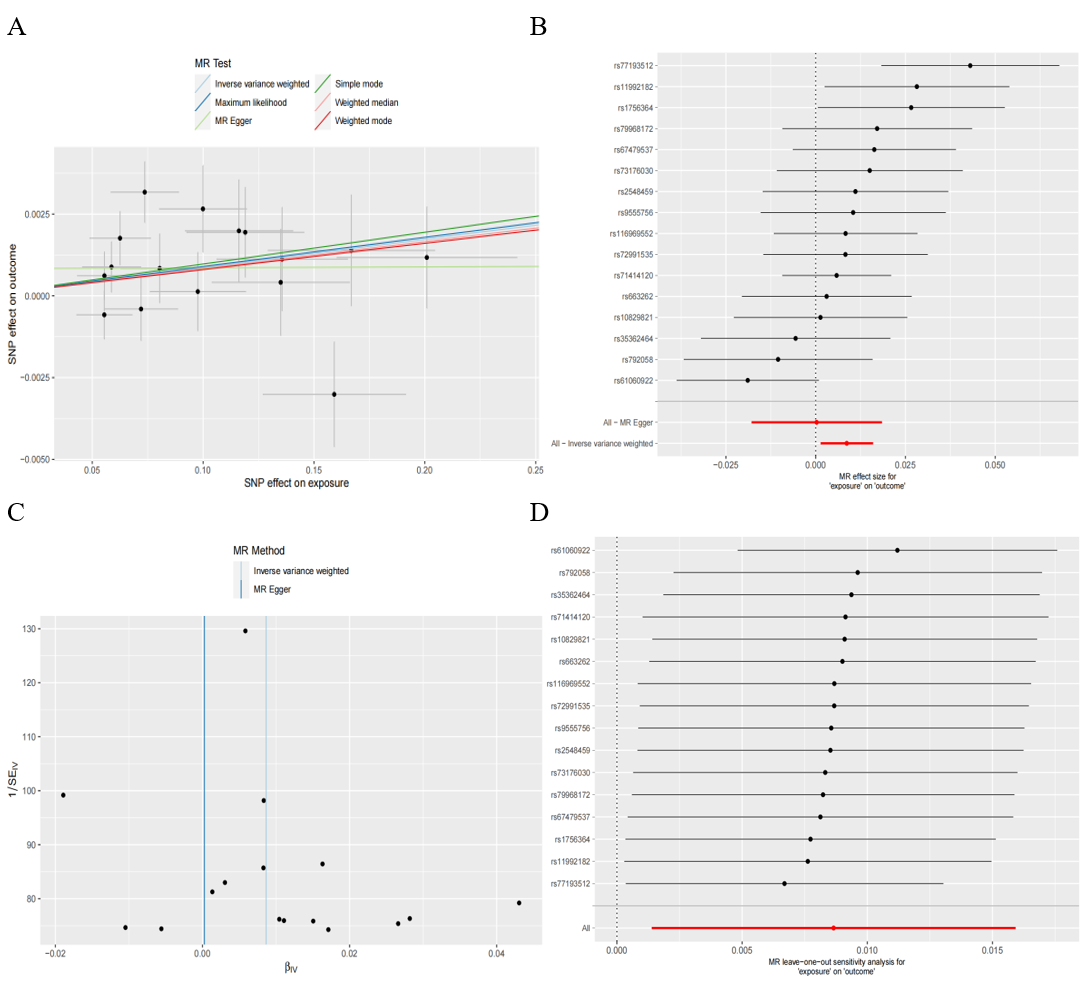
**

**Figure S62 Scatter plot (A), forest plot (B), funnel plot (C) and sensitivity analysis (D) of the causal effect of *Ruminococcaceae NK4A214 group* on long sleep duration.**

**
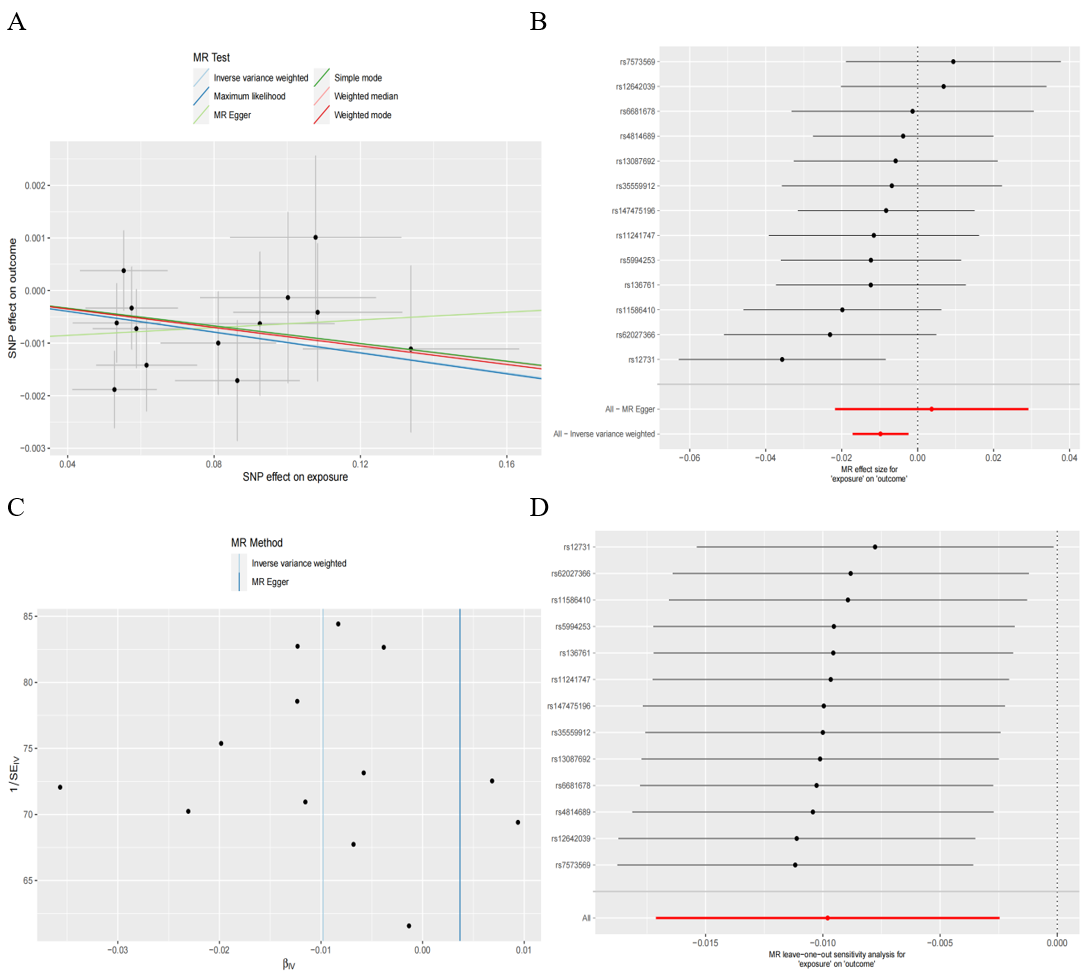
**

**Figure S63 Scatter plot (A), forest plot (B), funnel plot (C) and sensitivity analysis (D) of the causal effect of *Ruminococcaceae UCG002* on daytime napping.**

**
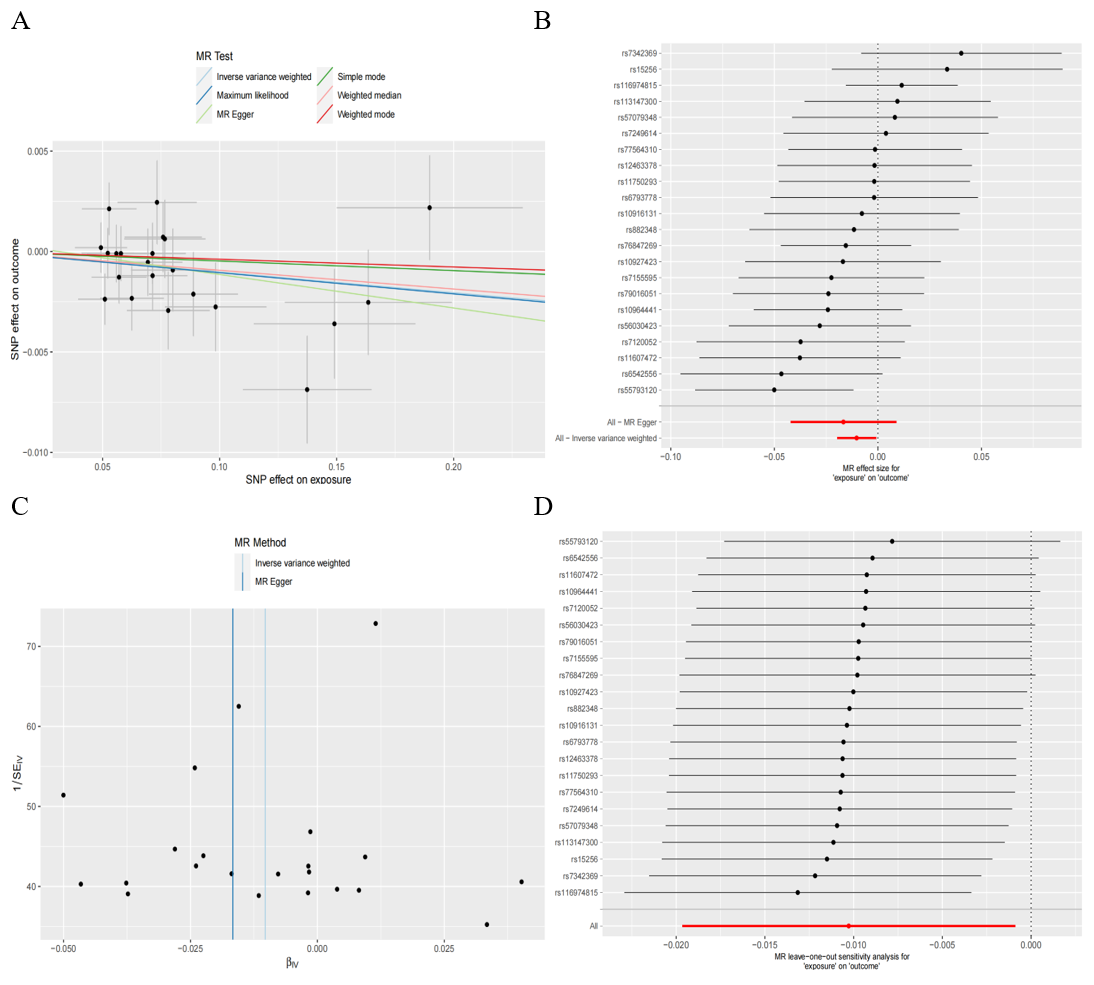
**

**Figure S64 Scatter plot (A), forest plot (B), funnel plot (C) and sensitivity analysis (D) of the causal effect of *Intestinibacter* on chronotype.**

**
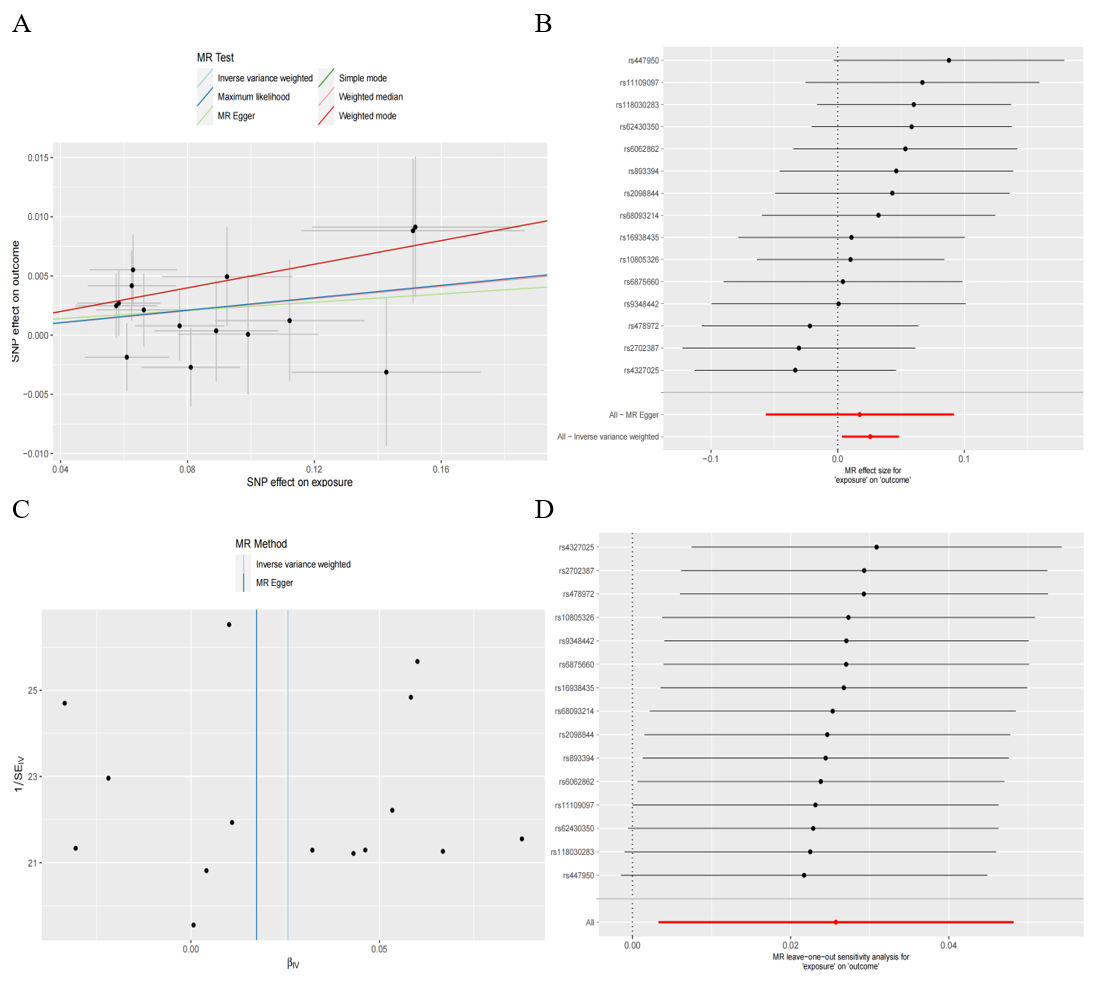
**

**Figure S65 Scatter plot (A), forest plot (B), funnel plot (C) and sensitivity analysis (D) of the causal effect of *Intestinibacter* on binary chronotype.**

**
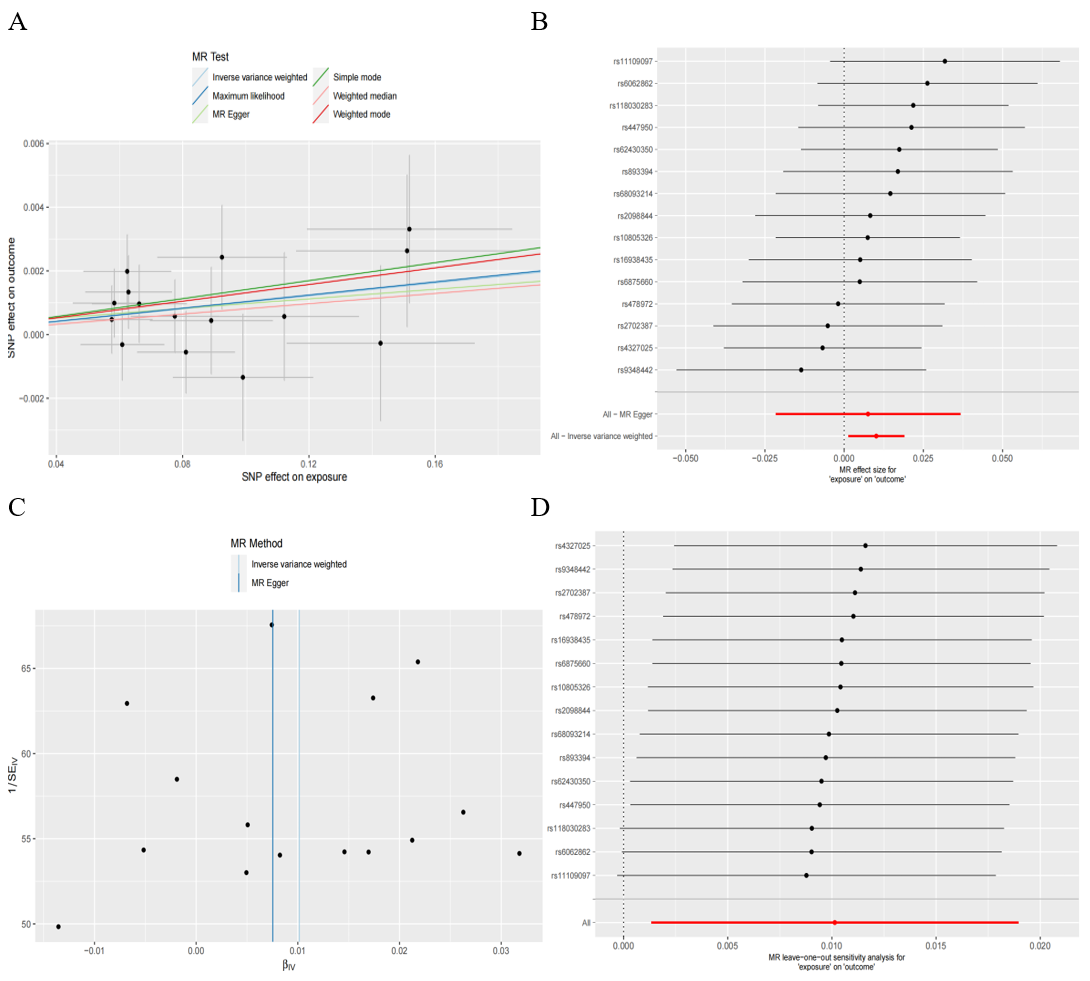
**

**Figure S66 Scatter plot (A), forest plot (B), funnel plot (C) and sensitivity analysis (D) of the causal effect of *Lachnoclostridium* on insomnia.**

**
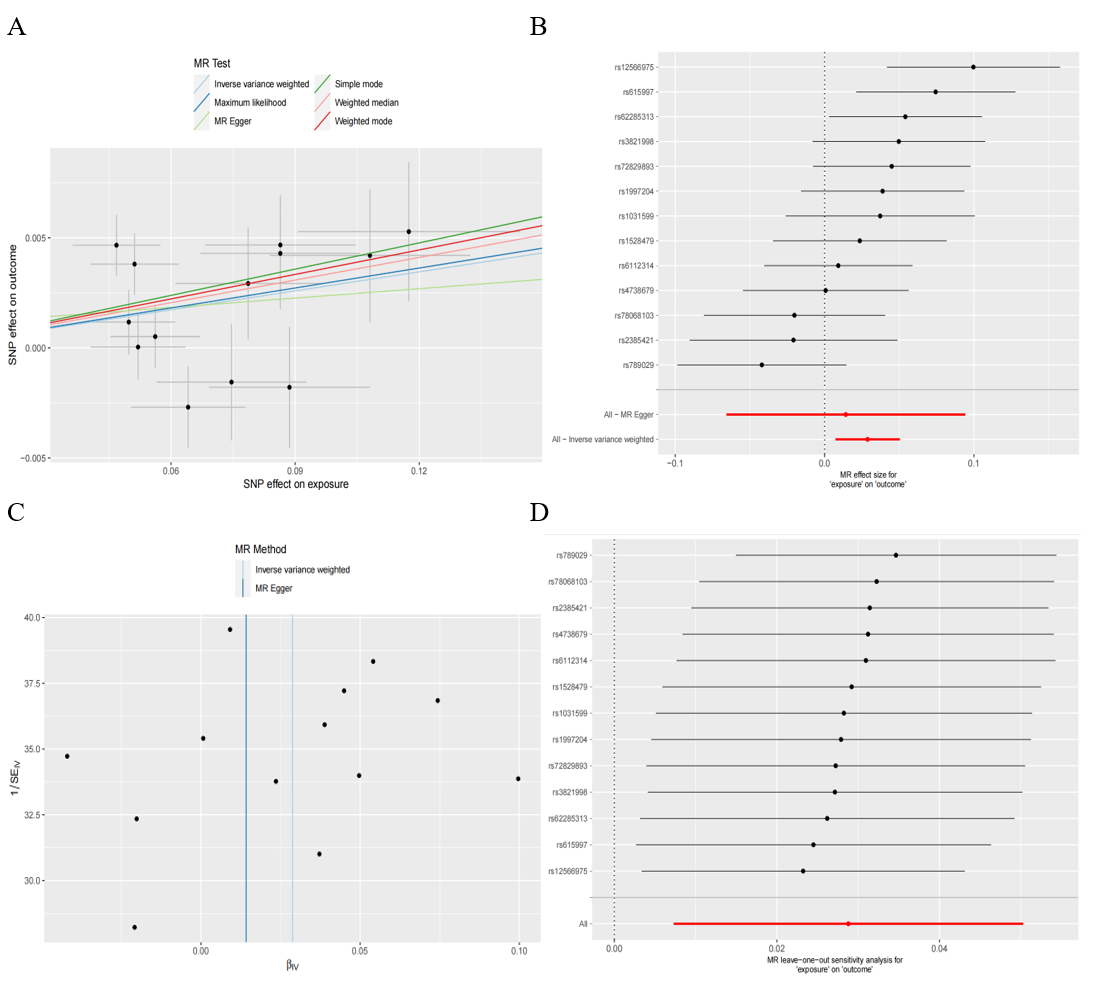
**

**Figure S67 Scatter plot (A), forest plot (B), funnel plot (C) and sensitivity analysis (D) of the causal effect of *Ruminococcus 1* on binary chronotype.**

**
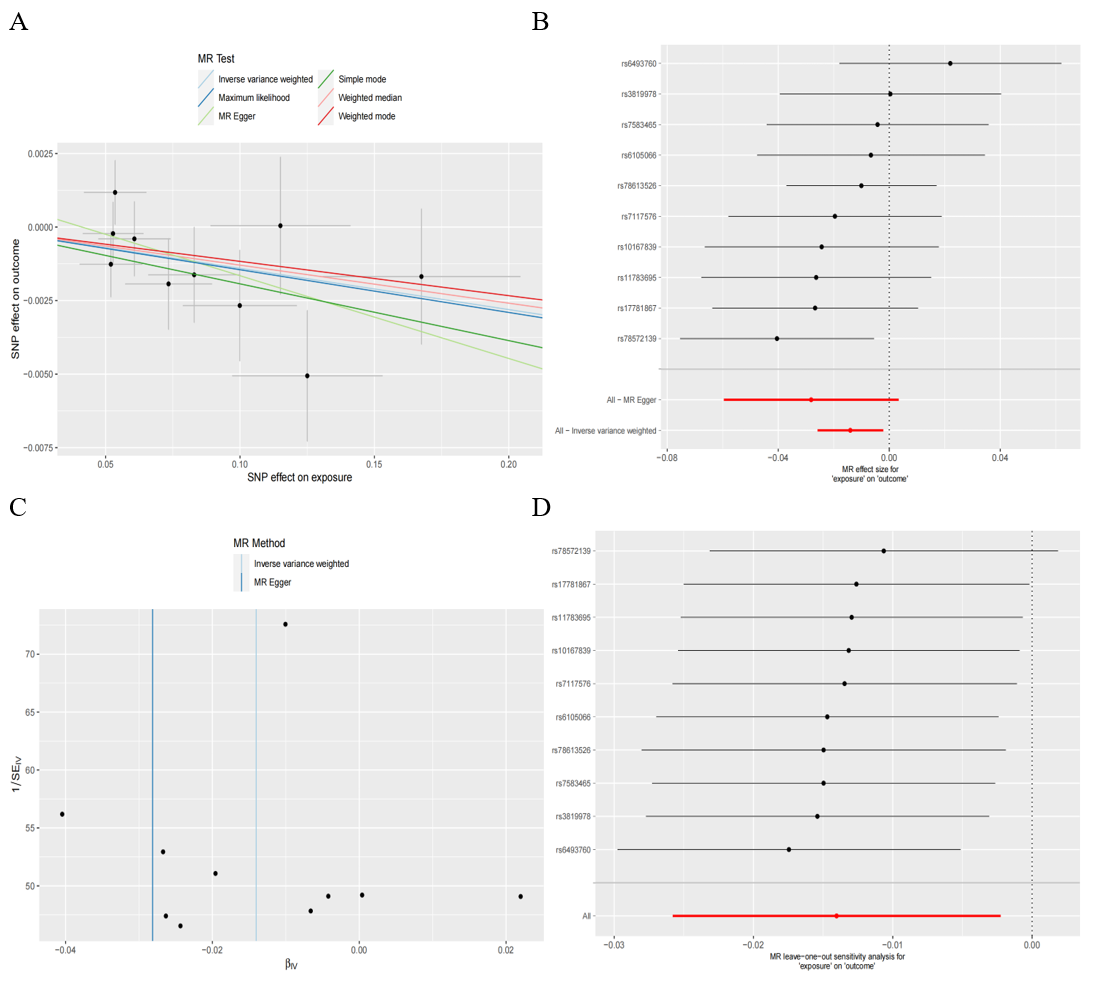
**

**Figure S68 Scatter plot (A), forest plot (B), funnel plot (C) and sensitivity analysis (D) of the causal effect of *Slackia* on daytime sleepiness.**

**
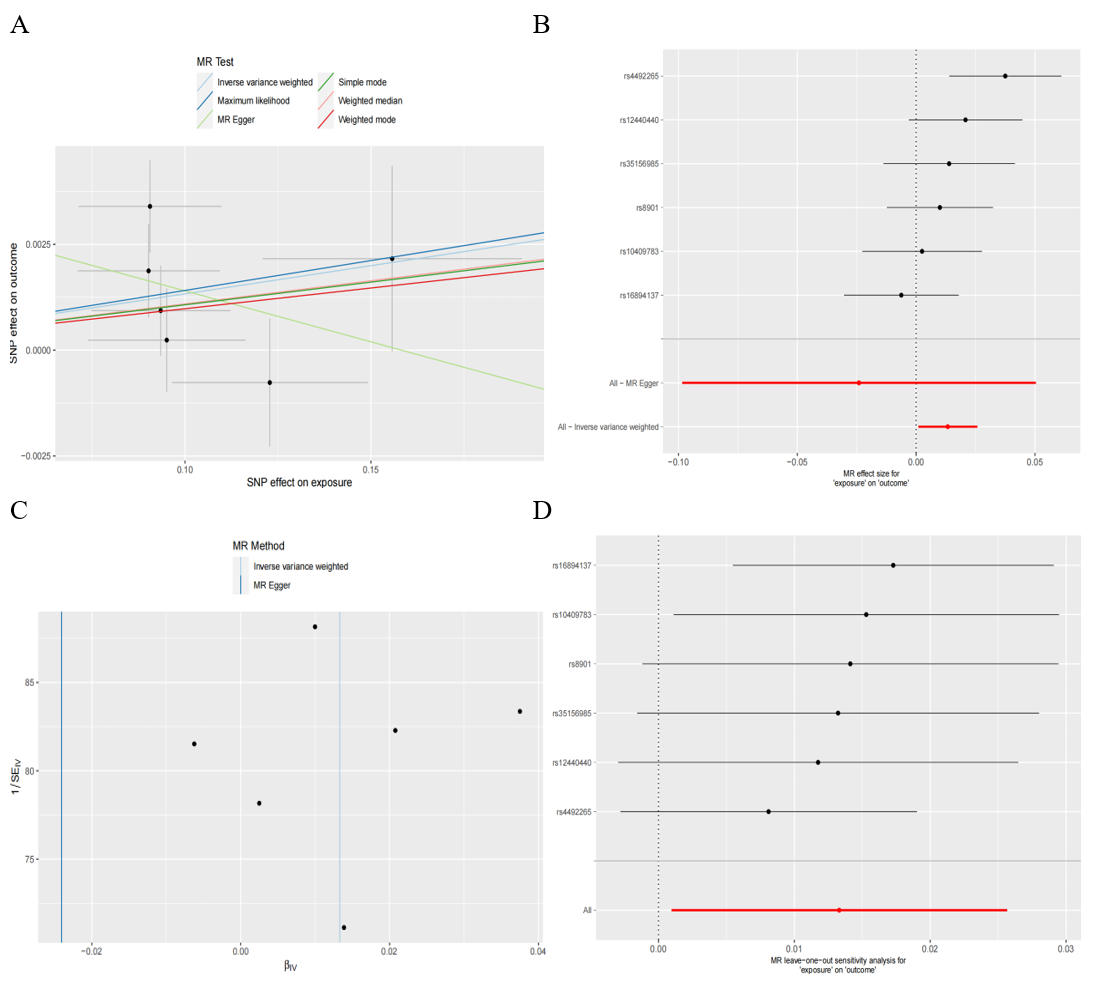
**

**Figure S69 Scatter plot (A), forest plot (B), funnel plot (C) and sensitivity analysis (D) of the causal effect of *Slackia* on daytime sleepiness adjusted for BMI.**

**
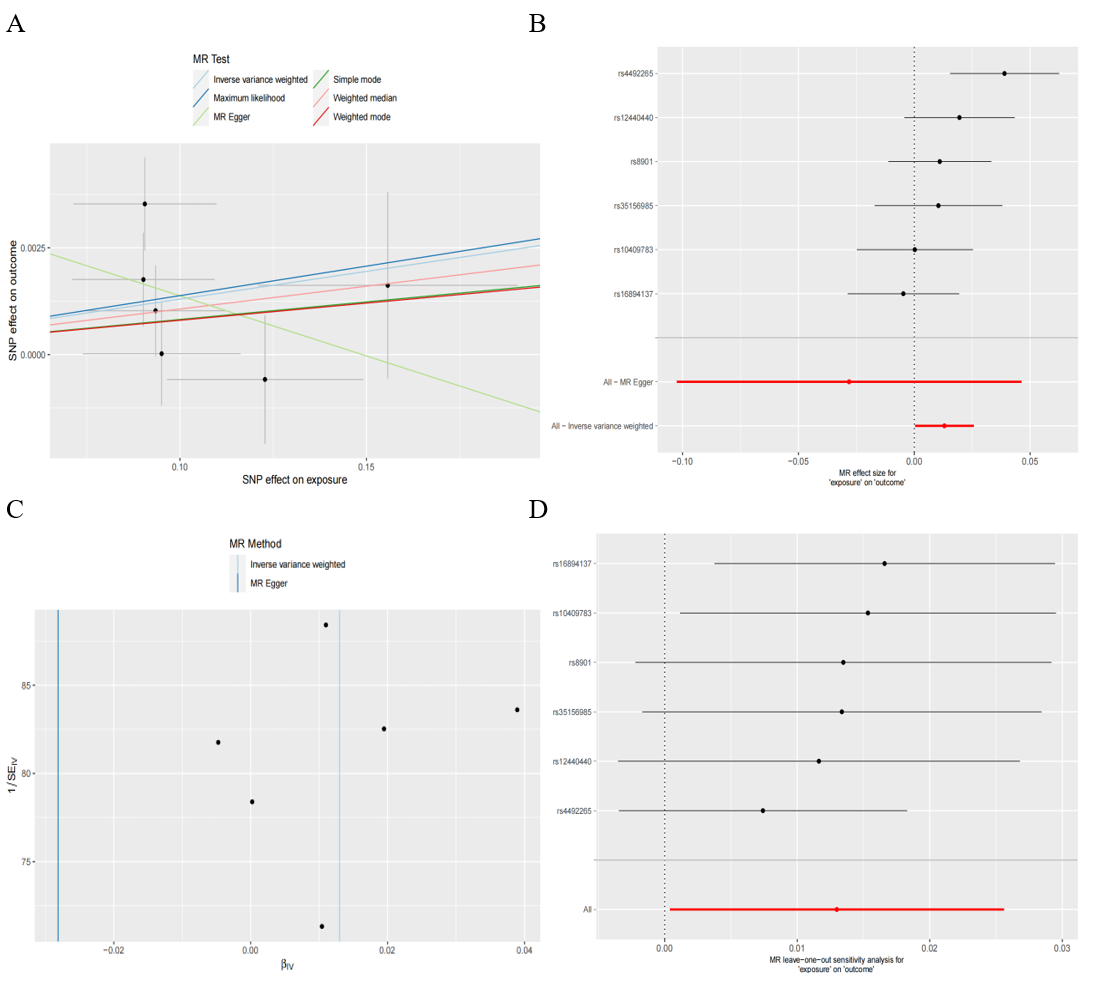
**

**Figure S70 Scatter plot (A), forest plot (B), funnel plot (C) and sensitivity analysis (D) of the causal effect of *Slackia* on long sleep duration.**

**
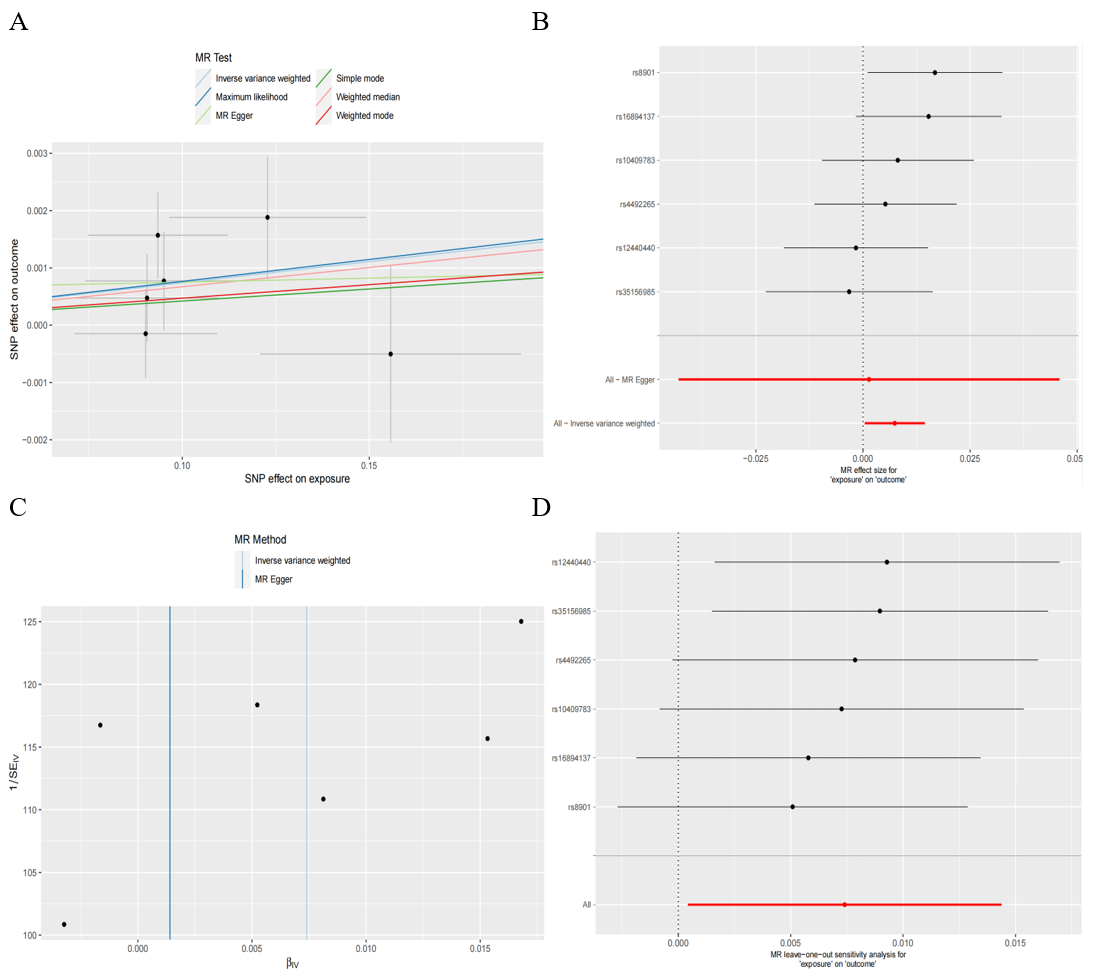
**

**Figure S71 Scatter plot (A), forest plot (B), funnel plot (C) and sensitivity analysis (D) of the causal effect of *Ruminococcaceae UCG013* on daytime napping.**

**
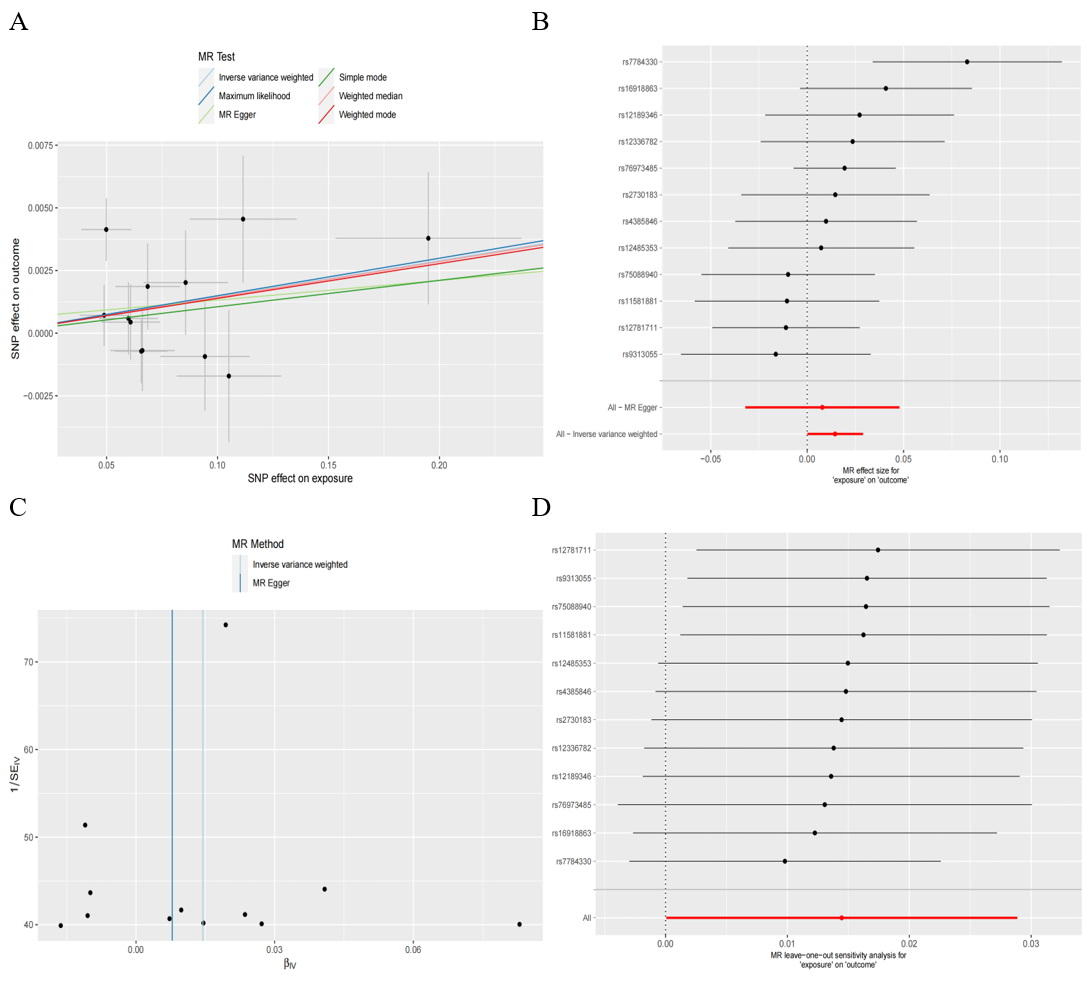
**

**Figure S72 Scatter plot (A), forest plot (B), funnel plot (C) and sensitivity analysis (D) of the causal effect of *Tyzzerella 3* on chronotype.**

**
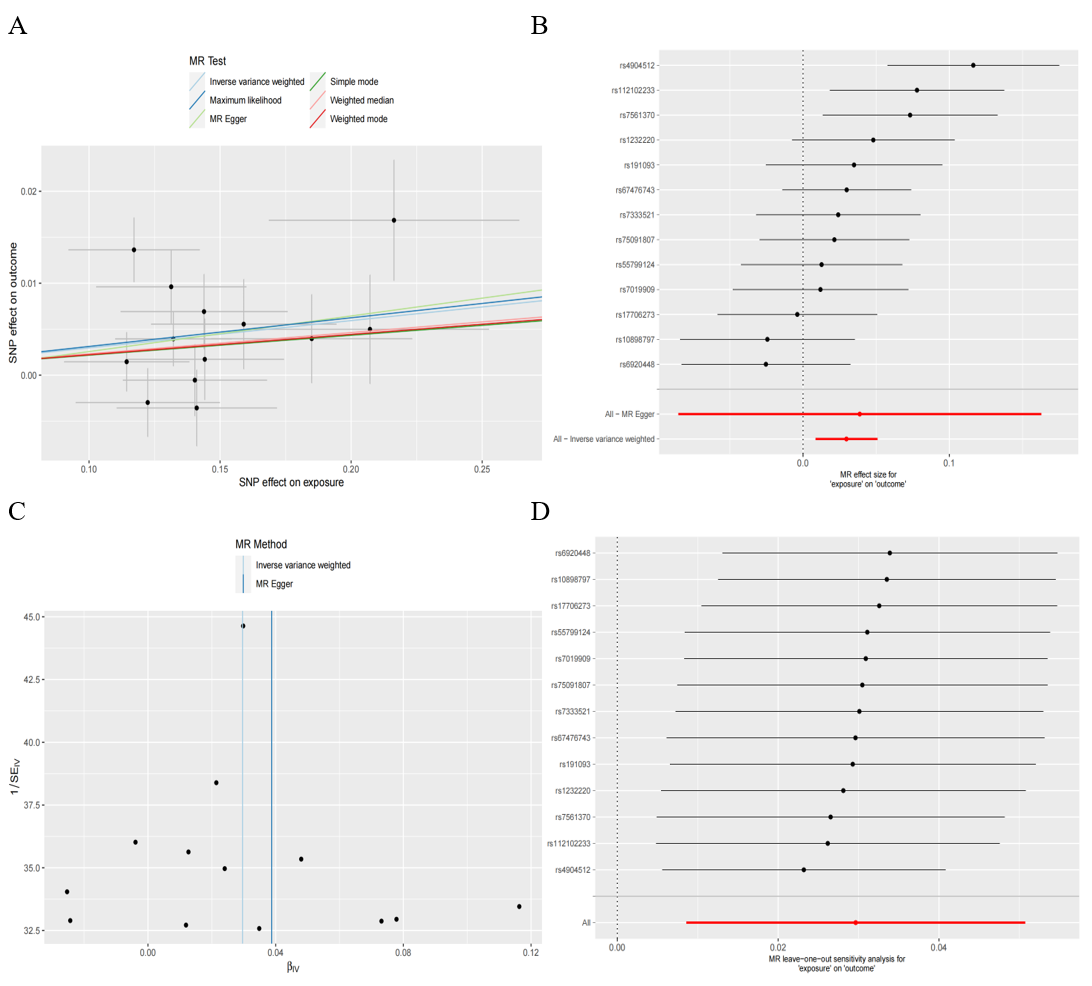
**

**Figure S73 Scatter plot (A), forest plot (B), funnel plot (C) and sensitivity analysis (D) of the causal effect of *Tyzzerella 3* on binary chronotype.**

**
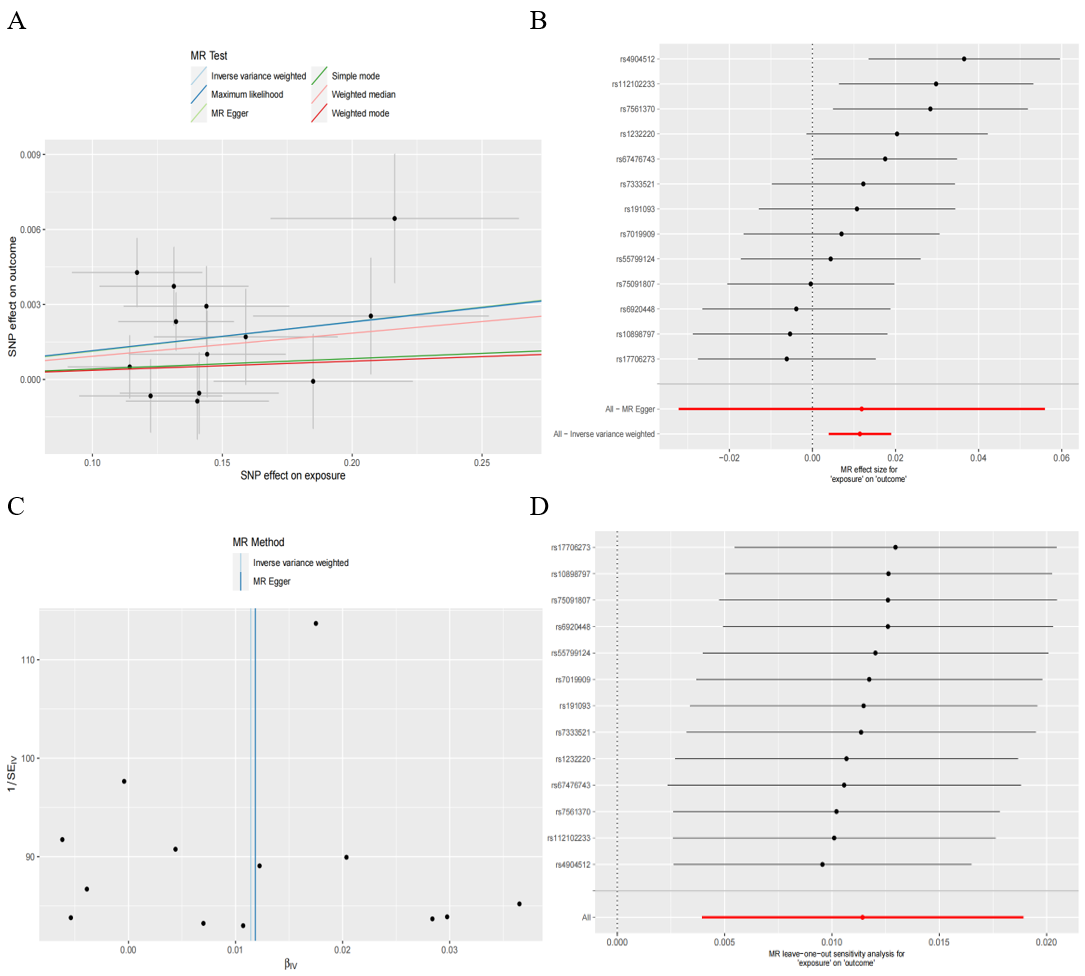
**

**Figure S74 Scatter plot (A), forest plot (B), funnel plot (C) and sensitivity analysis (D) of the causal effect of sleep duration on *Bacteroides*.**

**
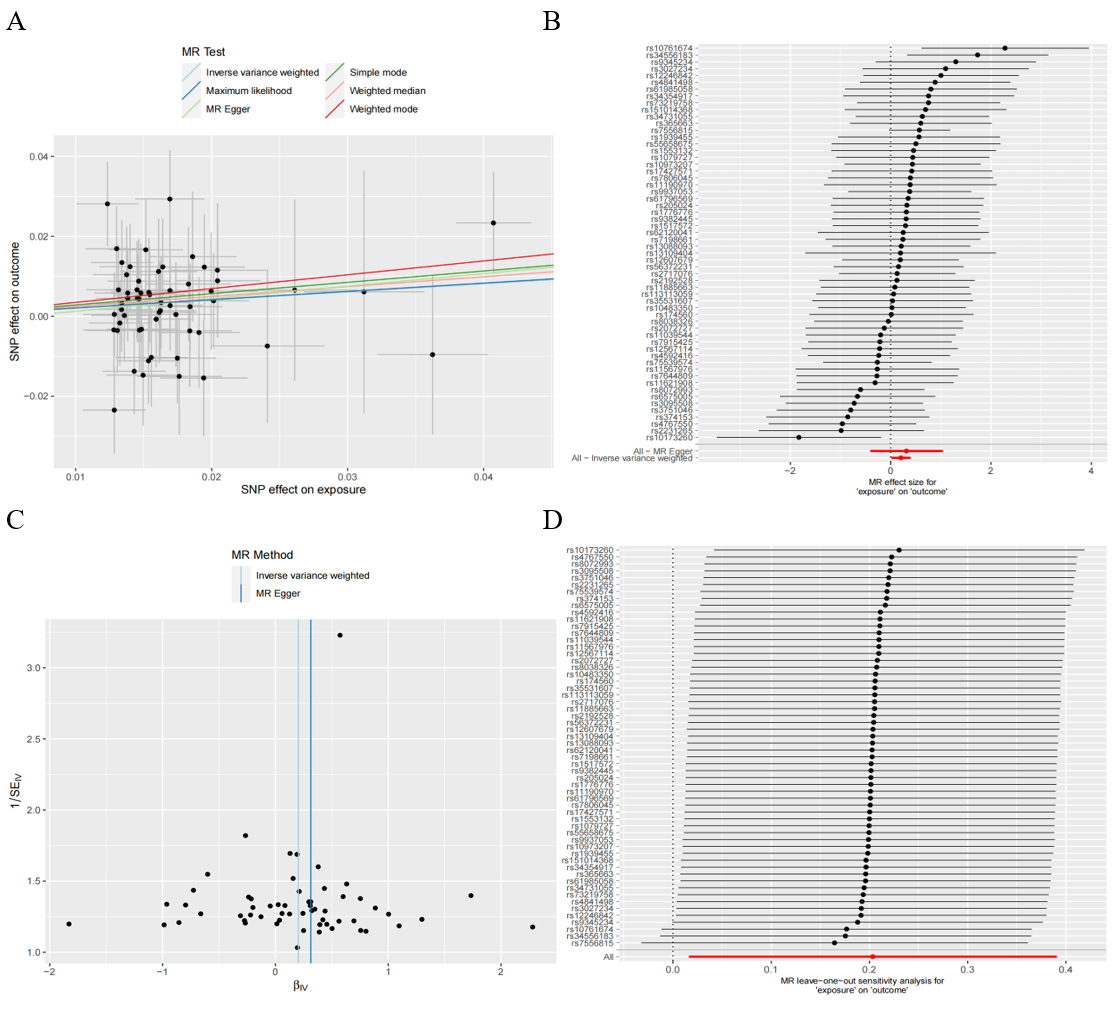
**

**Figure S75 Scatter plot (A), forest plot (B), funnel plot (C) and sensitivity analysis (D) of the causal effect of short sleep duration on *Bacteroides*.**

**
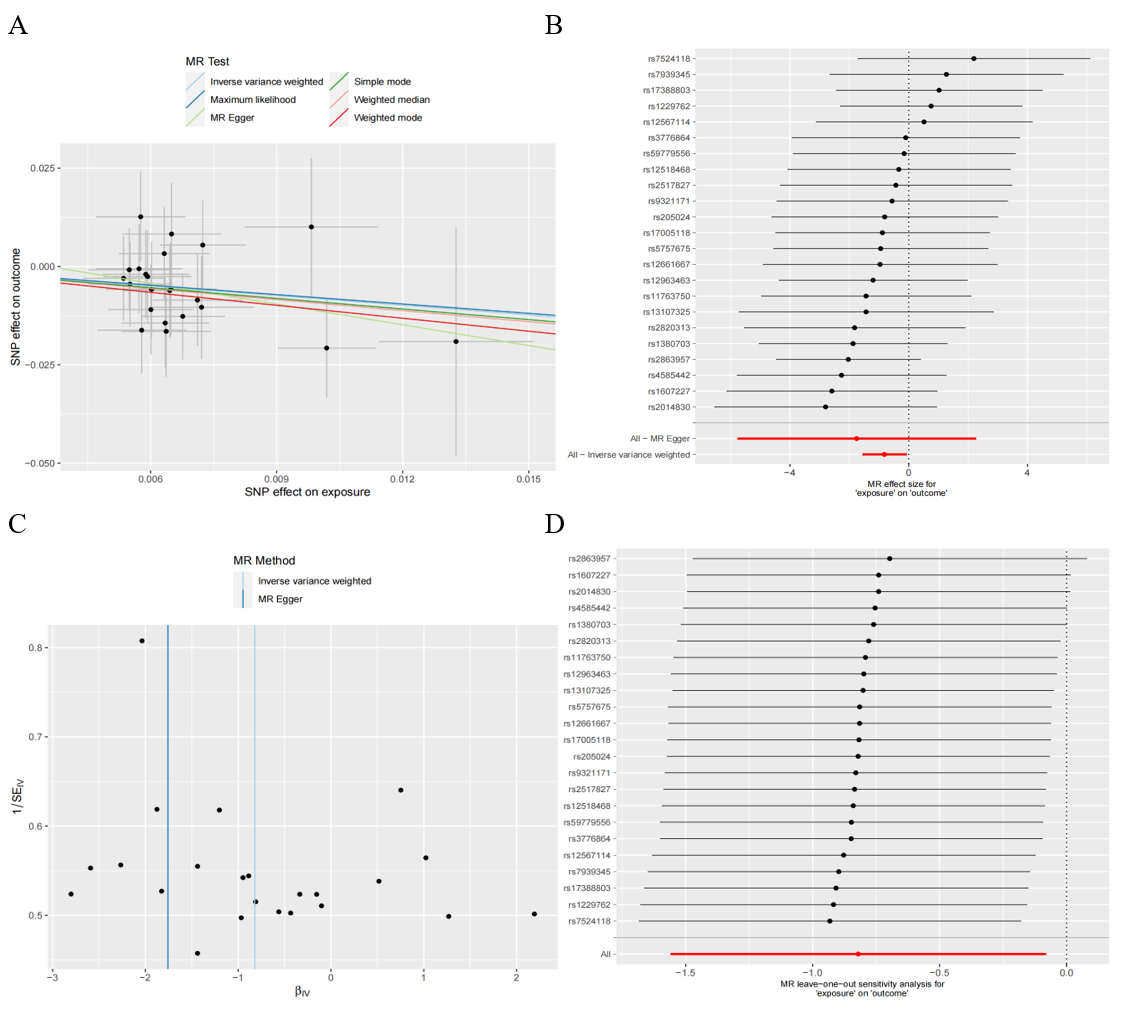
**

**Figure S76 Scatter plot (A), forest plot (B), funnel plot (C) and sensitivity analysis (D) of the causal effect of binary chronotype on *Bacteroides*.**

**
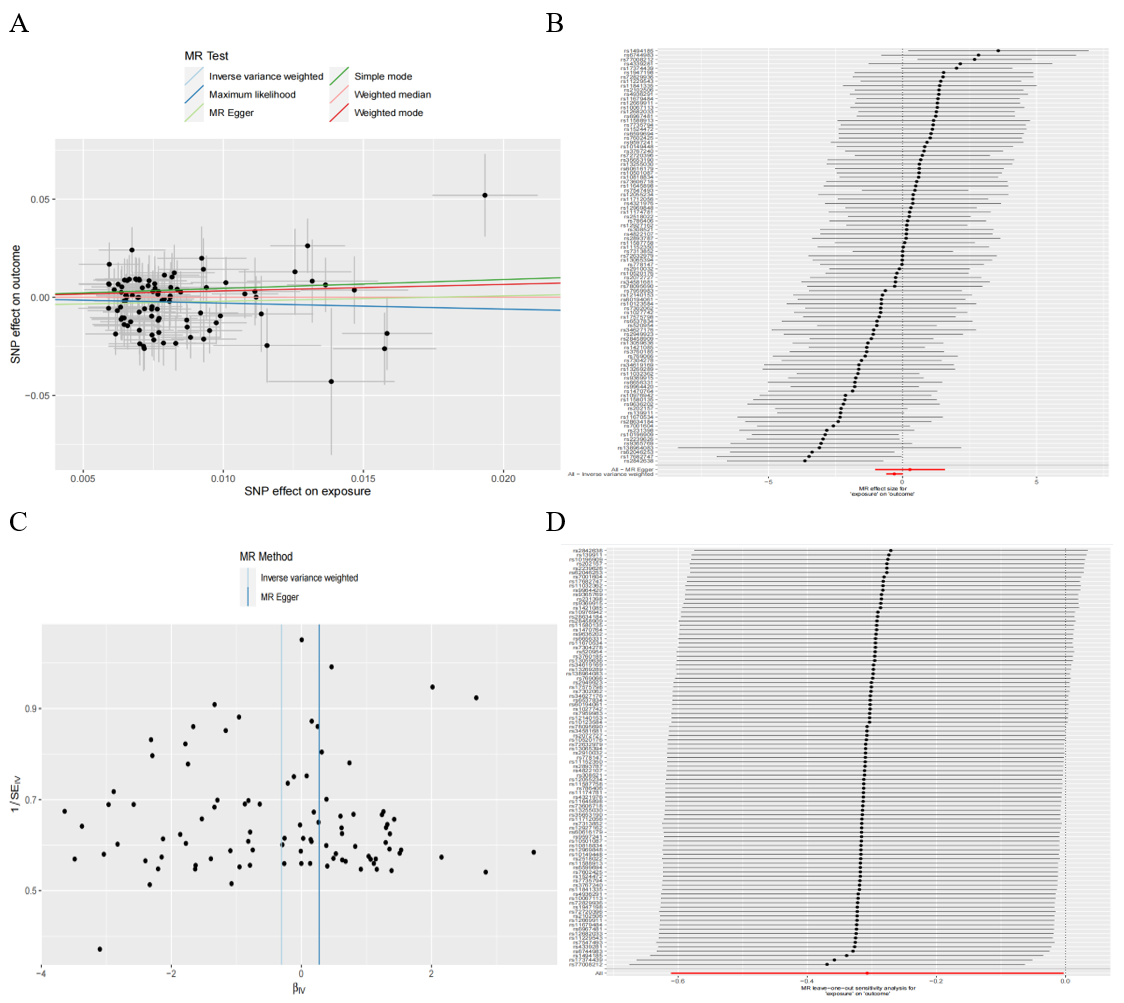
**

**Figure S77 Scatter plot (A), forest plot (B), funnel plot (C) and sensitivity analysis (D) of the causal effect of sleep duration on *Alloprevotella*.**

**
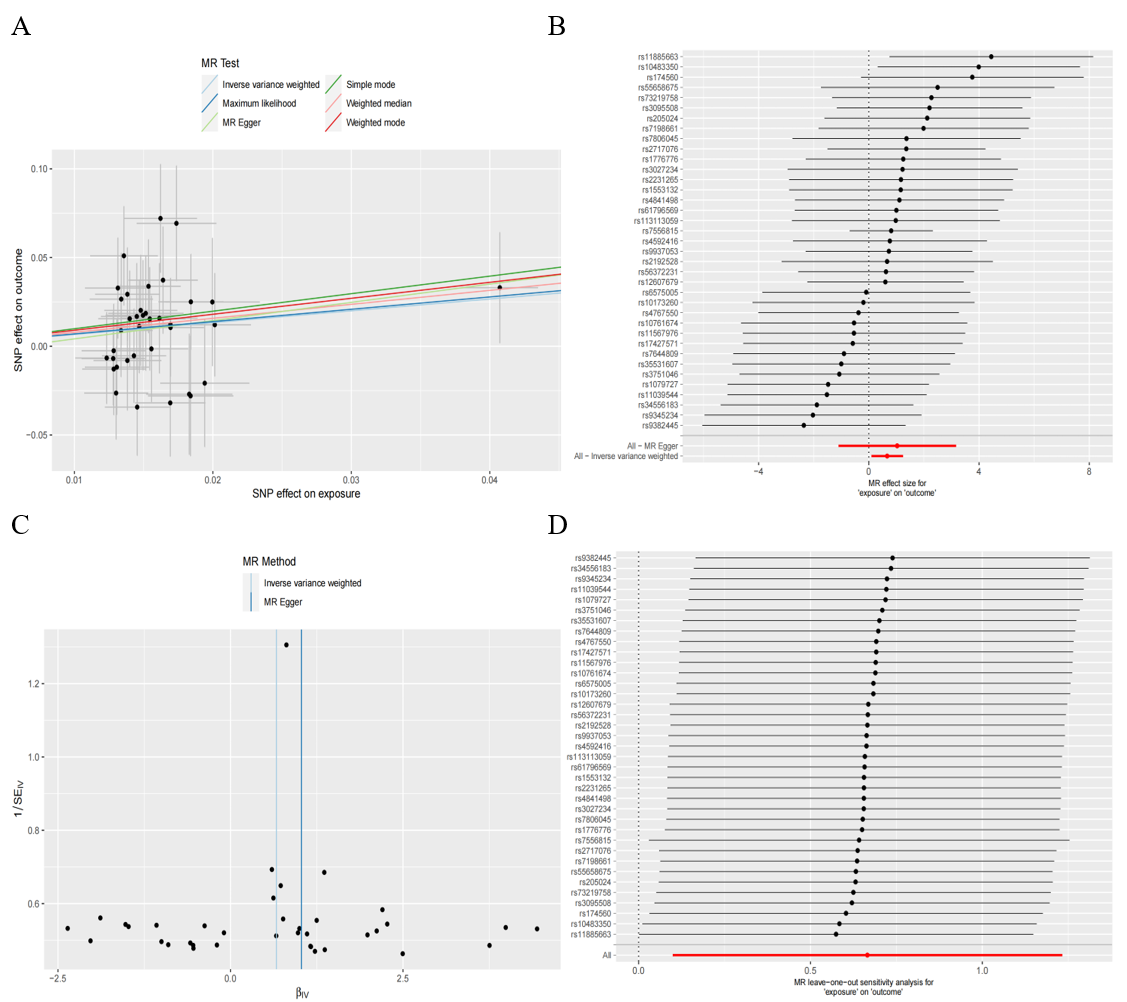
**

**Figure S78 Scatter plot (A), forest plot (B), funnel plot (C) and sensitivity analysis (D) of the causal effect of sleep duration on *Prevotella 9*.**

**
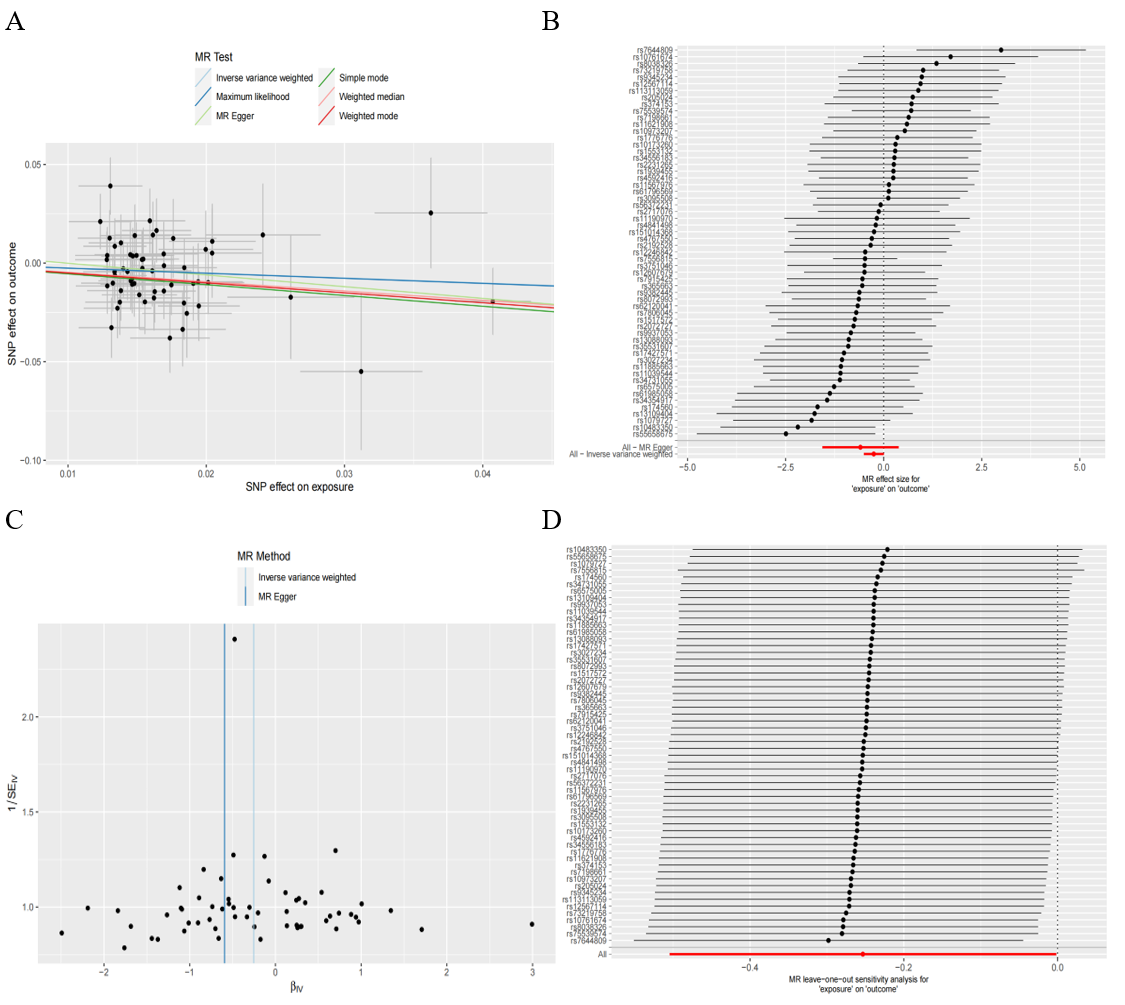
**

**Figure S79 Scatter plot (A), forest plot (B), funnel plot (C) and sensitivity analysis (D) of the causal effect of binary chronotype on *Alistipes*.**

**
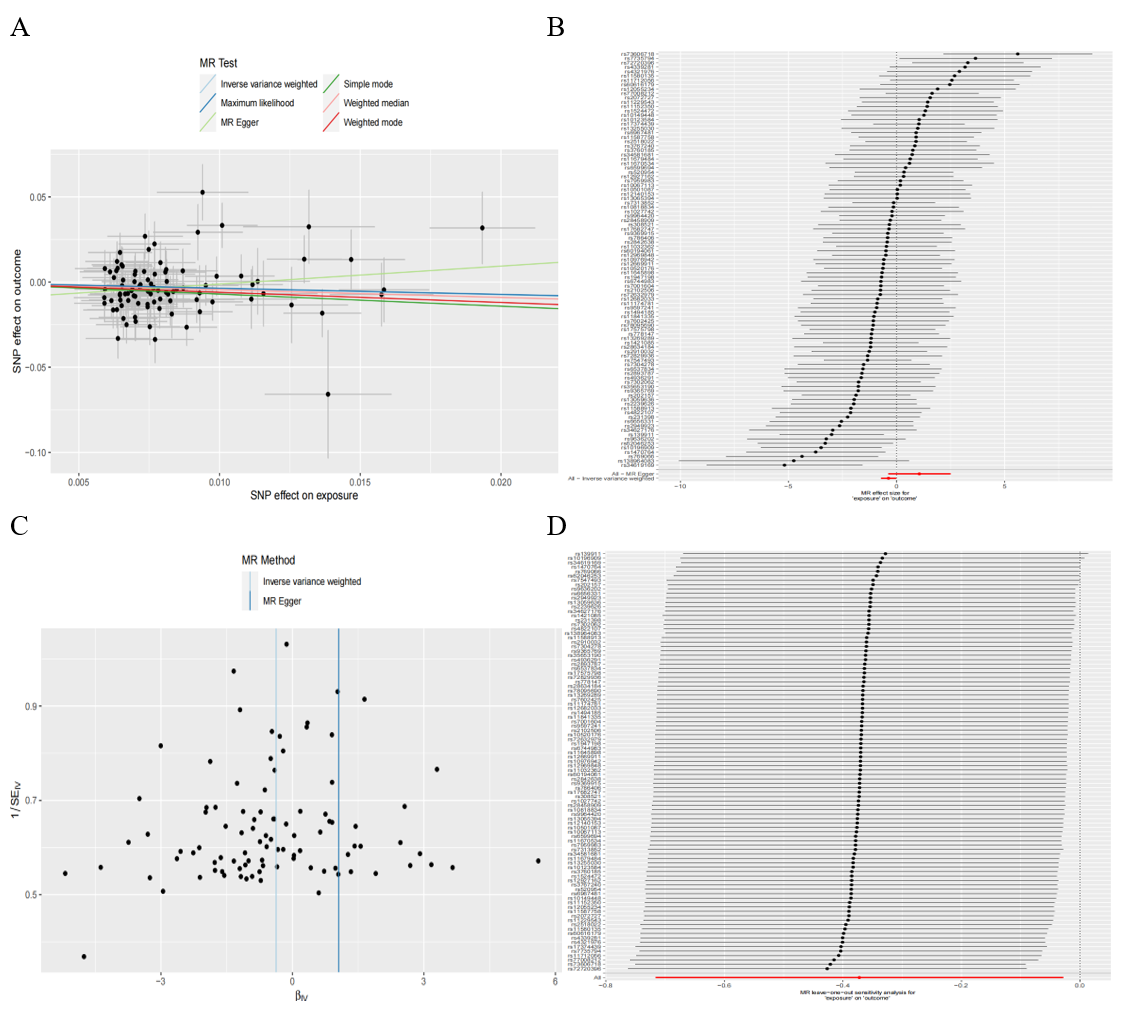
**

**Figure S80 Scatter plot (A), forest plot (B), funnel plot (C) and sensitivity analysis (D) of the causal effect of short sleep duration on *Parabacteroides*.**

**
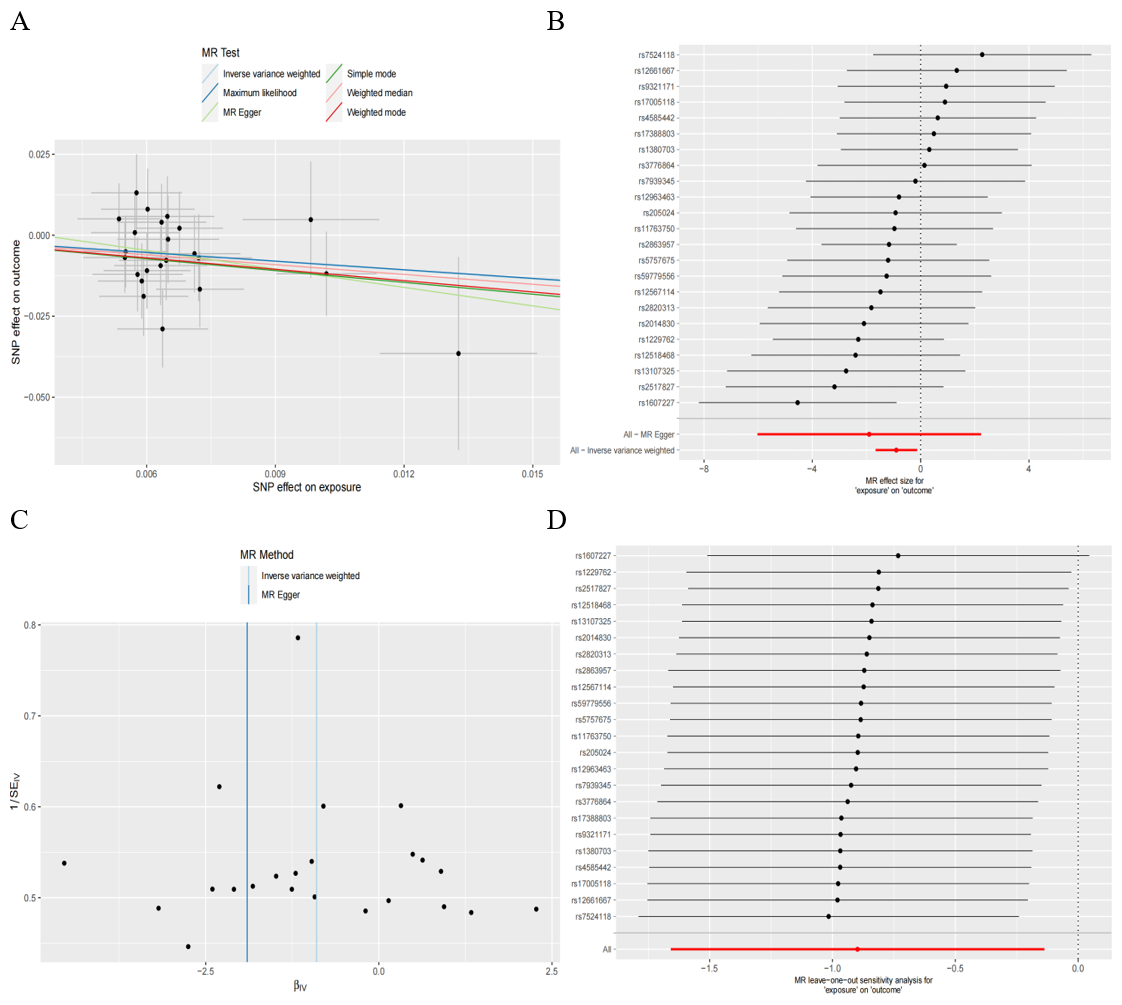
**

**Figure S81 Scatter plot (A), forest plot (B), funnel plot (C) and sensitivity analysis (D) of the causal effect of chronotype on *Parabacteroides*.**

**
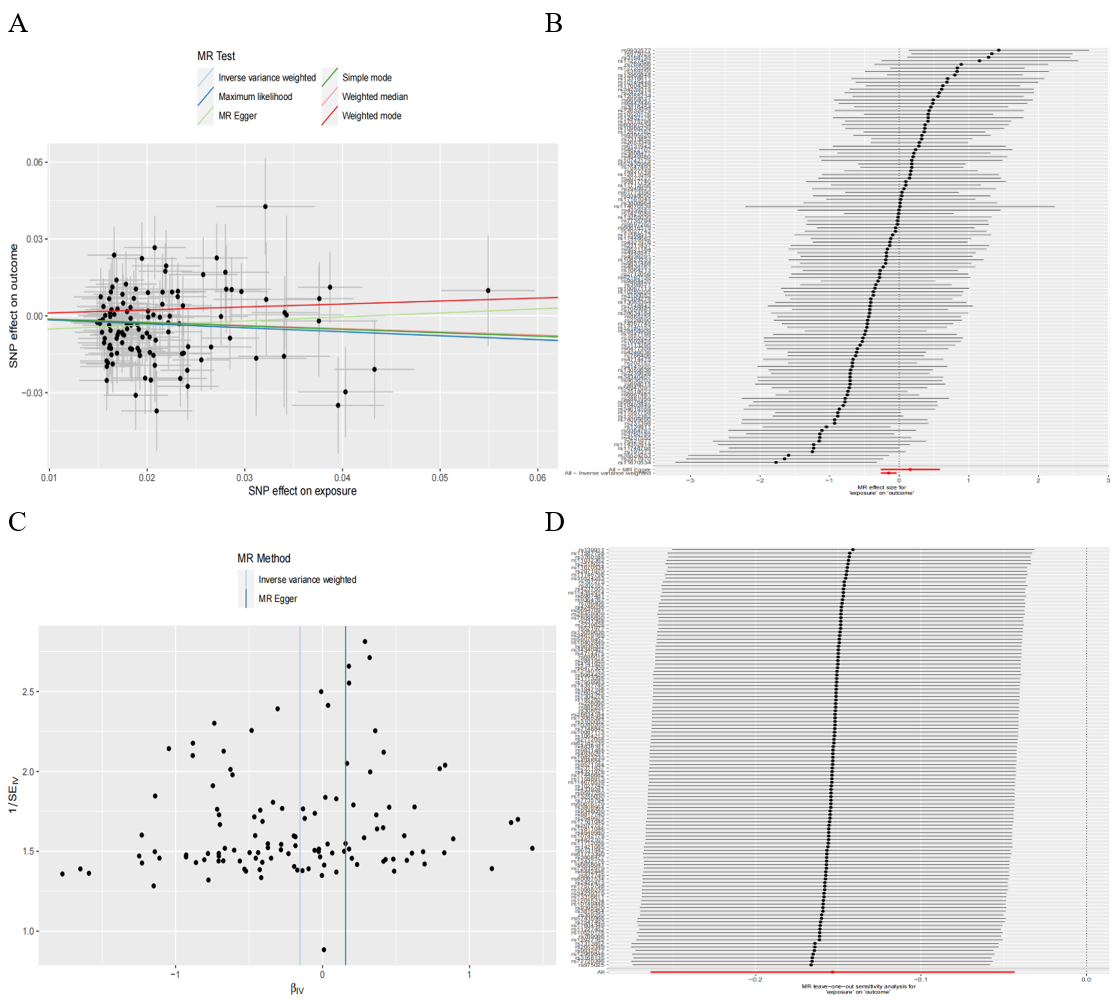
**

**Figure S82 Scatter plot (A), forest plot (B), funnel plot (C) and sensitivity analysis (D) of the causal effect of binary chronotype on *Parabacteroides*.**

**
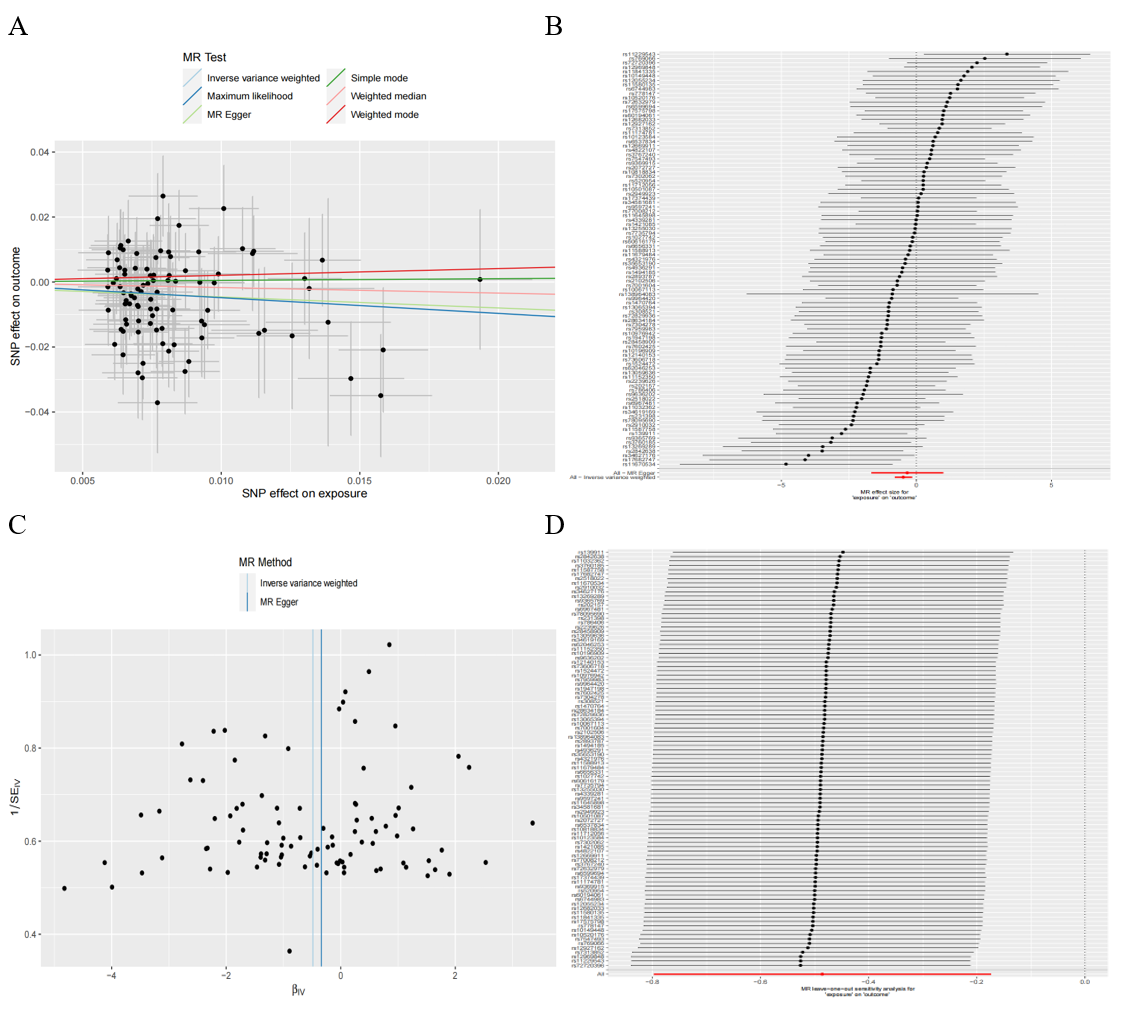
**

**Figure S83 Scatter plot (A), forest plot (B), funnel plot (C) and sensitivity analysis (D) of the causal effect of daytime sleepiness on *Oxalobacter*.**

**
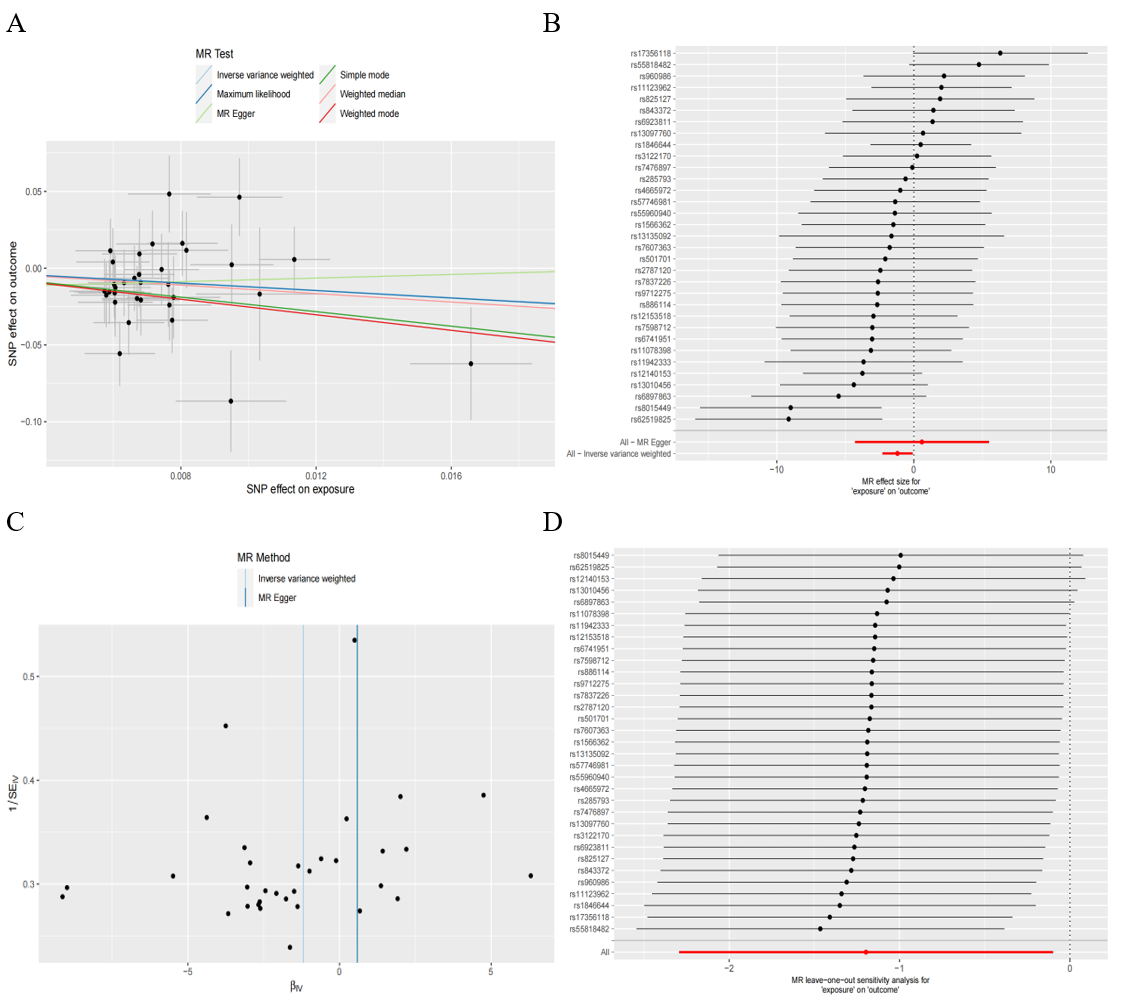
**

**Figure S84 Scatter plot (A), forest plot (B), funnel plot (C) and sensitivity analysis (D) of the causal effect of daytime sleepiness adjusted for BMI on *Oxalobacter*.**

**
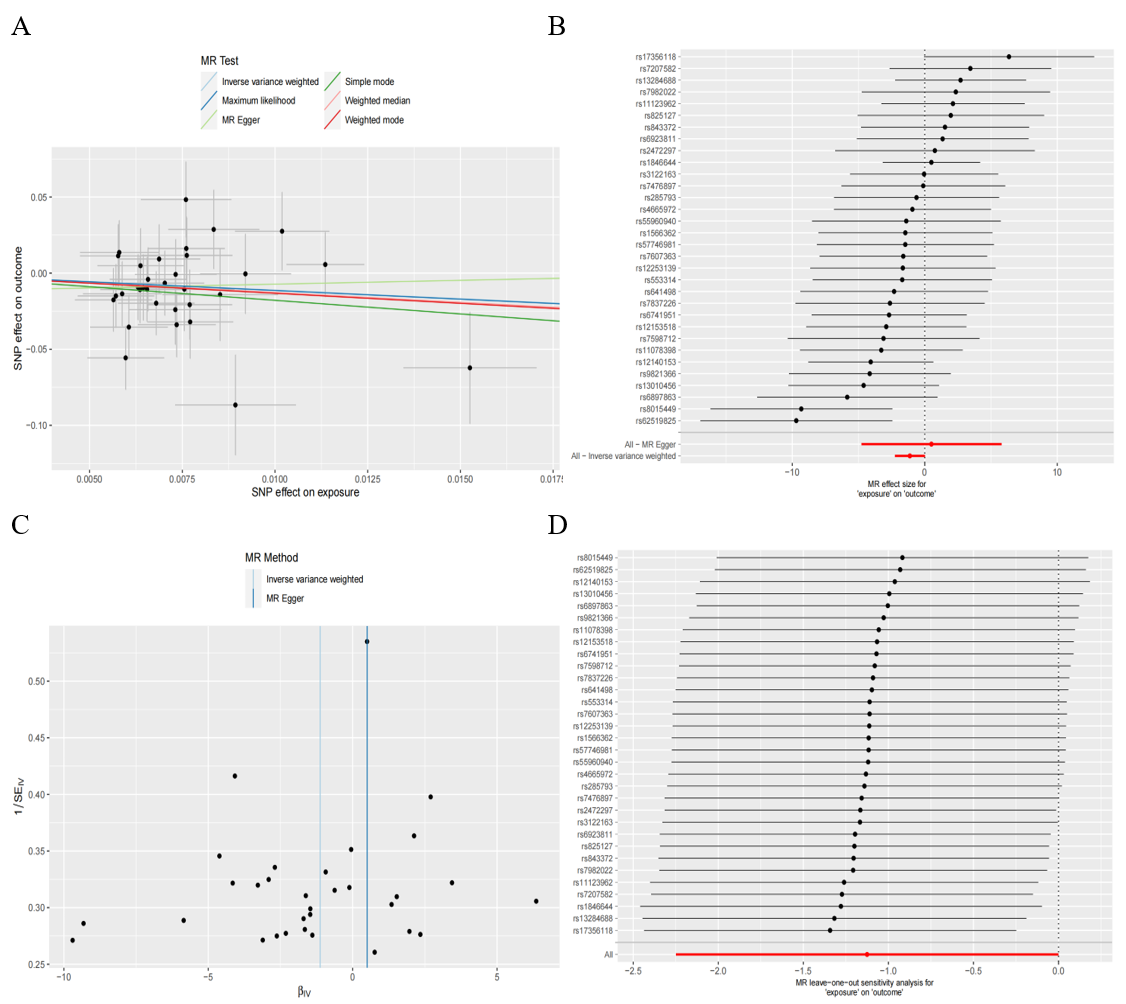
**

**Figure S85 Scatter plot (A), forest plot (B), funnel plot (C) and sensitivity analysis (D) of the causal effect of insomnia on *Oxalobacter*.**

**
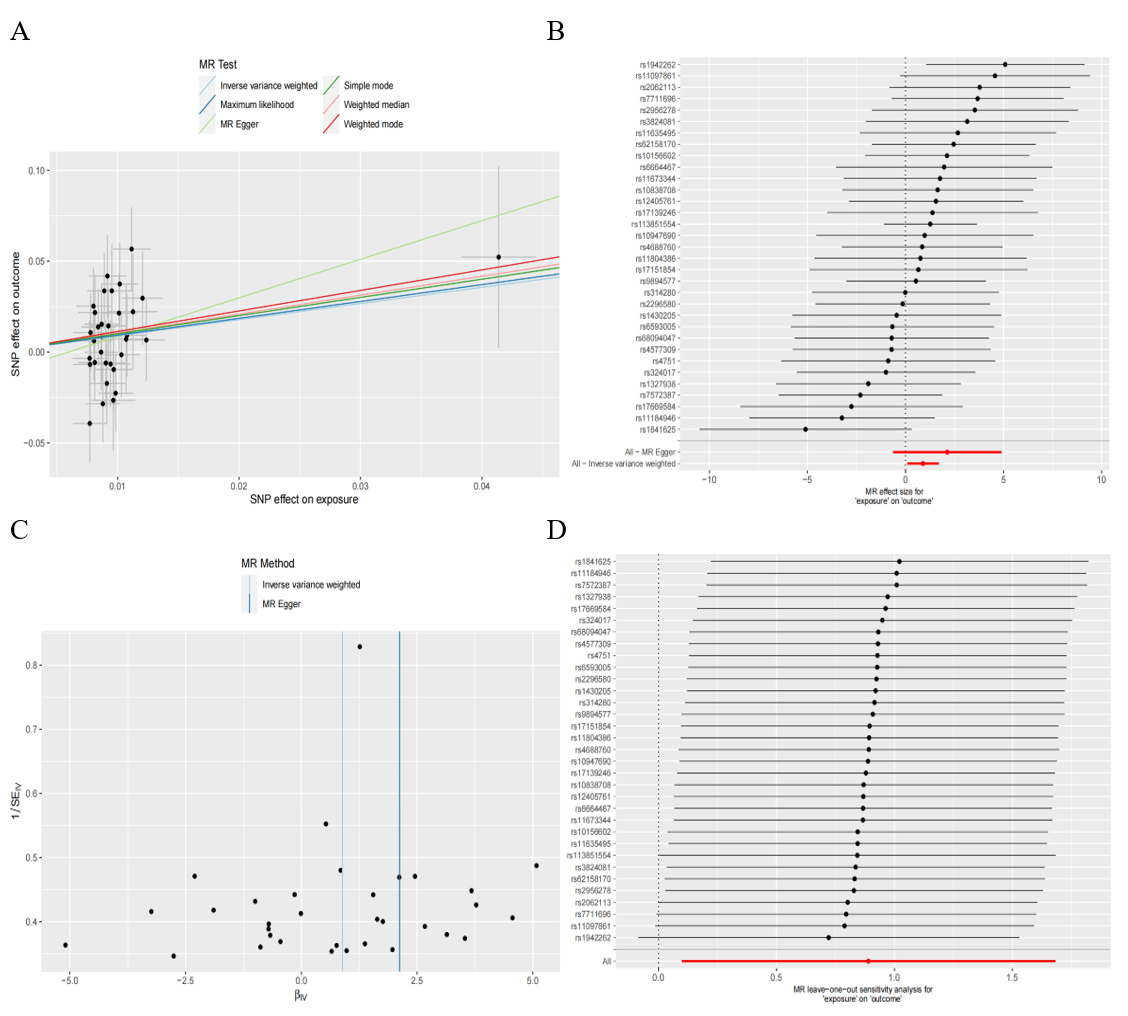
**

**Figure S86 Scatter plot (A), forest plot (B), funnel plot (C) and sensitivity analysis (D) of the causal effect of short sleep duration on *Haemophilus*.**

**
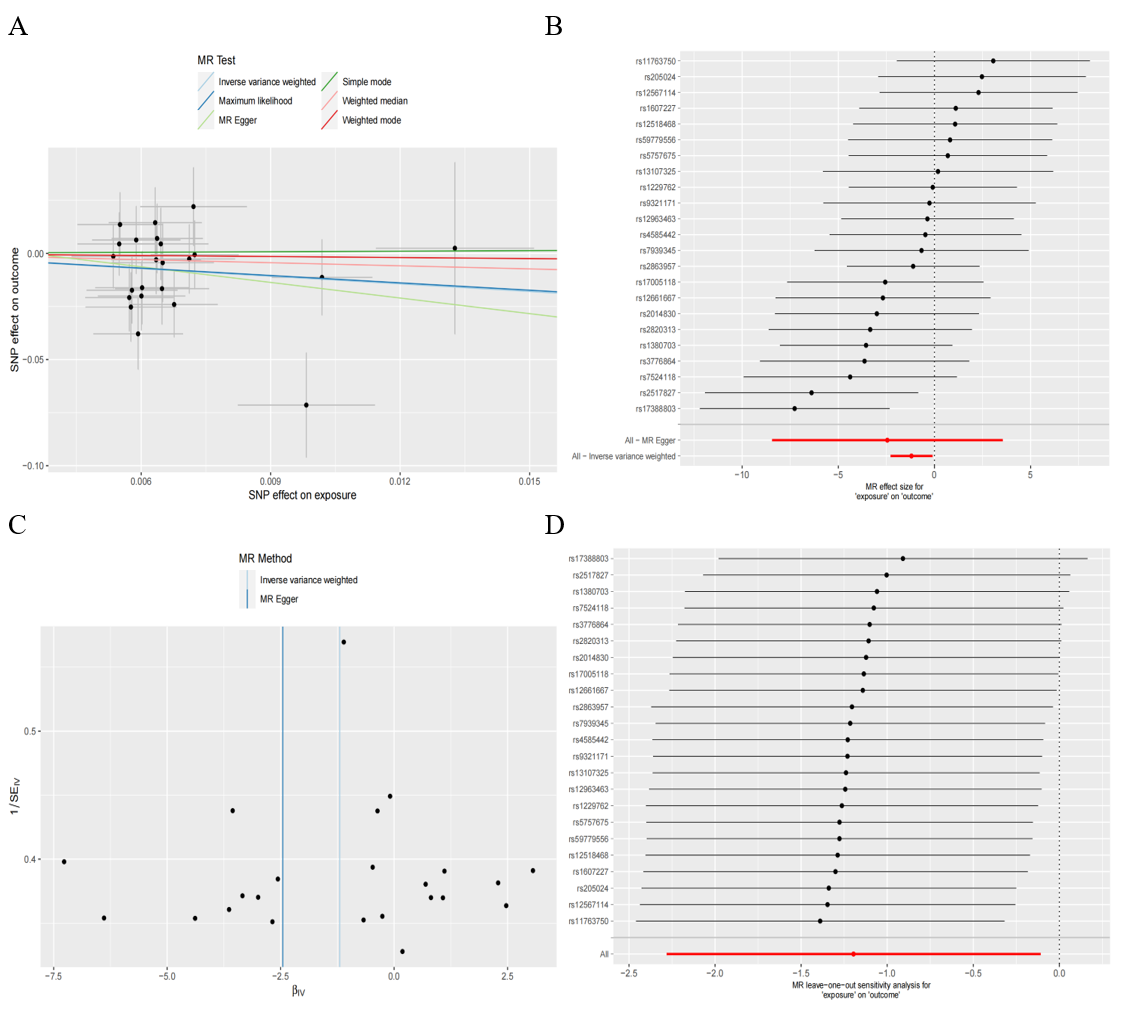
**

**Figure S87 Scatter plot (A), forest plot (B), funnel plot (C) and sensitivity analysis (D) of the causal effect of daytime napping on *Akkermansia*.**

**
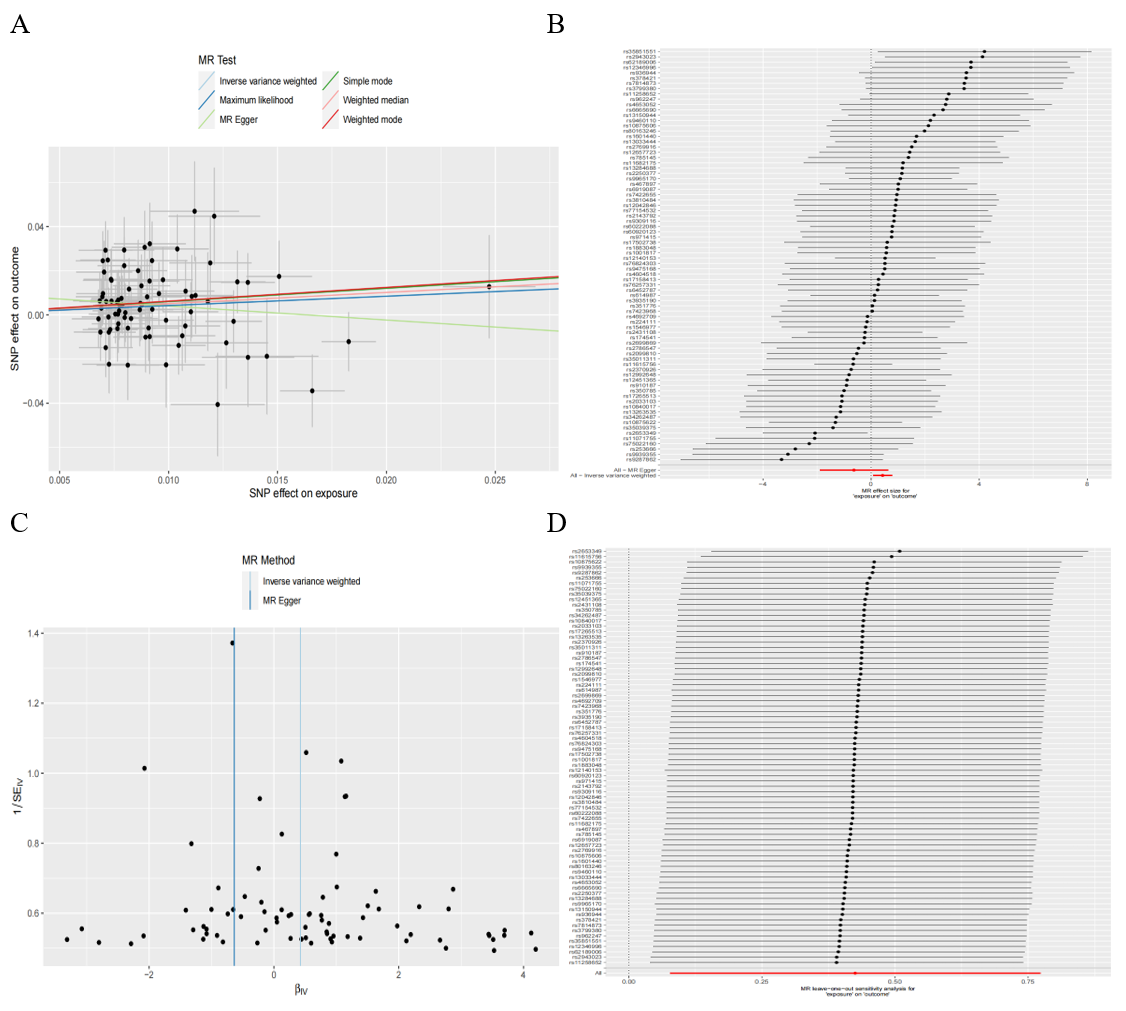
**

**Figure S88 Scatter plot (A), forest plot (B), funnel plot (C) and sensitivity analysis (D) of the causal effect of sleep duration on *Senegalimassilia*.**

**
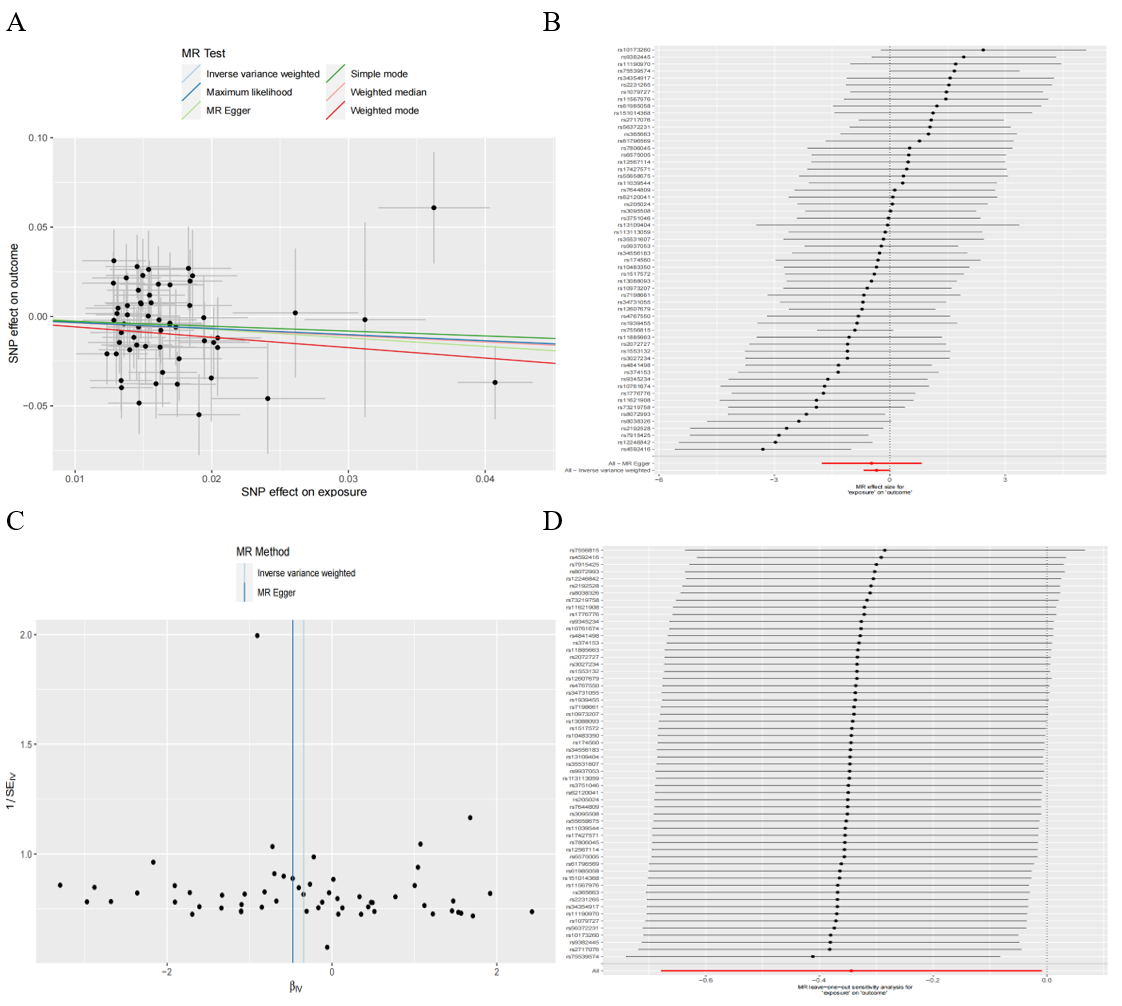
**

**Figure S89 Scatter plot (A), forest plot (B), funnel plot (C) and sensitivity analysis (D) of the causal effect of long sleep duration on *Adlercreutzia*.**

**
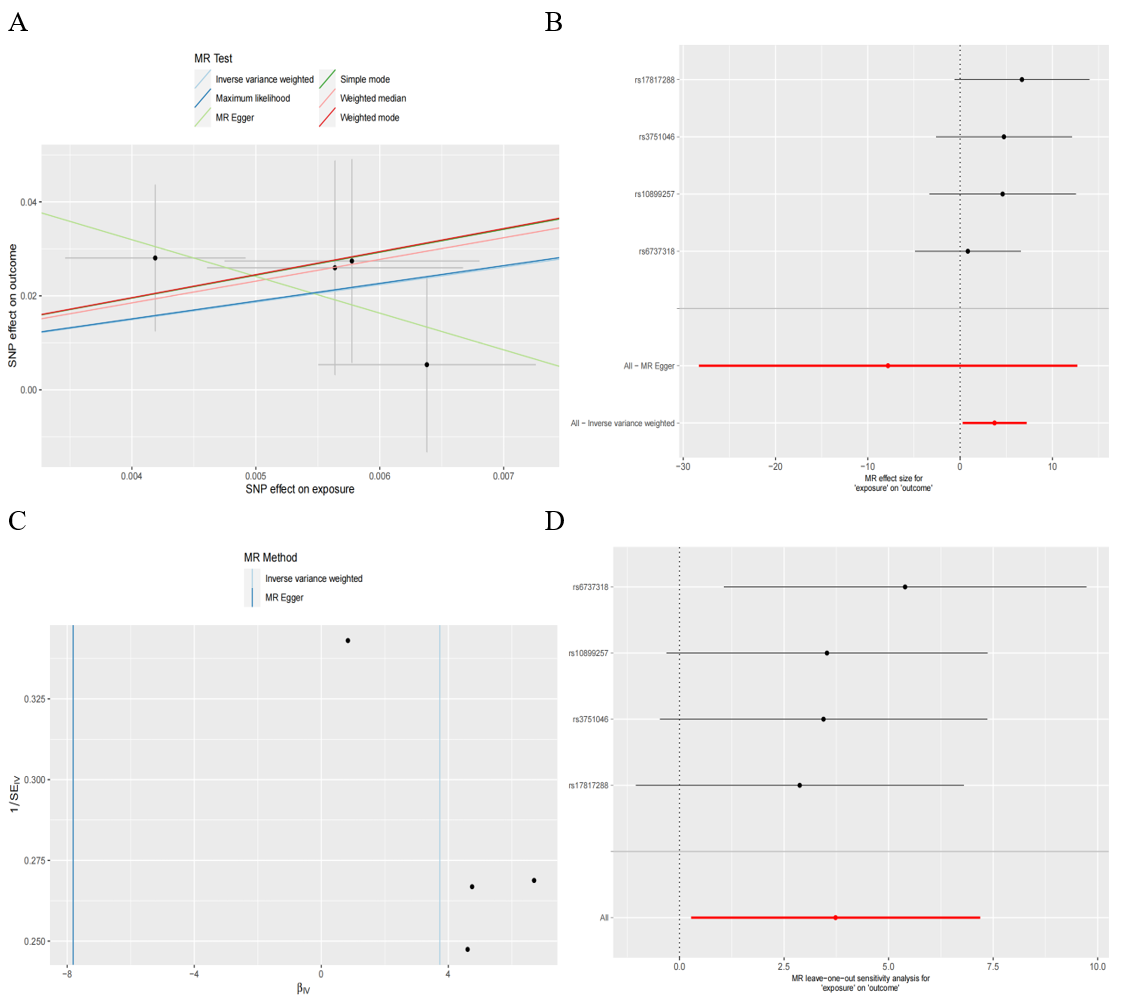
**

**Figure S90 Scatter plot (A), forest plot (B), funnel plot (C) and sensitivity analysis (D) of the causal effect of short sleep duration on *Eggerthella*.**

**
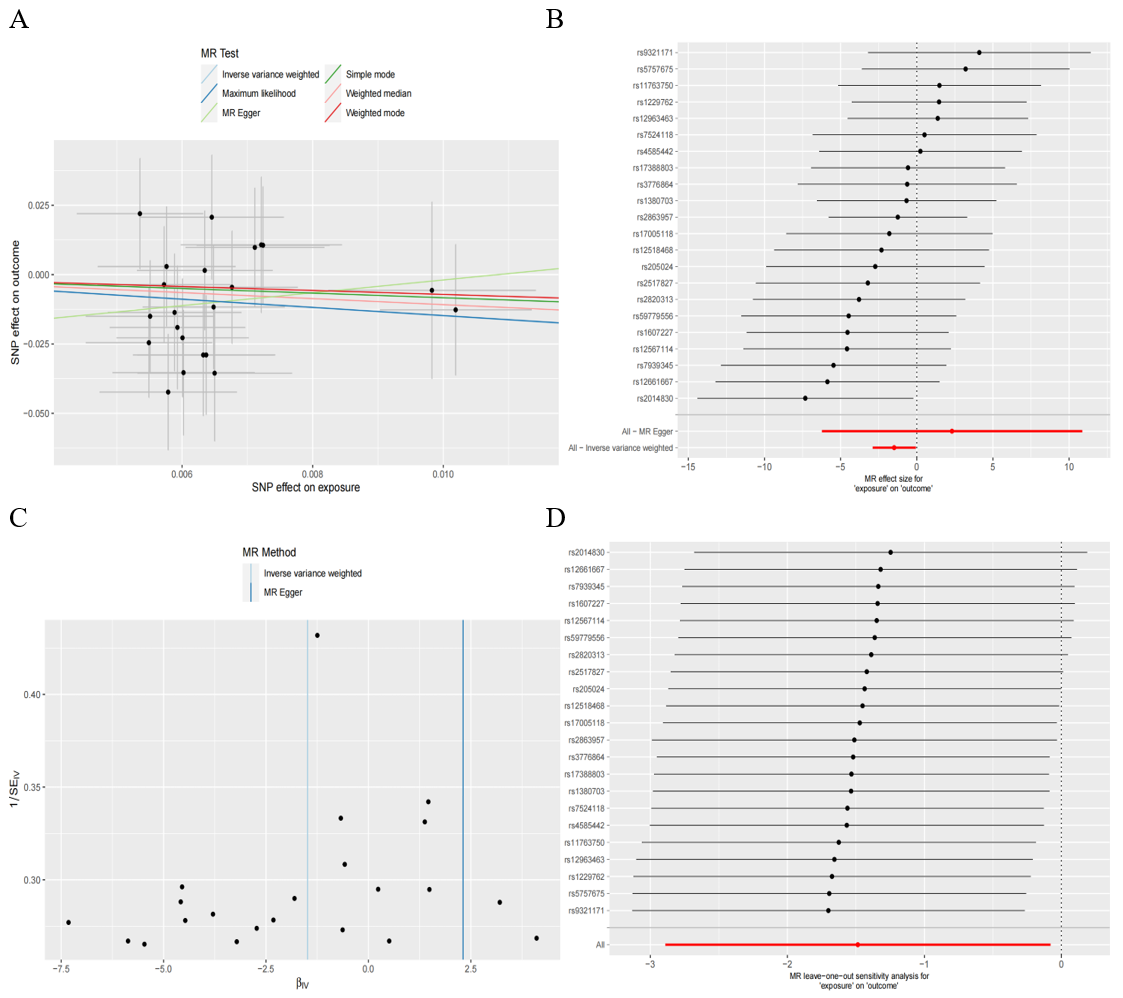
**

**Figure S91 Scatter plot (A), forest plot (B), funnel plot (C) and sensitivity analysis (D) of the causal effect of binary chronotype on *Eggerthella*.**

**
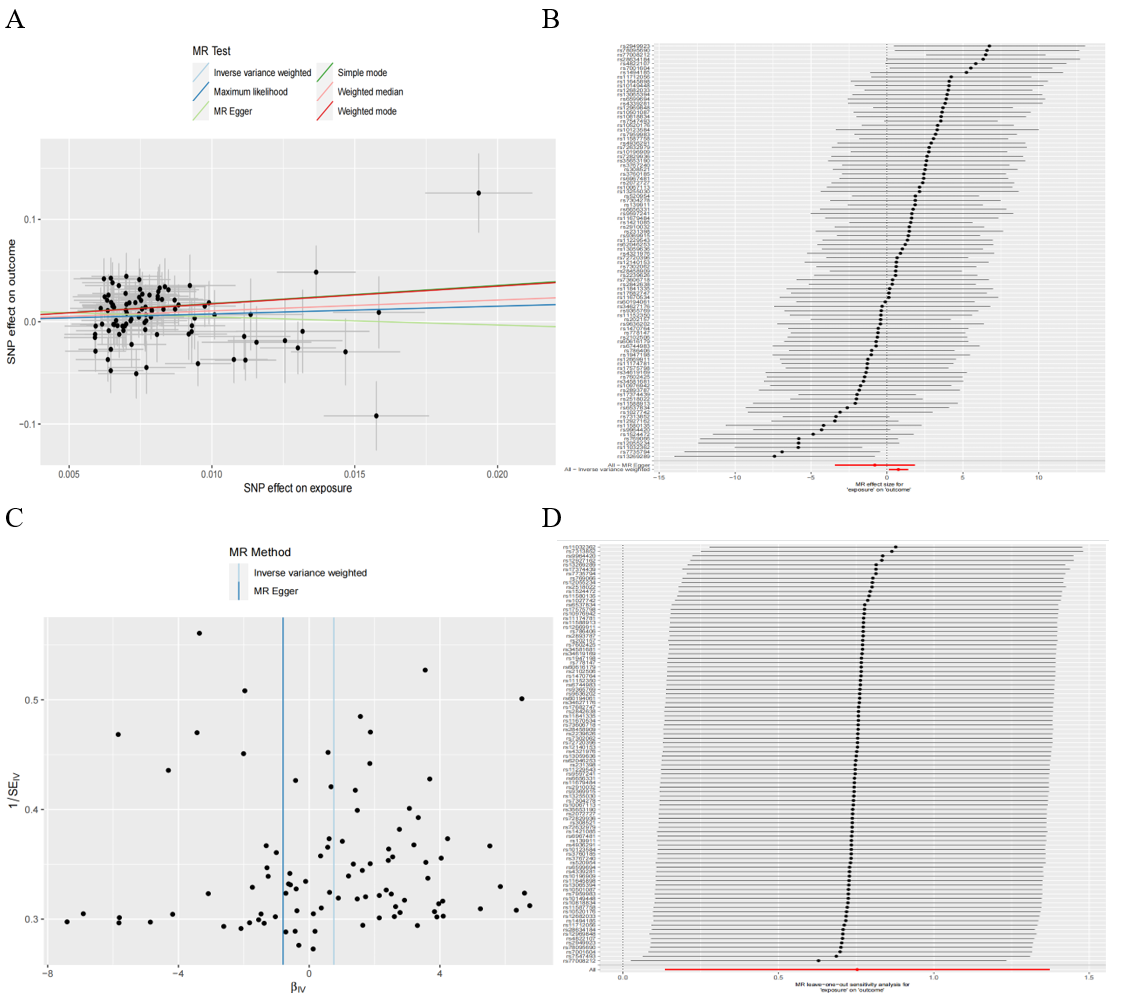
**

**Figure S92 Scatter plot (A), forest plot (B), funnel plot (C) and sensitivity analysis (D) of the causal effect of daytime sleepiness on *Slackia*.**

**
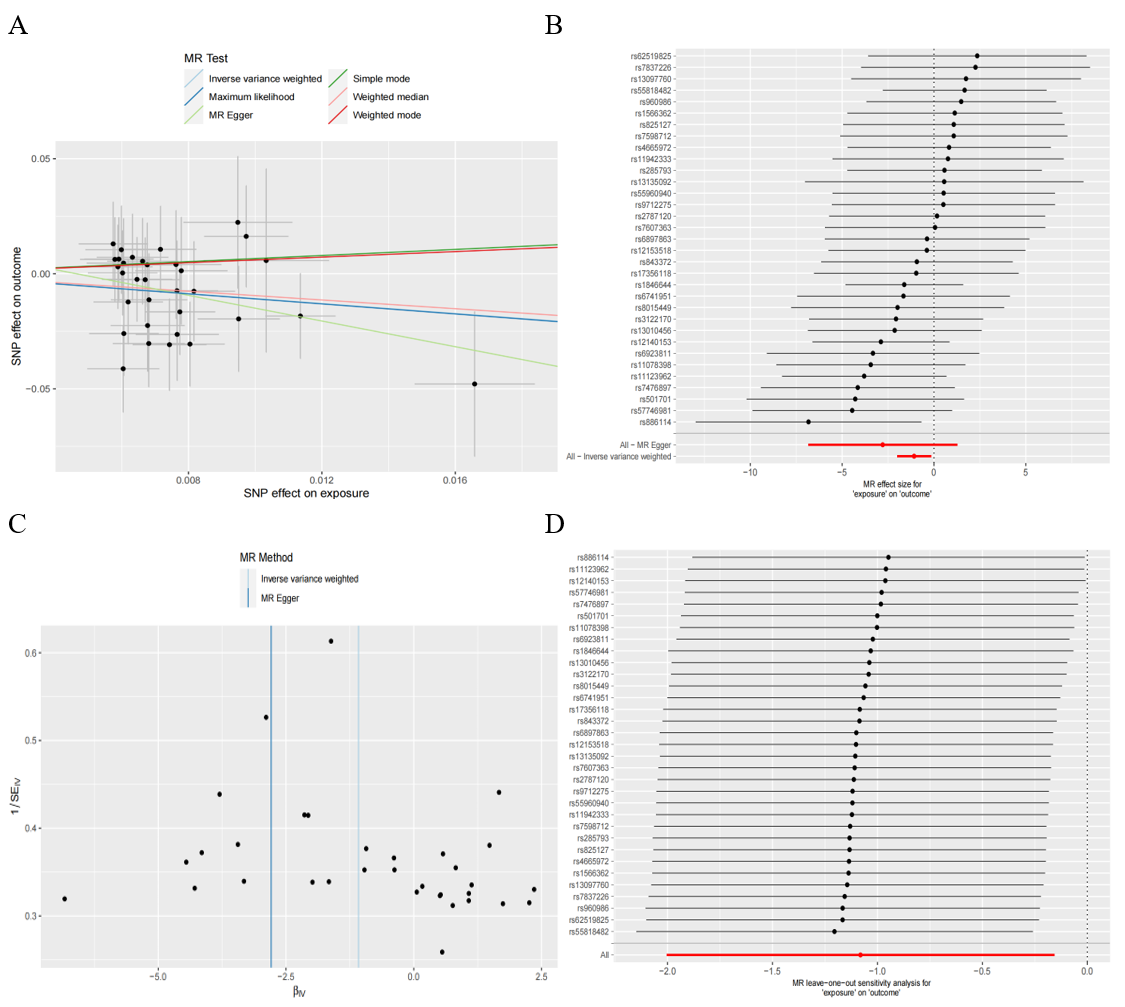
**

**Figure S93 Scatter plot (A), forest plot (B), funnel plot (C) and sensitivity analysis (D) of the causal effect of sleep duration on *Lactobacillus*.**

**
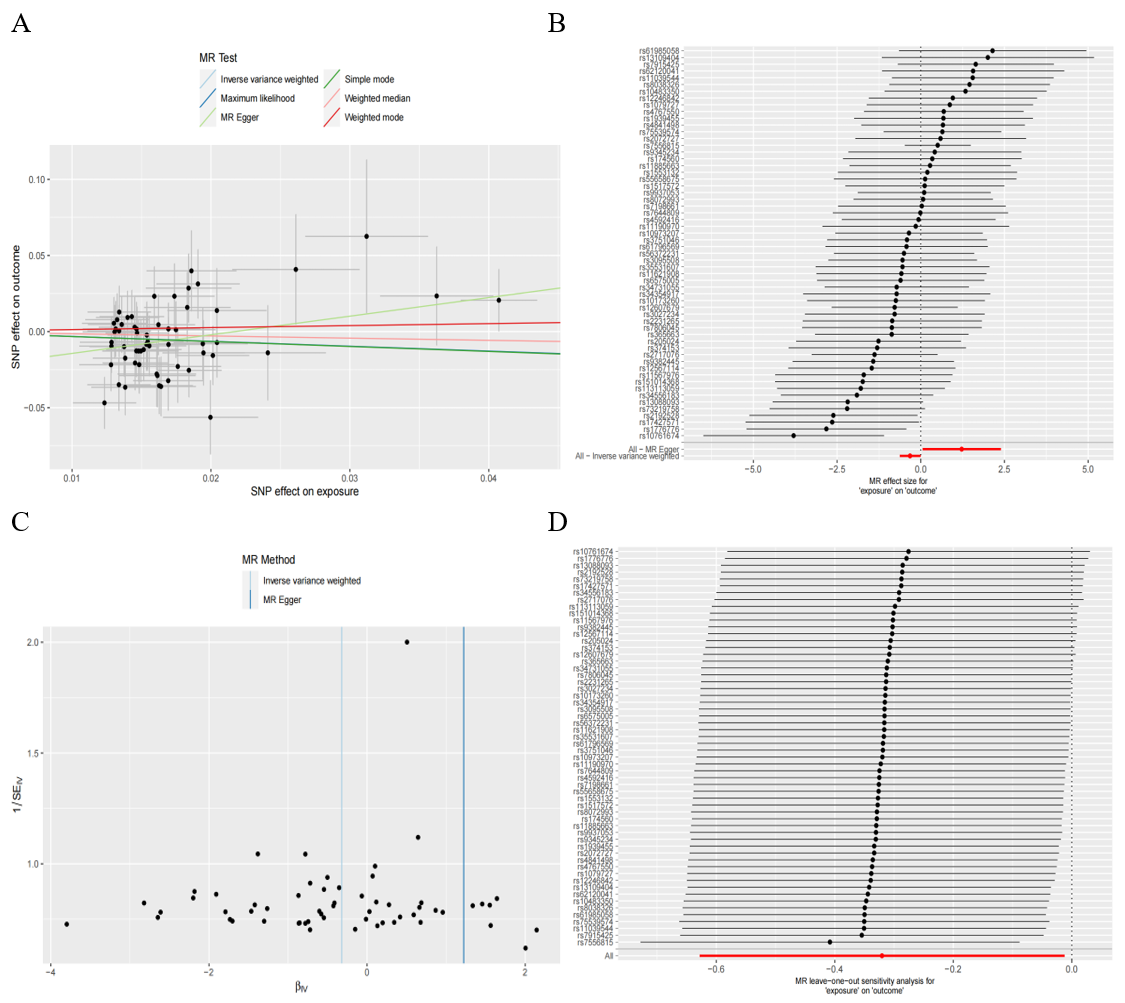
**

**Figure S94 Scatter plot (A), forest plot (B), funnel plot (C) and sensitivity analysis (D) of the causal effect of chronotype on *Streptococcus*.**

**
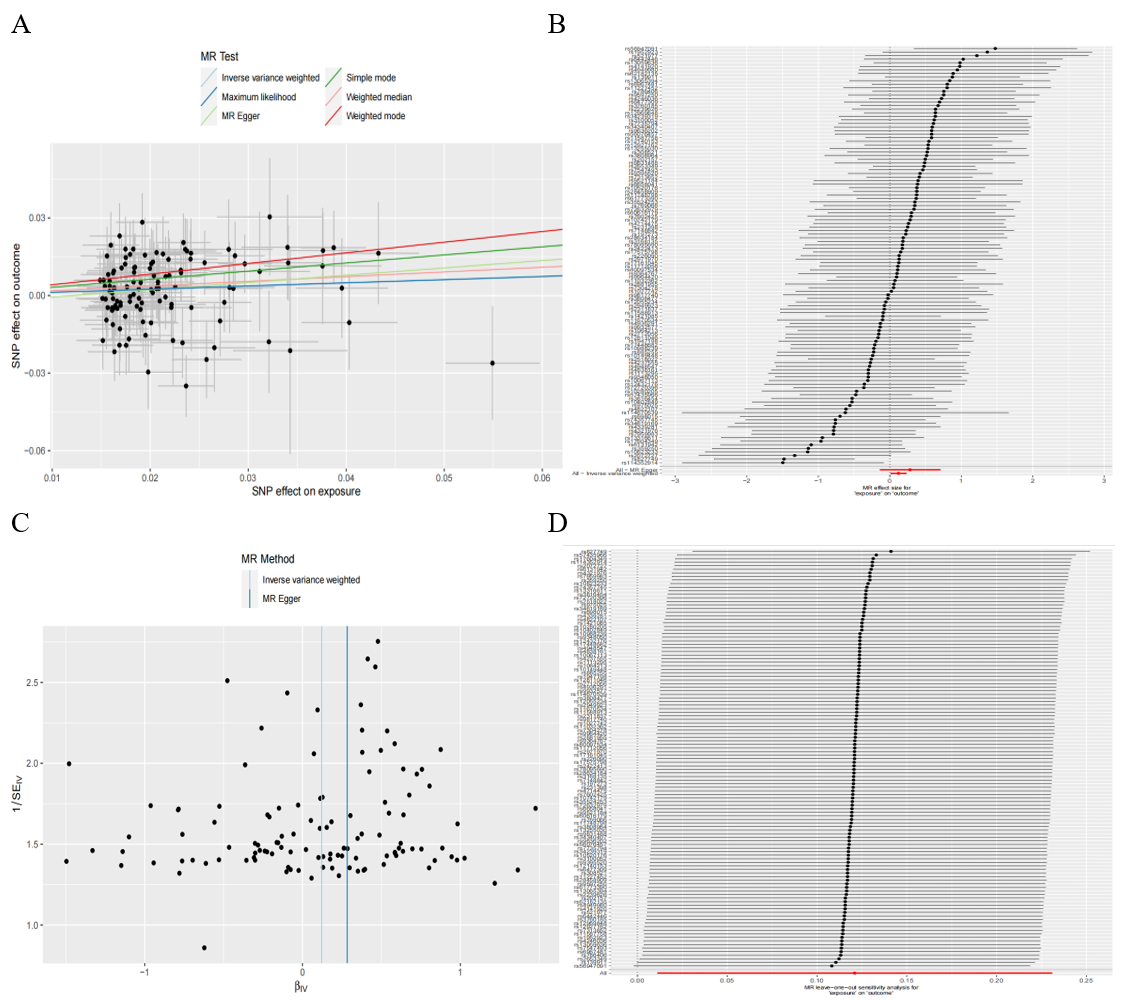
**

**Figure S95 Scatter plot (A), forest plot (B), funnel plot (C) and sensitivity analysis (D) of the causal effect of binary chronotype on *Streptococcus*.**

**
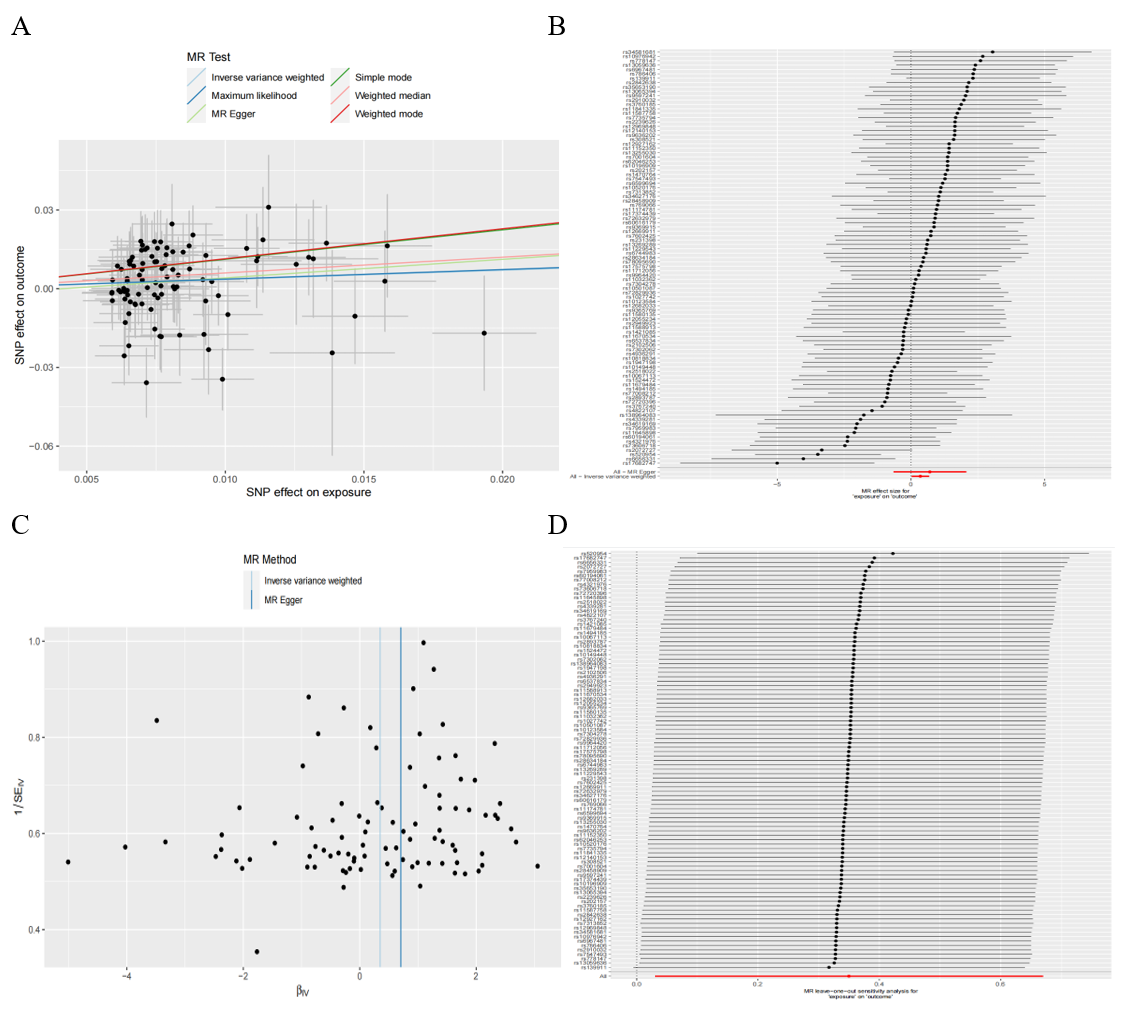
**

**Figure S96 Scatter plot (A), forest plot (B), funnel plot (C) and sensitivity analysis (D) of the causal effect of long sleep duration on *Lachnospiraceae ND3007 group*.**

**
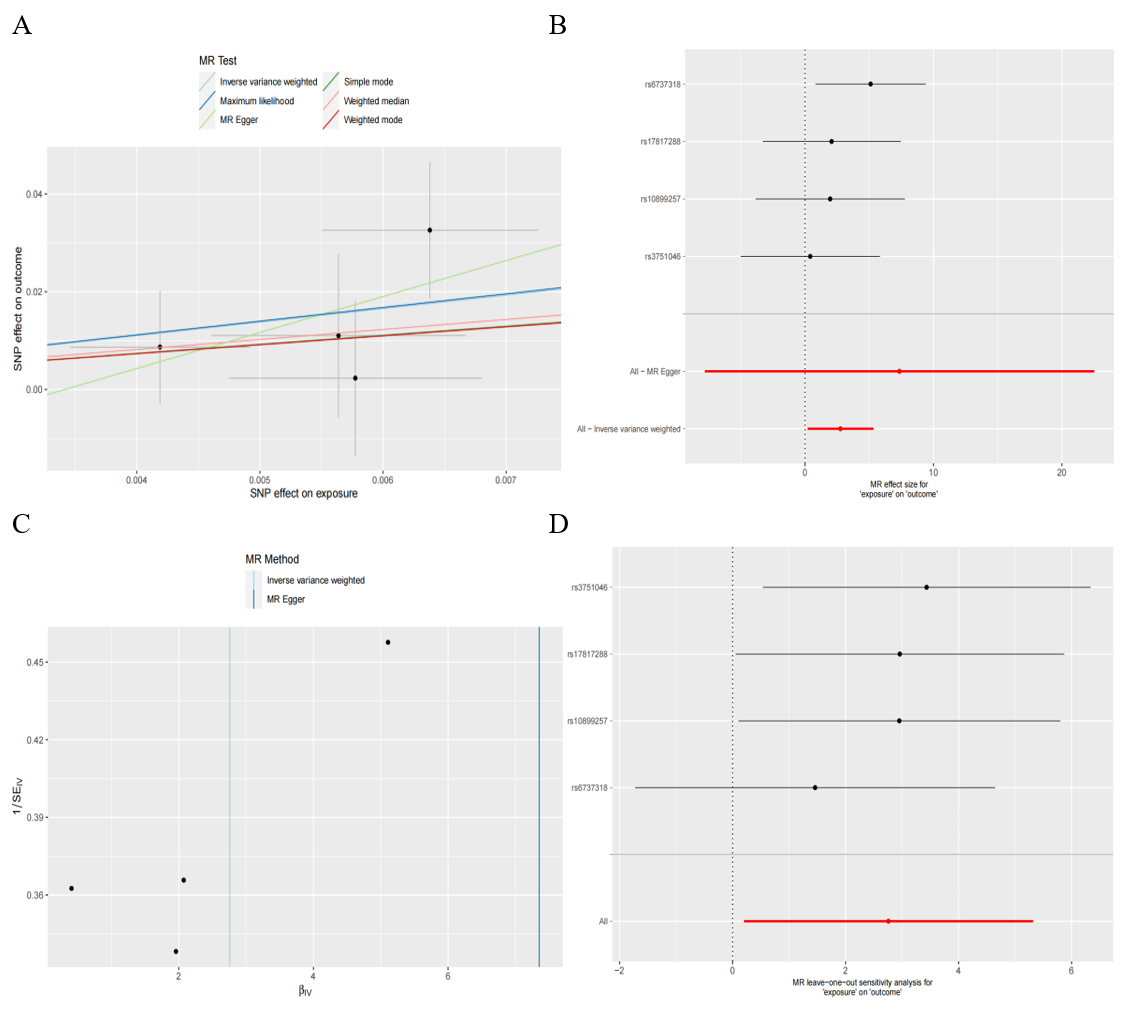
**

**Figure S97 Scatter plot (A), forest plot (B), funnel plot (C) and sensitivity analysis (D) of the causal effect of daytime napping on *Ruminococcaceae NK4A214 group*.**

**
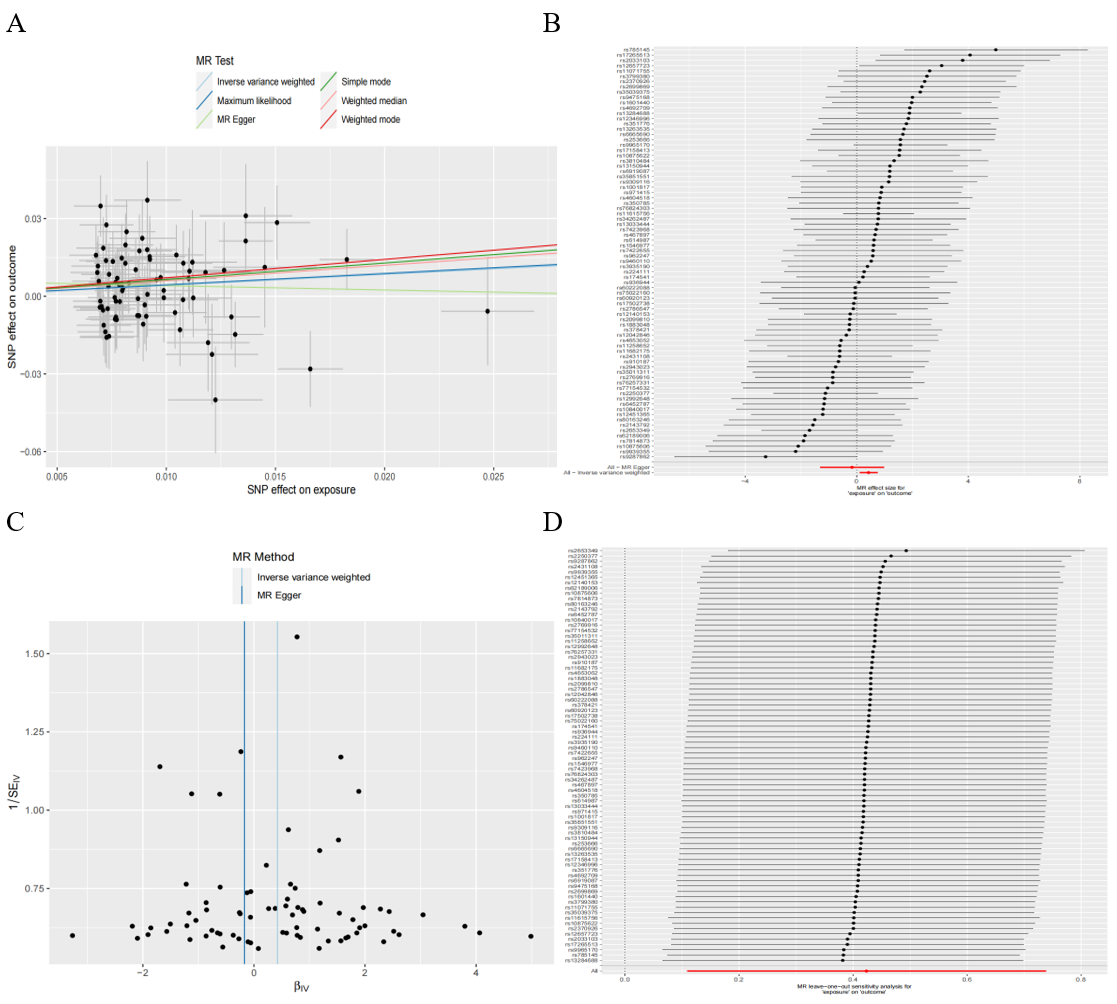
**

**Figure S98 Scatter plot (A), forest plot (B), funnel plot (C) and sensitivity analysis (D) of the causal effect of insomnia on *Ruminococcaceae UCG013*.**

**
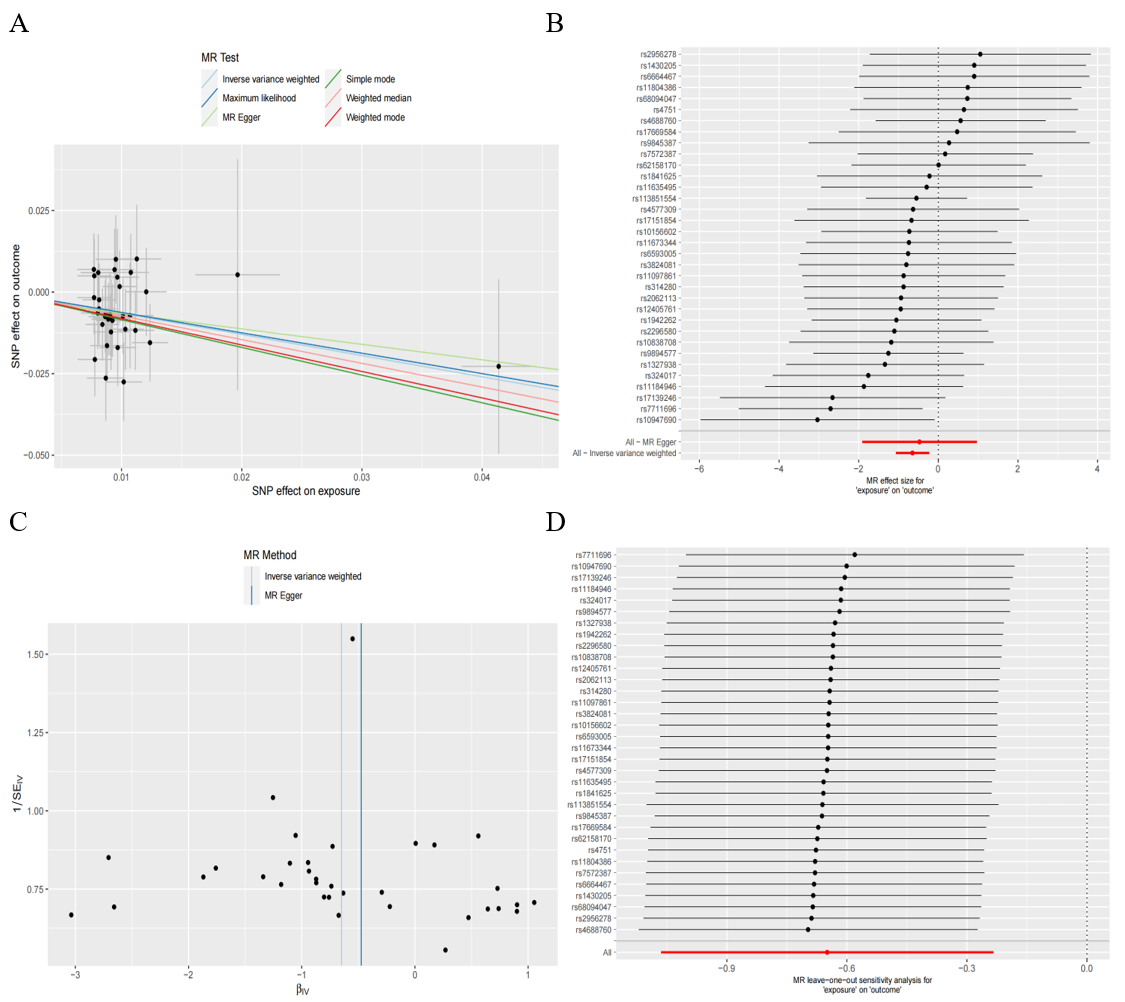
**

**Figure S99 Scatter plot (A), forest plot (B), funnel plot (C) and sensitivity analysis (D) of the causal effect of chronotype on *Butyricicoccus*.**

**
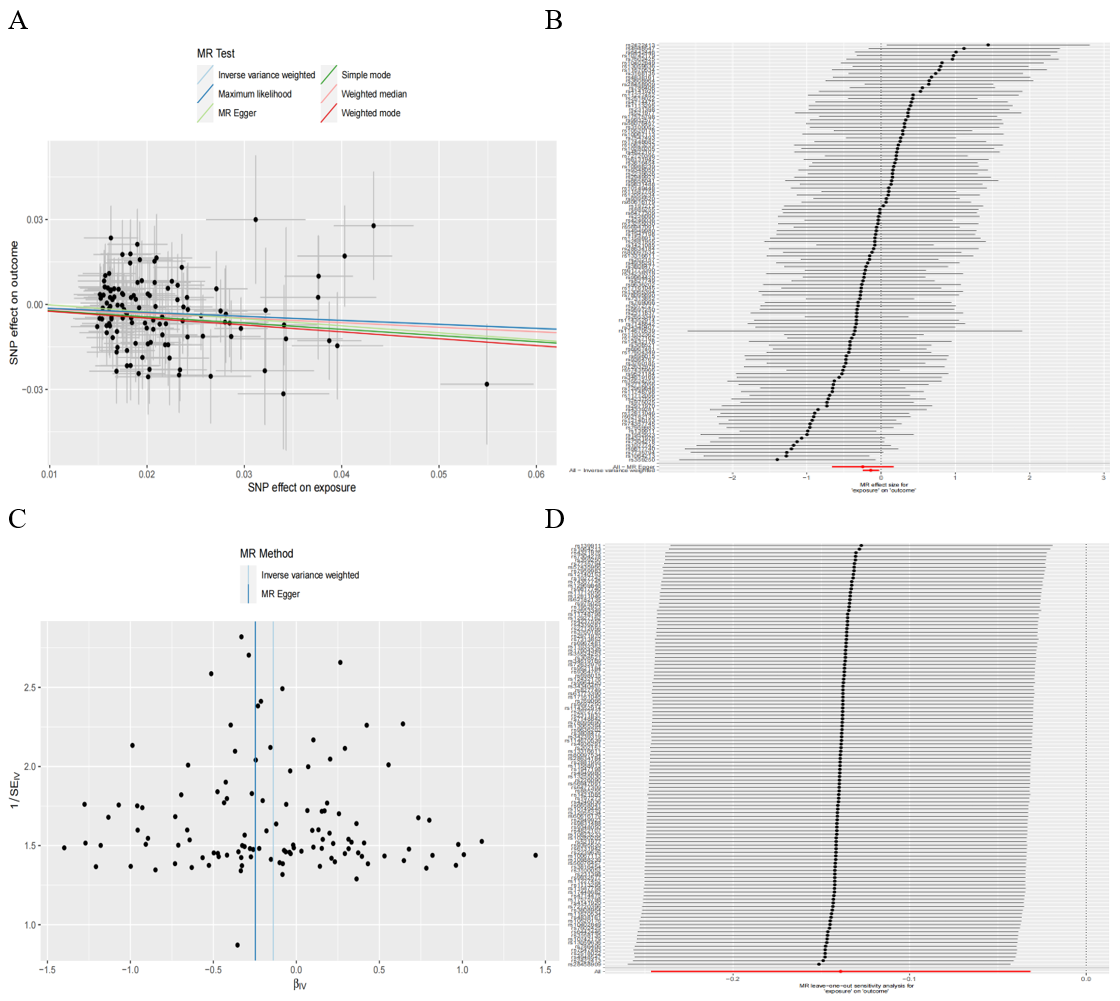
**

**Figure S100 Scatter plot (A), forest plot (B), funnel plot (C) and sensitivity analysis (D) of the causal effect of insomnia on *Clostridiumsensustricto 1*.**

**
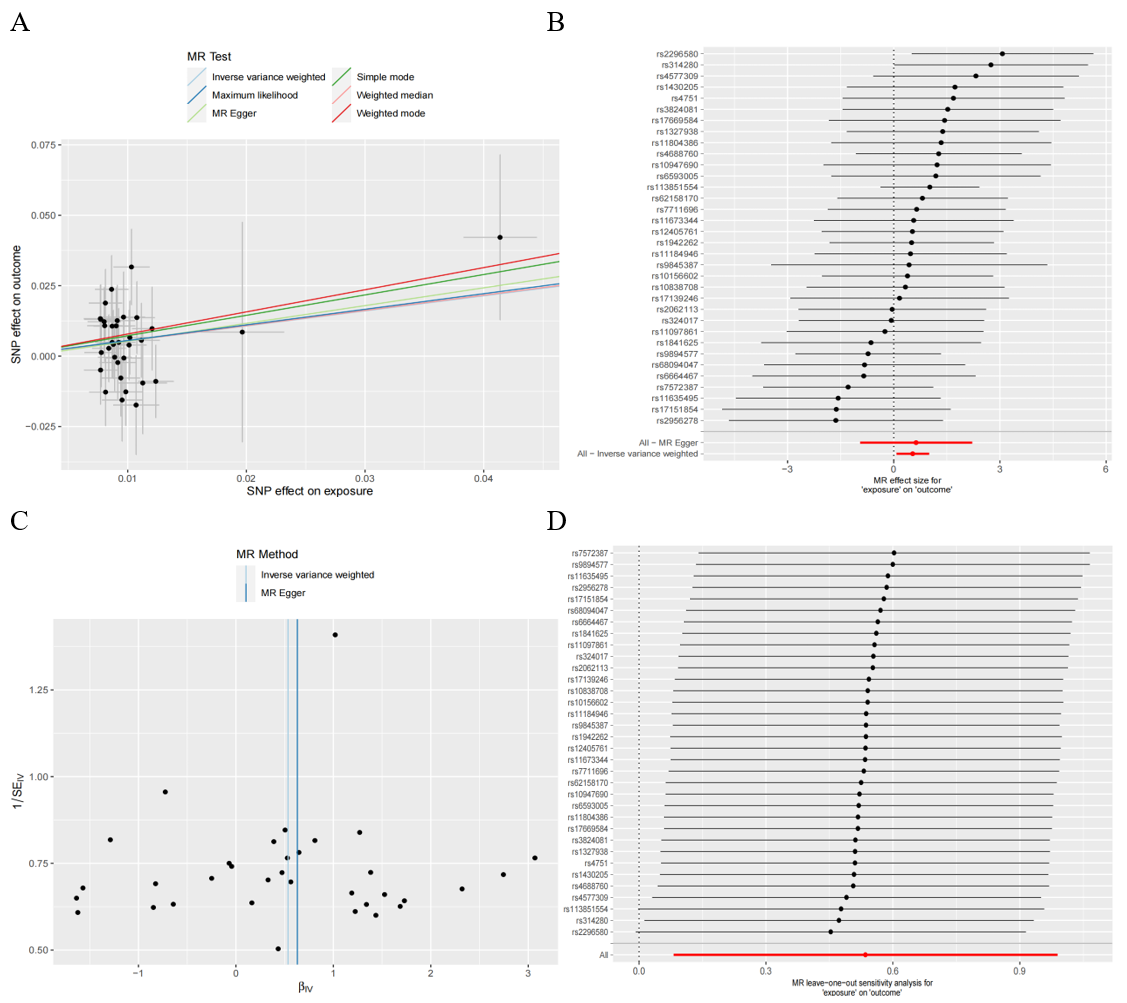
**

**Figure S101 Scatter plot (A), forest plot (B), funnel plot (C) and sensitivity analysis (D) of the causal effect of short sleep duration on *Eubacterium oxidoreducens group*.**

**Figure S102 Scatter plot (A), forest plot (B), funnel plot (C) and sensitivity analysis (D) of the causal effect of sleep duration on *Eubacterium ventriosum group*.**

**Figure S103 Scatter plot (A), forest plot (B), funnel plot (C) and sensitivity analysis (D) of the causal effect of short sleep duration on *Eubacterium xylanophilum group*.**

**Figure S104 Scatter plot (A), forest plot (B), funnel plot (C) and sensitivity analysis (D) of the causal effect of insomnia on *Eubacterium nodatum group*.**

**Figure S105 Scatter plot (A), forest plot (B), funnel plot (C) and sensitivity analysis (D) of the causal effect of daytime sleepiness on *Blautia*.**

**Figure S106 Scatter plot (A), forest plot (B), funnel plot (C) and sensitivity analysis (D) of the causal effect of insomnia on *Butyrivibrio*.**

**Figure S107 Scatter plot (A), forest plot (B), funnel plot (C) and sensitivity analysis (D) of the causal effect of daytime sleepiness on *Coprococcus 3*.**

**Figure S108 Scatter plot (A), forest plot (B), funnel plot (C) and sensitivity analysis (D) of the causal effect of daytime sleepiness on *Dorea*.**

**Figure S109 Scatter plot (A), forest plot (B), funnel plot (C) and sensitivity analysis (D) of the causal effect of daytime napping on *Fusicatenibacter*.**

**Figure S110 Scatter plot (A), forest plot (B), funnel plot (C) and sensitivity analysis (D) of the causal effect of short sleep duration on *Sellimonas*.**

**Figure S111 Scatter plot (A), forest plot (B), funnel plot (C) and sensitivity analysis (D) of the causal effect of sleep duration on *Eubacterium rectale group*.**

**Figure S112 Scatter plot (A), forest plot (B), funnel plot (C) and sensitivity analysis (D) of the causal effect of long sleep duration on *Ruminococcus gnavus group*.**

**Figure S113 Scatter plot (A), forest plot (B), funnel plot (C) and sensitivity analysis (D) of the causal effect of chronotype on *Ruminococcus gnavus group*.**

**Figure S114 Scatter plot (A), forest plot (B), funnel plot (C) and sensitivity analysis (D) of the causal effect of daytime sleepiness on *Anaerofilum*.**

**Figure S115 Scatter plot (A), forest plot (B), funnel plot (C) and sensitivity analysis (D) of the causal effect of daytime sleepiness adjusted for BMI on *Anaerofilum*.**

**Figure S116 Scatter plot (A), forest plot (B), funnel plot (C) and sensitivity analysis (D) of the causal effect of binary chronotype on *Faecalibacterium*.**

**Figure S117 Scatter plot (A), forest plot (B), funnel plot (C) and sensitivity analysis (D) of the causal effect of daytime sleepiness on *Flavonifractor*.**

**Figure S118 Scatter plot (A), forest plot (B), funnel plot (C) and sensitivity analysis (D) of the causal effect of daytime sleepiness adjusted for BMI on *Flavonifractor*.**

**Figure S119 Scatter plot (A), forest plot (B), funnel plot (C) and sensitivity analysis (D) of the causal effect of chronotype on *Ruminiclostridium 6*.**

**Figure S120 Scatter plot (A), forest plot (B), funnel plot (C) and sensitivity analysis (D) of the causal effect of sleep duration on *Catenibacterium*.**

**Figure S121 Scatter plot (A), forest plot (B), funnel plot (C) and sensitivity analysis (D) of the causal effect of long sleep duration on *Allisonella*.**

**Figure S122 Scatter plot (A), forest plot (B), funnel plot (C) and sensitivity analysis (D) of the causal effect of daytime sleepiness on *Dialister*.**

**Figure S123 Scatter plot (A), forest plot (B), funnel plot (C) and sensitivity analysis (D) of the causal effect of short sleep duration on *Desulfovibrio*.**
